# OMA1 mediates local and global stress responses against protein misfolding in CHCHD10 mitochondrial myopathy

**DOI:** 10.1172/JCI157504

**Published:** 2022-07-15

**Authors:** Mario K. Shammas, Xiaoping Huang, Beverly P. Wu, Evelyn Fessler, Insung Y. Song, Nicholas P. Randolph, Yan Li, Christopher K.E. Bleck, Danielle A. Springer, Carl Fratter, Ines A. Barbosa, Andrew F. Powers, Pedro M. Quirós, Carlos Lopez-Otin, Lucas T. Jae, Joanna Poulton, Derek P. Narendra

**Affiliations:** 1 Inherited Movement Disorders Unit, Neurogenetics Branch, National Institute of Neurological Disorders and Stroke (NINDS), NIH, Bethesda, Maryland, USA.; 2 Gene Center and Department of Biochemistry, Ludwig-Maximilians-Universität München, Munich, Germany.; 3 Proteomics Core Facility, National Institute of Neurological Disorders and Stroke and; 4 National Heart, Lung, and Blood Institute, (NHLBI) NIH, Bethesda, Maryland, USA.; 5 Oxford Genetics Laboratories, Oxford University Hospitals NHS Foundation Trust, Oxford, United Kingdom.; 6 Department of Medical and Molecular Genetics, School of Basic and Medical Biosciences, King’s College London, United Kingdom.; 7 Ionis Pharmaceuticals, Carlsbad, California, USA.; 8 Departamento de Bioquímica y Biología Molecular, Facultad de Medicina, Instituto Universitario de Oncología, Universidad de Oviedo, Oviedo, Spain.; 9 Nuffield Department of Women’s and Reproductive Health, University of Oxford, Oxford, United Kingdom.

**Keywords:** Cell Biology, Genetics, Cell stress, Mitochondria, Proteases

## Abstract

Mitochondrial stress triggers a response in the cell’s mitochondria and nucleus, but how these stress responses are coordinated in vivo is poorly understood. Here, we characterize a family with myopathy caused by a dominant p.G58R mutation in the mitochondrial protein CHCHD10. To understand the disease etiology, we developed a knockin (KI) mouse model and found that mutant CHCHD10 aggregated in affected tissues, applying a toxic protein stress to the inner mitochondrial membrane. Unexpectedly, the survival of CHCHD10-KI mice depended on a protective stress response mediated by the mitochondrial metalloendopeptidase OMA1. The OMA1 stress response acted both locally within mitochondria, causing mitochondrial fragmentation, and signaled outside the mitochondria, activating the integrated stress response through cleavage of DAP3-binding cell death enhancer 1 (DELE1). We additionally identified an isoform switch in the terminal complex of the electron transport chain as a component of this response. Our results demonstrate that OMA1 was critical for neonatal survival conditionally in the setting of inner mitochondrial membrane stress, coordinating local and global stress responses to reshape the mitochondrial network and proteome.

## Graphical abstract

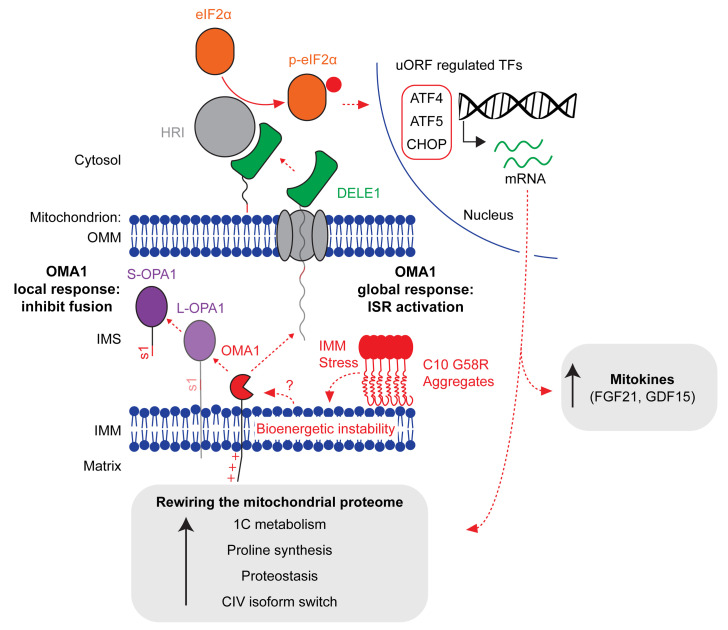

## Introduction

Mitochondria produce energy by coupling the transport of protons across the inner mitochondrial membrane (IMM) to the production of ATP (1). The integrity of the IMM is sensed by quality control pathways that include the IMM metalloendopeptidase OMA1 and the PINK1/PARKIN pathway (2–5).

OMA1 senses IMM integrity through its N-terminus, which responds to loss of IMM voltage by increasing OMA1 peptidase activity (6). Once activated by loss of IMM voltage, OMA1 inhibits the fusion of dysfunction mitochondria by cleaving the IMM fusion protein OPA1 (L-OPA1), as part of a local response (2, 3, 7). Recently, OMA1 was additionally found to mediate a global stress response, communicating bioenergetic stress to the nucleus through the integrated stress response (ISR) in cultured cells (8, 9). Along this axis, OMA1 cleaves DAP3-binding cell death enhancer 1 (DELE1) in the process of mitochondrial import, thereby releasing a short form of DELE1 to the cytosol to activate HRI (10), one of four eIF2α kinases. eIF2α phosphorylation inhibits the cytosolic translation of most mRNAs but increases the translation of select mRNAs that contain upstream ORFs, notably the transcription factors ATF4, ATF5, and CHOP. These transcription factors upregulate amino acid transporters, metabolic enzymes, and other transcription factors to restore cellular homeostasis. Although mitochondrial stress leads to an ATF4 transcriptional response in diverse models of mitochondrial disease (11–17), it is not known whether OMA1 activation can mediate mitonuclear communication in vivo. Additionally, it is not known whether OMA1 activation may protect against specific mitochondrial stresses in vivo, as prior in vivo studies have found that OMA1 activation is often maladaptive (18, 19).

We recently discovered that OMA1 is activated in response to pathogenic mutations in the protein CHCHD10 (hereafter referred to as C10) (15). C10 is a small mitochondrial intermembrane space (IMS) protein that extends into mitochondrial cristae and, together with its paralog CHCHD2 (hereafter referred to as C2), is important for maintaining inner membrane integrity and electron transport chain (ETC) function (15, 20–24). Dominant mutations in C10 cause a spectrum of neuromuscular disorders that includes autosomal dominant isolated mitochondrial myopathy (IMMD, reported family with R15S and G58R variants in *cis*) (25, 26), myopathy with amyotrophic lateral sclerosis/frontotemporal dementia (ALS/FTD; S59L) (27), adult-onset spinal muscular atrophy (SMA-J; G66V) (28), and ALS (R15L) (29), whereas the T61I mutation in C2 causes Parkinson’s disease (30). It is unresolved which C10 mutation, R15S or G58R, causes IMMD, as the 2 mutations cosegregated with disease in the 1 reported family (hereafter referred to as the US family).

Here, we present a study of a family with myopathy and cardiomyopathy (hereafter referred to as the UK family) due to the C10 G58R mutation and demonstrate that the G58R mutation is the cause of IMMD. This phenotype was also observed in what we believe to be the first C10^G58R/+^ (hereafter referred to as C10^G58R^) knockin (KI) mouse model. We further identify that OMA1 was critical for C10^G58R^ neonatal survival, as it orchestrated both local and global stress responses to IMM stress from misfolding of the C10 protein.

## Results

### The CHCHD10 G58R mutation causes autosomal dominant myopathy and cardiomyopathy.

**Figure 1:**
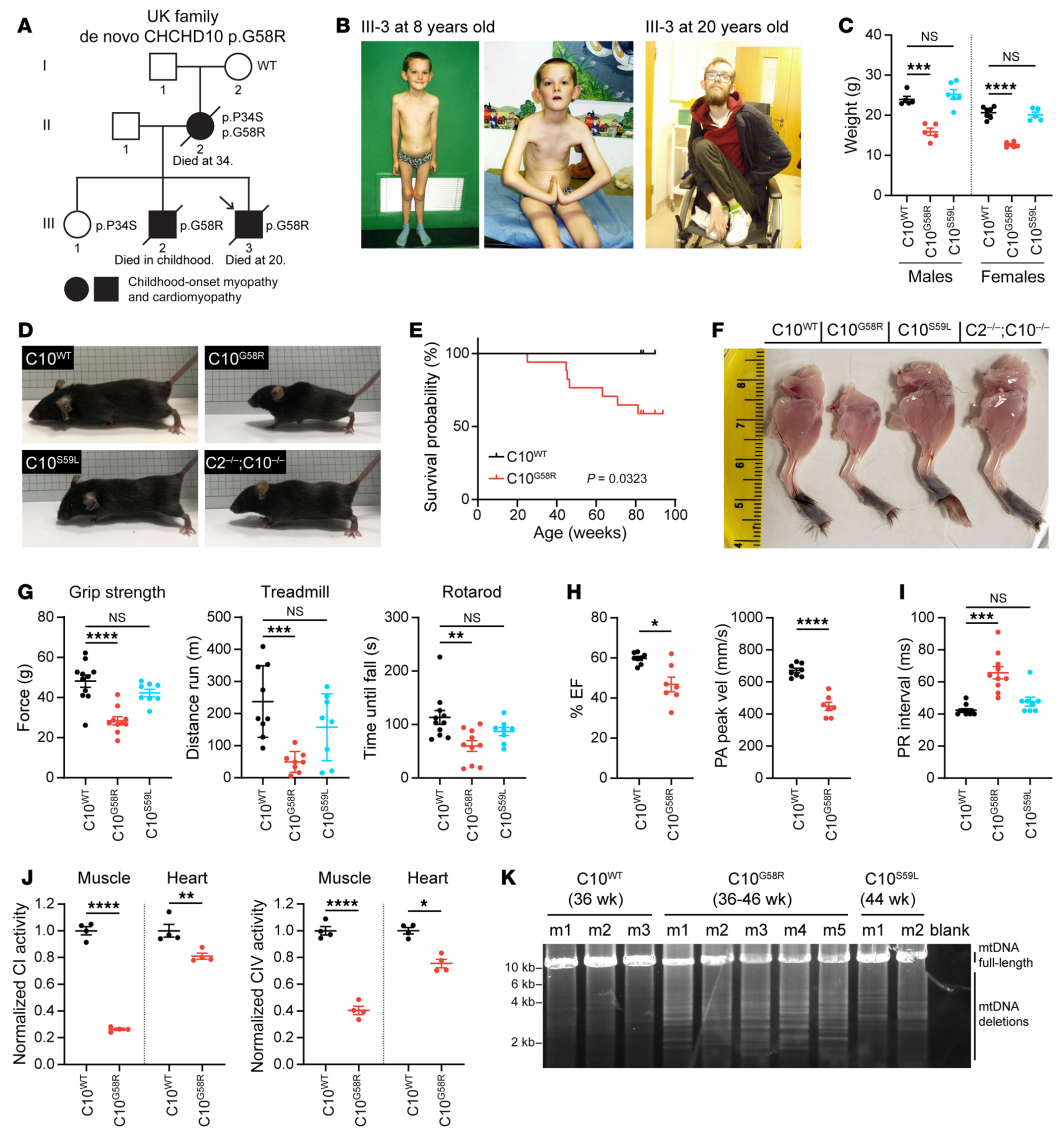
The C10 G58R mutation causes dominant myopathy and cardiomyopathy in humans and mice. (**A**) Pedigree of the UK family. Arrow indicates the proband. (**B**) The proband (III-3) at 8 (left) and 20 (right) years of age. (**C**) Weights of 10- to 11-week-old C10^WT^, C10^G58R^, and C10^S59L^ male and female mice. (**D**) Fifteen-week-old C10^WT^ and C10^G58R^ littermate mice, with C10^S59L^ and C2/C10-DKO mice of the same age for comparison. (**E**) Survival curve for C10^WT^ (*n =* 9) and C10^G58R^ (*n =* 17) mice. (**F**) Skinned hind limbs of 15-week-old C10^WT^, C10^G58R^, C10^S59L^, and C2/C10-DKO mice. (**G**) Grip strength, treadmill fatigue test, and rotarod assays of 18-week-old C10^WT^ and C10^G58R^ mice and 25-week-old C10^S59L^ mice. (**H**) Ejection fraction (EF) percentage and pulmonary artery (PA) peak velocity (vel) in C10^WT^ and C10^G58R^ mice on echocardiography. (**I**) Electrocardiographic PR intervals for C10^WT^, C10^G58R^, and C10^S59L^ mice. (**J**) CI and CIV activity in C10^WT^ and C10^G58R^ mouse heart and tibialis lysates. (**K**) Long-range PCR of a 12.8 kb segment of C10^WT^, C10^G58R^, and C10^S59L^ mouse heart mtDNA. m, mouse. Error bars represent the SEM. *P < 0.05, **P < 0.01, ***P < 0.001, and ****P < 0.0001, by 1-way ANOVA with Dunnett’s T3 multiple comparisons (**C**, **G**, and **I**), log-rank test (**E**), and *t* test with Welch’s correction (**H** and **J**). See also Supplemental Figures 1–3, Table 1, and Supplemental data set 1.

We identified a family with autosomal dominant mitochondrial myopathy and cardiomyopathy (Figure 1A). The mother of the UK family was previously described approximately 50 years ago as having childhood-onset myopathy with mitochondrial inclusions of unknown cause (31). Electromyography (EMG) showed only myopathic features, with no fibrillation potentials at rest (31). The proband also had childhood-onset myopathy with delayed motor milestones, an inability to run or jump, a positive Gowers’s sign, frequent falls, and lax ligaments (Figure 1B). By 20 years of age, the proband was wheelchair bound and had received a heart transplant because of severe progression of his cardiomyopathy. He died from lymphoma soon afterwards, probably as a complication of immunosuppression. The proband had an unaffected sister (III-1) and a brother with myopathy and cardiomyopathy who died in childhood (III-2).

Whole-exome sequencing demonstrated a p.G58R mutation in the proband, and Sanger sequencing of C10 in other members of the UK family revealed the p.G58R variant in *trans*, with the benign p.P34S variant in the mother, the benign p.P34S variant in the unaffected daughter, and the p.G58R variant in the affected brother. The maternal grandmother had neither the p.G58R nor the p.P34S variant, suggesting that the mother most likely inherited the p.P34S variant from the maternal grandfather and that the p.G58R variant most likely arose in the mother de novo. Together, these findings establish that the G58R variant is pathogenic and suggest that it primarily affects striated muscle in humans.

Our assessment of muscle tissue from members of the UK family was also consistent with mitochondrial myopathy and similar to findings reported for the US family (25, 26). A biopsy of the proband’s pectoralis muscle at 8 years of age showed excess lipid droplets in type I fibers, suggestive of a mitochondrial myopathy (Supplemental Figure 1A; supplemental material available online with this article; https://doi.org/10.1172/JCI157504DS1). Mitochondrial DNA (mtDNA) deletions were present at low levels in the pectoralis muscle but not the heart (detectable by long-range PCR but not by Southern blotting) (Supplemental Figure 1B). Mitochondrial complex activity assays of the proband’s muscle showed normal complex I activity, moderately decreased complex II-III activity, and more severely decreased complex IV activity (Table 1). Additionally, the mother’s muscle biopsy showed a predominance of type I fibers, with ragged-red fibers and cytochrome c oxidase–negative (COX^–^) fibers (Supplemental Figure 1C). The proband also had substantially elevated serum FGF-21 (5000 pg/mL; 3.89 SDs above the mean for his age), which was previously published as part of a large series (32).

To further study the pathogenesis of C10 G58R, we assessed mouse C10 with the homologous mutation (p.G54R in mouse, but the human numbering will be used throughout). We first confirmed that mouse C10 and human C10 are functionally conserved, using a cell-based C2/C10 complementation assay that we developed previously (15). Mouse C10 was able to suppress OMA1 activation in cells lacking human C2 and C10, as evidenced by partial normalization of OPA1 cleavage (Supplemental Figures 1, D and E). Additionally, mouse C10 G58R exogenous expression caused mitochondrial fragmentation in human cells, similar to what we observed previously for human C10 G58R (Supplemental Figures 1, F and G). Together, these findings indicate that human and mouse C10 are functionally conserved.

We next generated a C10^G58R^-KI mouse model (Supplemental Figure 2A). The C10^G58R^ mice were smaller, had decreased body weight, and died prematurely (Figure 1, C–E). The weight difference for C10^G58R^ mice was already clear at the time of weaning (Supplemental Figure 2B). By contrast, C10^S59L/+^ (hereafter referred to as C10^S59L^) and C2/C10 double-KO (DKO) mice were similar in size to WT mice at 15 weeks (Figure 1D). Total food intake did not differ between C10^G58R^ and C10^WT^ mice, and there was no difference in the percentage of lean and fat mass between the genotypes (Supplemental Figure 2, C and D). The respiratory exchange ratio, measured by a comprehensive laboratory animal monitoring system (CLAMS), tended to be higher for C10^G58R^ mice at night (indicating increased carbohydrate reliance) and lower during the day (indicating increased fatty acid reliance) compared with that of C10^WT^ littermates, although these differences did not reach statistical significance (Supplemental Figure 2E). When accounting for body weight, we found no difference between C10^WT^ and C10^G58R^ mice in oxygen consumption, carbon dioxide production, or energy expenditure (Supplemental Figure 2F).

Similar to the proband, C10^G58R^ mice had myopathy, with smaller leg muscles compared with C10^WT^, C10^S59L^, and C2/C10-DKO mice (Figure 1F). Consistently, muscle fiber size in C10^G58R^ mice was smaller than in C10^WT^ mice (Supplemental Figures 3, A and B), but no COX^–^ fibers were observed (Supplemental Figure 3A). The myopathy was also functionally evident in C10^G58R^ mice, with decreased grip strength, increased treadmill-induced fatigue, and worse rotarod performance when tested at 18 weeks (Figure 1G). C10^S59L^ mice tested at a slightly older age (25 weeks) did not yet differ from C10^WT^ mice (Figure 1G), although both C10^G58R^ and C10^S59L^ mice took longer to descend a 50 cm pole (Supplemental Figure 3C). Additionally, C10^G58R^ (but not C10^S59L^) mouse tibialis muscles had increased lipid droplets, phenocopying findings in muscle biopsies from members of the UK family (Supplemental Figure 3D). Also similar to the proband, C10^G58R^ mice had decreased heart function as measured by echocardiography (Figure 1H and Supplemental data set 1) and, additionally, had atrioventricular heart block (Figure 1I and Supplemental Figure 3E). Consistent with a mitochondrial basis for heart and muscle dysfunction, complex I and complex IV activities were diminished in C10^G58R^ mouse heart and muscle (Figure 1J), and C10^G58R^ mice had multiple mtDNA deletions and decreased mtDNA copy numbers in the heart, suggesting a mild defect in mtDNA maintenance (Figure 1K and Supplemental Figure 3F). Together, these results demonstrate that the C10^G58R^ mouse model recapitulated the myopathy and cardiomyopathy phenotypes of the UK family and had a more severe myopathy than did the C10^S59L^ mouse model.

### CHCHD10 G58R and S59L mutations form distinct aggregates.

**Figure 2:**
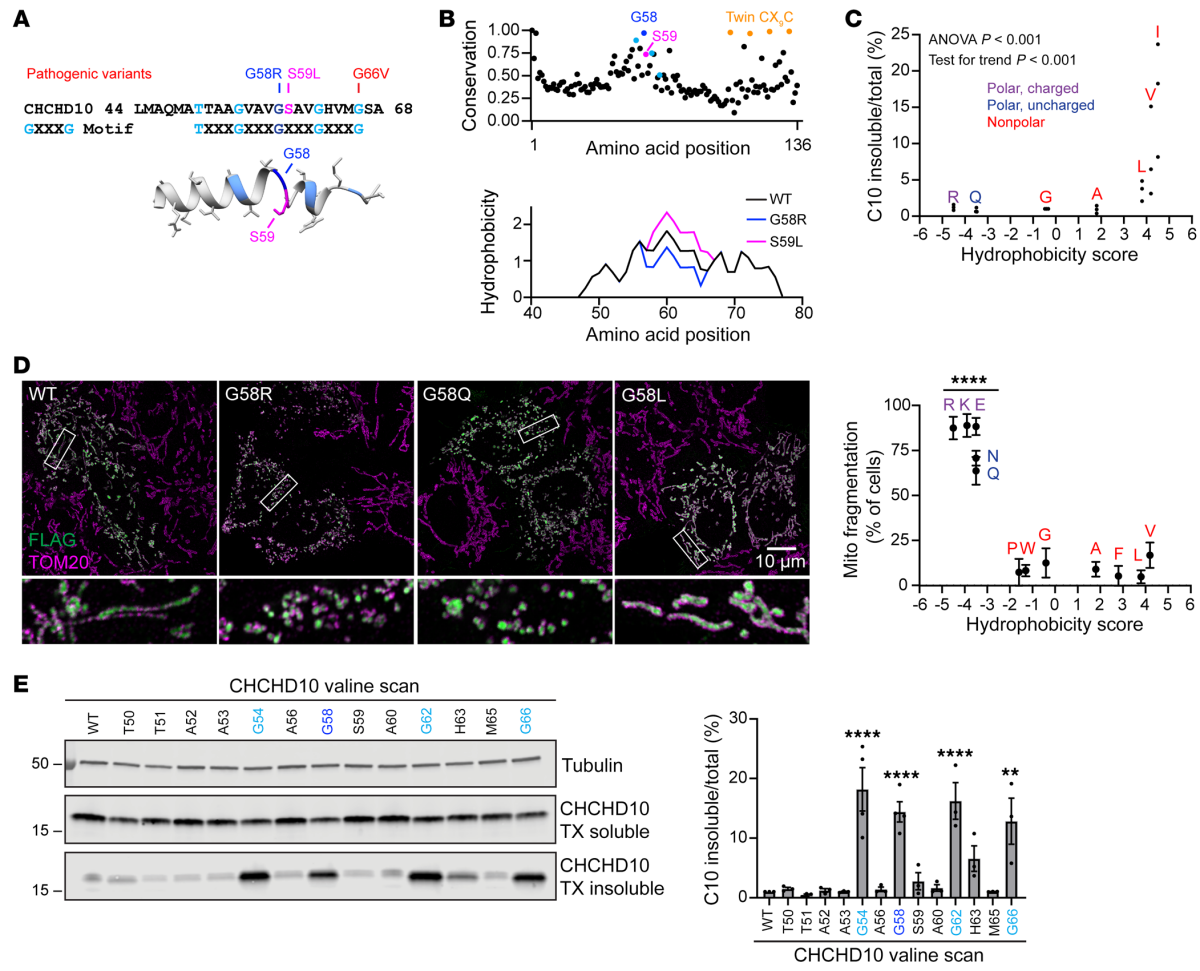
The C10 G58 position is highly conserved and lies on an insolubility-prone face of the α-helix. (**A**) Top: Amino acid sequence of the hydrophobic α-helix of C10 showing pathogenic variants and highlighting the GXXXG motif. Bottom: EVfold prediction of the structure of the α-helix. (**B**) EVcouplings conservation analysis of C10 and predicted hydrophobicity of the region around the α-helix. (**C**) Levels of insoluble/total C10 in HEK293 C2/C10-DKO cells after transfection with C10 containing G58 substitutions with amino acids of varying hydrophobicity on the Kyte-Doolittle scale (*x* axis) (*n =* 3 biological replicates). (**D**) Representative Airyscan images of mitochondria in HeLa cells transfected with C10 containing the indicated G58 substitutions. Scale bar: 10 μm. Original magnification, ×5 (bottom images). Plot shows quantification of mitochondrial (Mito) fragmentation in HeLa cells transfected with C10 containing G58 substitutions with amino acids of varying hydrophobicity (*n* ≥50 cells per replicate from 3 biological replicates; individual data points are shown in Supplemental Figure 4B). (**E**) Representative blot of a Triton X–soluble/–insoluble (TX-soluble/-insoluble) assay of HEK293 C2/C10-DKO cells transfected with C10, whereby individual residues of the α-helix were mutated to valines. Graph shows quantification of the blots (*n =* 3–4 biological replicates). Error bars represent the SEM. **P < 0.01 and ****P < 0.0001, by 1-way ANOVA with Dunnett’s T3 multiple comparisons with pooled variance (**D**) or Sidak’s multiple comparisons (**E**). See also Supplemental Figure 4.

We next explored the basis for phenotypic differences between C10^G58R^ and C10^S59L^ mice. The G58 and S59 residues are highly conserved and are predicted to introduce a break in the central α-helix (Figure 2, A and B). Notably, although the S59L mutation increased the calculated hydrophobicity of the region, as recognized previously (11), the G58R mutation decreased it, suggesting that mutations in these neighboring residues may have differential physiochemical effects on the α-helix (Figure 2B, bottom).

Consistent with its conservation, we found that the G58 residue was intolerant to substitution when exogenously expressed in cultured cells. Hydrophobic substitutions decreased C10 protein solubility (Figures 2C and Supplemental Figure 4A), whereas large hydrophilic substitutions caused mitochondrial fragmentation, similar to what we and others previously reported for the pathogenic G58R substitution (Figure 2D and Supplemental Figure 4B) (15, 23, 25). An arginine scan of this region showed that the G58 position was the most prone to causing mitochondrial fragmentation (Supplemental Figure 4C). We previously observed that the neuronopathy-causing G66V mutation also causes C10 insolubility (23). As the G58 and G66 residues lie on either end of a GXXXGXXXG motif, we systematically assessed the effect of valine substitutions on C10 solubility. Notably, substitution of any glycine residue strongly reduced C10 solubility (Figure 2E; see complete unedited blots in the supplemental material). Thus, increasing hydrophobicity of the glycine face of the α-helix may promote an insoluble C10 conformation. This contrasts with the C10 G58R substitution, which may adopt a soluble conformation that is more prone to inducing mitochondrial fragmentation.

**Figure 3:**
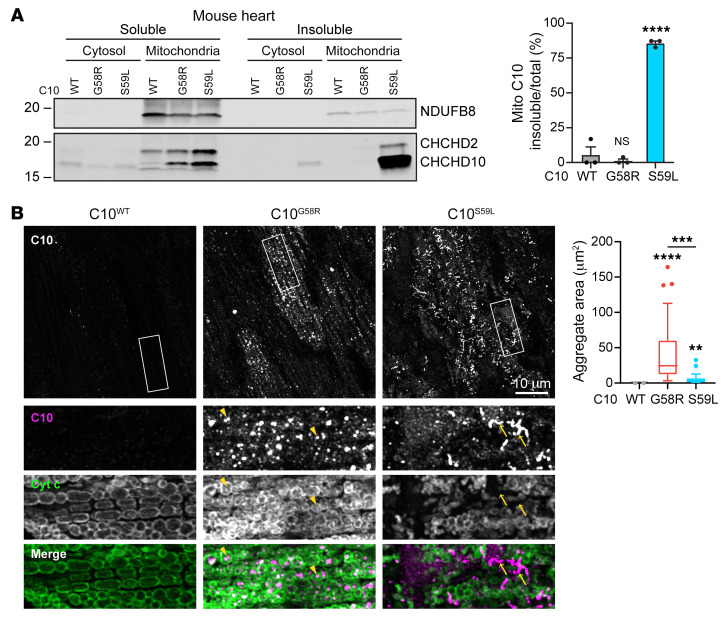
C10 G58R is predominantly soluble but forms aggregates that are distinct from C10 S59L. (**A**) Representative immunoblot of a soluble/insoluble assay of C10 from the cytosolic or mitochondrial (Mito) fraction of C10^WT^, C10^G58R^, and C10^S59L^ mouse hearts. Loading controls are shown in Supplemental Figure 4D. Graph shows quantification of the blots (*n =* 3 mice per genotype). (**B**) Airyscan images of 15-week-old C10^WT^, C10^G58R^, and C10^S59L^ mouse hearts stained for C10 and cytochrome c. Arrowheads show intramitochondrial aggregates, and arrows show extramitochondrial aggregates. Quantification of C10 aggregate area per field of view (FOV) of 36- to 46-week-old mice (*n =* 3 mice C10^WT^ and C10^S59L^, *n =* 4 mice C10^G58R^; *n* = 8 FOV per mouse). Scale bar: 10 μm. Original magnification, ×2.5 (bottom images). Error bars represent the SEM. **P < 0.01, ***P < 0.001, and ****P < 0.0001, by 1-way ANOVA with Dunnett’s T3 multiple comparisons. Box-and-whisker plot lines were calculated using Tukey’s method. See also Supplemental Figure 4.

The S59L mutation was previously reported to form insoluble C10 aggregates in mouse heart (11, 15), so we compared the solubility and aggregation tendency of the G58R and S59L mutations in this tissue. In the hearts of all animals, we found that C10 was predominantly in the mitochondrial fraction, suggesting that the G58R and S59L mutations do not alter the mitochondrial targeting of C10 (Figure 3A and Supplemental Figure 4D). Consistent with the data from cultured cells and the calculated change in hydrophobicity, C10 with the G58R mutation was soluble, in contrast to C10 with the S59L mutation (Figure 3A and Supplemental Figure 4D). C10 levels were much higher in C10^S59L^ than in C10^G58R^ mouse hearts, raising the possibility that insolubility may drive the increased protein level of C10 S59L. C10 protein aggregates were prominent in C10^G58R^ mouse hearts, but had a distinct morphology and localization compared with those in C10^S59L^ mouse hearts (Figure 3B and Supplemental Figure 4E). While C10 S59L protein tended to have a filamentous morphology, C10 G58R protein formed punctate aggregates (Figure 3B and Supplemental Figure 4E). C10 G58R aggregates were mostly within the mitochondrial boundary delineated by cytochrome c immunofluorescence and colocalized with the mitochondrial matrix protein PDH. By contrast, C10 S59L aggregates appeared to be outside of mitochondria. Additionally, C10 G58R aggregates occupied a larger area of the tissue in heart and skeletal muscle (Figure 3B and Supplemental Figure 4F). Together, these findings suggest that C10 G58R and S59L proteins likely form distinct aggregates, which may reflect their different conformations and toxicities.

### Intracristal inclusions are characteristic of C10 G58R pathology and likely reflect inner membrane stress.

**Figure 4:**
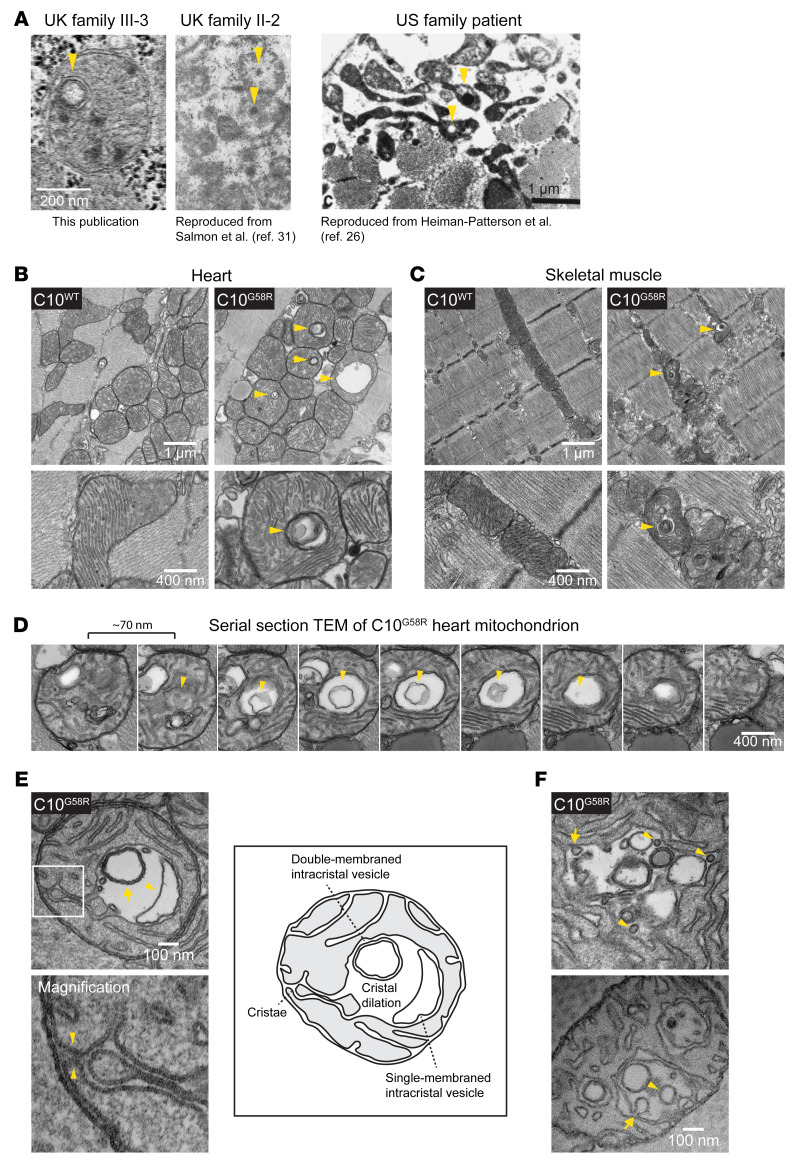
Intracristal inclusions are characteristic of affected C10 G58R patient and mouse muscle. (**A**) Intramitochondrial inclusions in muscles of patients with the G58R mutation (arrowheads). Scale bar: 1 μm. The “UK family II-2” image was reproduced with permission from *The Lancet* (ref. 31), and the “US family patient” image was reproduced with permission from *Muscle & Nerve* (ref. 26). (**B**) TEM of 28-week-old C10^WT^ and C10^G58R^ mouse hearts. Arrowheads indicate intramitochondrial inclusions (representative of ≥15 fields in 2 biological replicates). (**C**) TEM of tibialis from 14-week-old C10^WT^ and C10^G58R^ mice on the OMA1^+/–^ background. Arrowheads indicate intramitochondrial inclusions (representative of ≥10 fields in 1 biological replicate). Scale bars: 1 μm and 400 nm (**B** and **C**). (**D**) Serial TEM following an intramitochondrial inclusion containing a membranous vesicle within (representative of ≥6 mitochondria from 1 biological replicate). Scale bar: 400 nm. (**E**) Top: TEM of an ultrathin section of a 33-week-old C10^G58R^ mouse heart showing cristal dilation, a single-membraned intracristal vesicle (arrowhead), and a double-membraned intracristal vesicle (arrow). Bottom: Higher-magnification (×3.5) image showing continuity with the IMM, indicated by arrowheads. Right: Sketch and labels for the image on the left (representative of ≥10 mitochondria in 1 biological replicate). (**F**) TEM of an ultrathin section of a 33-week-old C10^G58R^ mouse heart showing inner membrane active budding events (arrows) and completed budding (arrowheads) (representative of ≥5 mitochondria in 1 biological replicate). Scale bar: 100 μm.

Double-membraned inclusions within mitochondria were observed in the proband’s muscle biopsy and were similar in appearance to the “inclusion bodies” previously noted in his mother and the “globular inclusions“ previously noted in a member of the US family (Figure 4A) (26, 31). In C10^G58R^ mouse heart and skeletal muscle (but not C10^WT^ or C10^S59L^ tissues), we observed similar inclusions in nearly every field of view (Figure 4, B and C). These inclusions were enclosed within mitochondria, as demonstrated by serial EM (Figure 4D), and were formed from the cristal membrane with which they were continuous (Figure 4E). Thus, they represented dilations of cristae, which sometimes contained membranous intracristal vesicles with single or multiple membranes (Figure 4E). Additionally, we observed possible inward budding events of the cristal membrane into the intracristal space, suggesting that at least some of the internal membranous structures may be derived from the cristal membrane (Figure 4F). Together, these observations establish that intracristal inclusions are characteristic of C10 G58R myopathy.

### OMA1 is activated by IMM stress in C10^G58R^ mice.

We reasoned that the cristal inclusions in C10^G58R^ striated muscle may be symptomatic of inner membrane stress exerted by C10 G58R protein misfolding in the IMS. We next assessed whether OMA1, a peptidase monitoring IMM stress, might be activated by the G58R mutation in vivo, similar to what we reported previously for the G58R mutation in cell culture and the S59L mutation in vivo (15).

**Figure 5:**
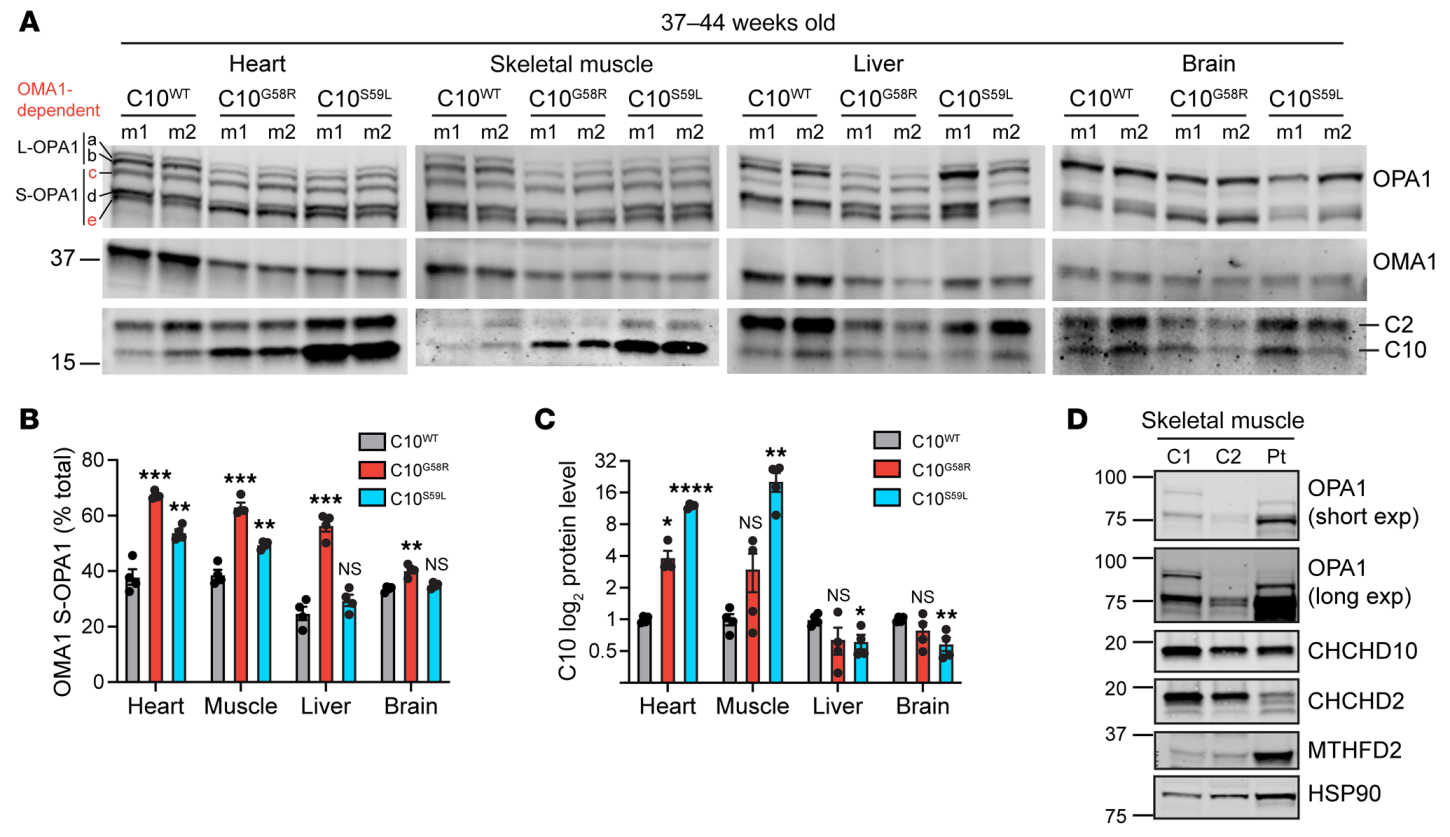
OMA1 is activated in C10 G58R mouse tissue. (**A**) Immunoblots of OPA1, OMA1, C2, and C10 in different tissues from 37- to 44-week-old C10^WT^, C10^G58R^, and C10^S59L^ mice. The loading controls are shown in Supplemental Figure 5A. (**B**) Quantification of OMA-cleaved OPA1 bands (c+e/total) from **A** (*n =* 4 mice per genotype). (**C**) Quantification of C10 levels from **A**. (**D**) Immunoblot of OPA1 levels from the C10 G58R proband (patient III-3) and 2 nonmyopathic controls. C, control, exp, exposure; Pt, patient. Error bars represent the SEM. *P < 0.05, **P < 0.01, ***P < 0.001, and ****P < 0.0001, by 1-way ANOVA with Dunnett’s T3 multiple comparisons. See also Supplemental Figure 5.

OMA1 activity was increased in C10^G58R^ mice in all tissues examined, as reflected by a decrease in L-OPA1 (bands a and b) and an increase in OMA1-generated short forms (S-OPA1, bands c and e) (Figure 5, A and B, and Supplemental Figure 5A). OMA1 was more strongly activated by C10 G58R than by C10 S59L in all tissues, despite higher protein levels of C10^S59L^ in some of these tissues (Figure 5, A and C). C2 protein levels trended toward an increase in mutant hearts but did not reach significance (Figure 5A and Supplemental Figure 5B). As expected, OMA1 levels were negatively correlated with OPA1 processing as a result of OMA1 autocatalysis upon activation (Figure 5A) (6). We also observed OMA1 cleavage of L-OPA1 in skeletal muscle tissue of the proband, demonstrating that the OMA1 stress response to C10 G58R protein was conserved between mice and humans (Figure 5D). These results show that the C10 G58R mutation caused widespread OMA1 activation in vivo.

### C10 G58R causes bioenergetic instability.

To further explore the mechanism of OMA1 activation by the C10 G58R mutation, we assessed C10 G58R expression in cultured cells. We first examined the localization of exogenously expressed C10 G58R in HeLa cells. By light microscopy, the majority of C10 G58R localized to mitochondria, consistent with prior reports (15, 23, 25), and immuno-electron microscopy revealed localization of C10 G58R to the IMS of the mitochondrial cristae (Supplemental Figure 6, A and B). Consistently, C10 G58R was predominantly in the mitochondrial fraction following subcellular fractionation and was protected from protease K in a pattern similar to that of endogenous WT C10 (Supplemental Figure 6, C and D). The IMS localization was important to C10 G58R toxicity, as the C10 G58R C132S double mutant, which cannot be oxidatively trapped in the IMS, caused less mitochondrial fragmentation than did the C10 G58R single mutant (Supplemental Figure 6, E and F).

**Figure 6:**
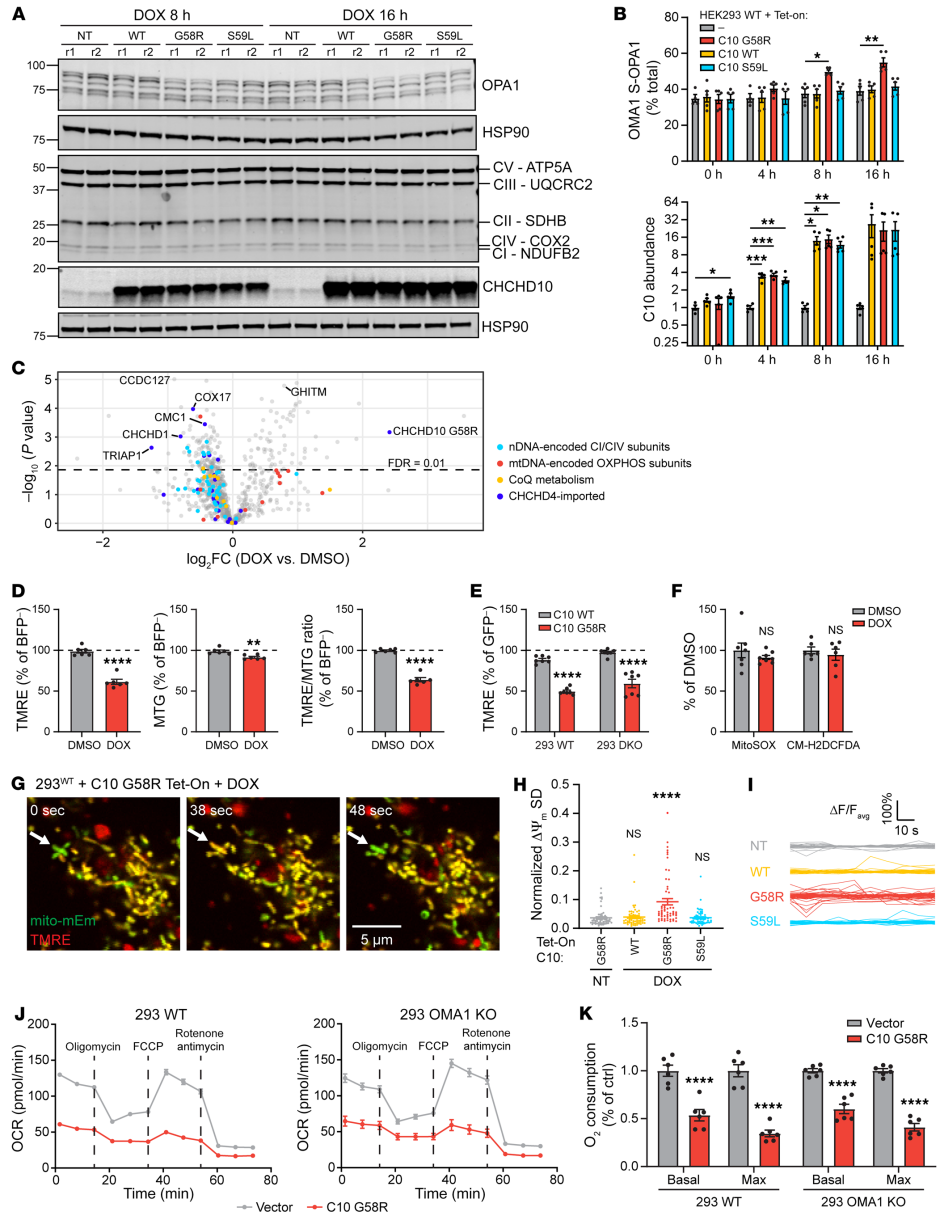
C10 G58R activates OMA1 in mouse and patient tissues and localizes to mitochondria. (**A**) Western blot showing WT and C10 WT/G58R/S59L dox-inducible HEK293 cell lines at the 8- and 16-hour dox (1 μg/mL) treatment time points. r1, replicate 1. (**B**) Quantification of OMA1 S-OPA1 (top) and C10 (bottom) levels from **A** and Supplemental Figure 7C (*n =* 4–5 biological replicates). (**C**) Protein differential abundance between HEK293 WT C10 G58R dox-inducible cells treated overnight with DMSO or dox (*n =* 4 biological replicates). nDNA, nuclear DNA. (**D**) Tetramethylrhodamine ethyl ester (TMRE) and MitoTracker Green (MTG) signal intensities in DMSO- and dox-treated HEK293 WT C10 G58R dox-inducible cells treated for over 24 hours (*n =* 6 biological replicates total on at least 2 occasions). (**E**) TMRE intensities in HEK293 WT (293 WT) and C2/C10-DKO cells transduced with WT C10 or C10 G58R (*n =* 7 biological replicates total on at least 2 occasions). (**F**) MitoSOX and CM-H_2_DCFDA fluorescence intensities in HEK293 WT C10 G58R dox-inducible cells treated overnight with DMSO or dox (*n =* at least 7 biological replicates on at least 2 occasions). (**G**) Representative confocal time series images of Tet-On HEK293 cells overexpressing C10 G58R. Arrows indicate a mitochondrial flicker event. Scale bar: 5 μm. mitomEm, mito-mEmerald. (**H**) Normalized SD of ΔΨ_m_ in mitochondria followed for 85 seconds (*n =* 60 mitochondria analyzed from 3 biological replicates). (**I**) Fluctuations in normalized ΔΨ_m_ of individual mitochondria followed for 85 seconds (*n =* 60 mitochondria analyzed from 3 biological replicates; each line represents an individual mitochondrion). ΔF/F_avg_, change in fluorescence intensity divided by the average fluorescence intensity. (**J**) Representative oxygen consumption rate (OCR) plots from Seahorse assays using HEK293 WT and OMA1-KO cells transduced with an empty vector or C10 G58R. (**K**) Quantification of basal and maximum oxygen consumption from the experiments in **J** (*n =* 6 biological replicates). Ctrl, control. Error bars represent the SEM. *P < 0.05, **P < 0.01, ***P < 0.001, and ****P < 0.0001, by 1-way ANOVA with Dunnett’s T3 multiple comparisons (**B** and **H**), permutation-based FDR with s_0_ = 0 (**C**), *t* test with Welch’s correction (**D** and **F**) and 2-way ANOVA with Sidak’s multiple comparisons (**E** and **K**). See also Supplemental Figures 7 and 8 and Supplemental data set 2.

We next assessed the effect of C10 G58R expression on OMA1 activation in cultured cells. As C10 RNA expression is approximately 10 to 100 times higher in human striated muscle compared with expression in commonly used human cell lines (Supplemental Figure 7A, data from Uhlén et al.; ref. 33), and endogenous levels of C10 G58R in neonatal fibroblasts from C10 G58R KI mice were not sufficient to activate OMA1 (Supplemental Figure 7B), we used a doxycycline-inducible (dox-inducible) system that could achieve a high level of C10 G58R expression in a temporally controlled manner. C10 G58R activated OMA1 approximately 8 hours after dox induction (Figure 6, A and B, and Supplemental Figure 7C). By contrast, C10 WT and C10 S59L at similar levels of expression did not activate OMA1 during the 16-hour time course (Figure 6, A and B, and Supplemental Figure 7C). Quantification showed small but significant decreases in complex I (CI), CIII, and CIV subunits with expression of C10 G58R for 16 hours, correlating with OMA1 activation (Figure 6A and Supplemental Figure 7, C and D). Notably, the decrease in oxidative phosphorylation (OXPHOS) subunits was not caused by OMA1 activation, as it was also observed following constitutive C10 G58R expression for more than 4 days in HEK293 OMA1-KO cells (Supplemental Figure 8, A–C). Consistent with the immunoblot results, most subunits belonging to CI and CIV were decreased following 24 hours of dox induction when measured by liquid chromatography tandem mass spectrometry–based (LC-MS/MS–based) proteomics (Figure 6C and Supplemental data set 2).

OMA1 is known to be activated under conditions of bioenergetic stress, including sustained loss of ΔΨ_m_ as well as instability of ΔΨ_m_ (also called mitochondrial flicker) (34, 35). Overexpression of C10 G58R decreased ΔΨ_m_ at the whole-cell level in both the presence and absence of endogenous C2 and C10 as measured by flow cytometry, and additionally increased OMA1 activation in the absence of endogenous C2 and C10 (Figure 6, D and E, and Supplemental Figure 8D). These effects are probably independent of ROS, as C10 G58R did not increase mitochondrial or cytoplasmic ROS, measured with the dyes MitoSOX and H_2_DCFDA, respectively (Figure 6F). Additionally, we found that C10 G58R overexpression (but not overexpression of C10 WT or C10 S59L) caused ΔΨ_m_ instability in individual mitochondria measured over time by live-cell imaging (Figure 6, G–I, and Supplemental Video 1). Consistent with its effect on ΔΨ_m_, C10 G58R expression decreased basal and maximal oxygen consumption in WT and OMA1-KO HEK293 cells (Figure 6, J and K). Taken together, these findings demonstrate that C10 G58R overexpression destabilized OXPHOS subunits and caused bioenergetic impairment either upstream of or parallel to the OMA1 stress response. We speculate that C10 G58R may activate OMA1 by impairing bioenergetics.

### OMA1 is critical for C10 G58R mouse neonatal survival.

**Figure 7:**
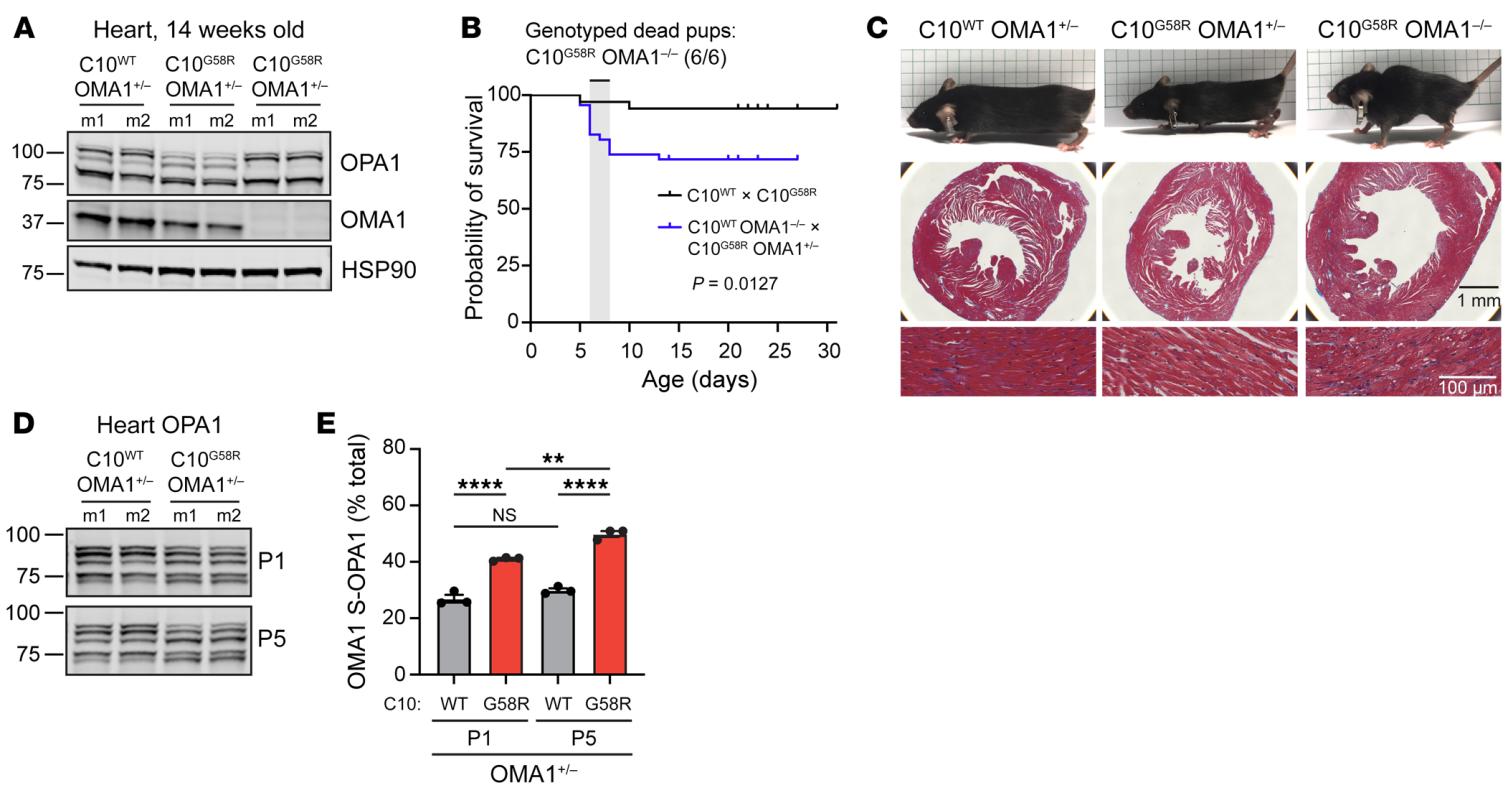
OMA1 is critical for C10 G58R pup survival. (**A**) Immunoblot of OPA1 and OMA1 levels in 14-week-old littermates of the C10^WT^ OMA1^–/–^ and C10^G58R^ OMA1^+/–^ cross. m, mouse (*n* = 4 mice per genotype). (**B**) Survival curves for pups from the C10^WT^ and C10^G58R^ cross and the C10^WT^ OMA1^–/–^ and C10^G58R^ OMA1^+/–^ cross. All 6 dead pups genotyped from the latter cross were C10^G58R^ OMA1^–/–^ (*n =* 18 pups from the C10^WT^ and C10^G58R^ cross; *n =* 46 pups from C10^WT^ OMA1^–/–^ and C10^G58R^ OMA1^+/–^ cross). (**C**) Top: 14-week-old littermates from the C10^WT^ OMA1^–/–^ and C10^G58R^ OMA1^+/–^ cross. Middle: Masson’s trichrome (MT) stainings of mouse hearts. Bottom: Magnified MT stainings of heart showing prominent vacuolation in C10^G58R^ OMA1^–/–^ hearts (*n* = 3 mice per genotype). Scale bars: 1 mm (middle) and 100 μm (bottom). (**D**) Immunoblot of OPA1 levels in hearts from C10^WT^ or C10^G58R^ mice on the OMA1^+/–^ background, on P1 and P5. Loading controls are shown in Supplemental Figure 9G (*n* = 3 mice per genotype). (**E**) Quantification of OMA-cleaved OPA1 bands (c+e/total) from immunoblot data in **D**. Error bars represent the SEM. **P < 0.01 and ****P < 0.0001, by log-rank test (**B**) and 2-way ANOVA with Sidak’s multiple comparisons (**E**). See also Supplemental Figure 9.

Having established that OMA1 is activated by the G58R mutation, we next asked whether the OMA1 stress response is protective or maladaptive. We crossed C10^G58R^ mice with OMA1-KO mice, which have a normal lifespan and tend to be heavier (Figure 7A) (36). To our surprise, the G58R mutation was neonatally lethal for mice on the OMA1-KO background, with few C10^G58R^ OMA1^–/–^ mice surviving to weaning (Figure 7B, Supplemental Figure 9A, and Table 2). Most of the C10^G58R^ OMA1^–/–^ pups died between P5 and P8, with the few survivors (escapees) dying by 15 weeks of age. Notably, the escapees had markedly enlarged hearts despite a decreased body size (Figure 7C). KO of OMA1 additionally worsened the mtDNA deletion load (Supplemental Figure 9B) and increased the number of p62, but not C10, aggregates in C10^G58R^ mouse hearts (Supplemental Figure 9C).

To further investigate the neonatal lethality of this cross, we examined timed pregnancies. C10^G58R^ OMA1^–/–^ mice were grossly indistinguishable from their littermates at E18.5 and P5 (Supplemental Figure 9, D and E) and did not differ in body weight at P1 (Supplemental Figure 9F). However, their body weight was significantly lower than that of their OMA1^+/–^ C10^G58R^ littermates at P5 (Supplemental Figure 9F), indicating decompensation. In the heart, OMA1 was already activated at P1 and OMA1 activity increased at P5 (Figure 7, D and E, and Supplemental Figure 9G). In marked contrast to the cross between C10^G58R^ OMA1^+/–^ and OMA1^–/–^ mice, the cross between C10^S59L^ OMA1^+/–^ and OMA1^–/–^ mice was not neonatally lethal (Supplemental Figure 9H). Together, these results demonstrate that the OMA1 stress response was critical for G58R survival, with decompensation starting around P5.

### OMA1 fragments heart mitochondria in response to C10 G58R stress.

**Figure 8:**
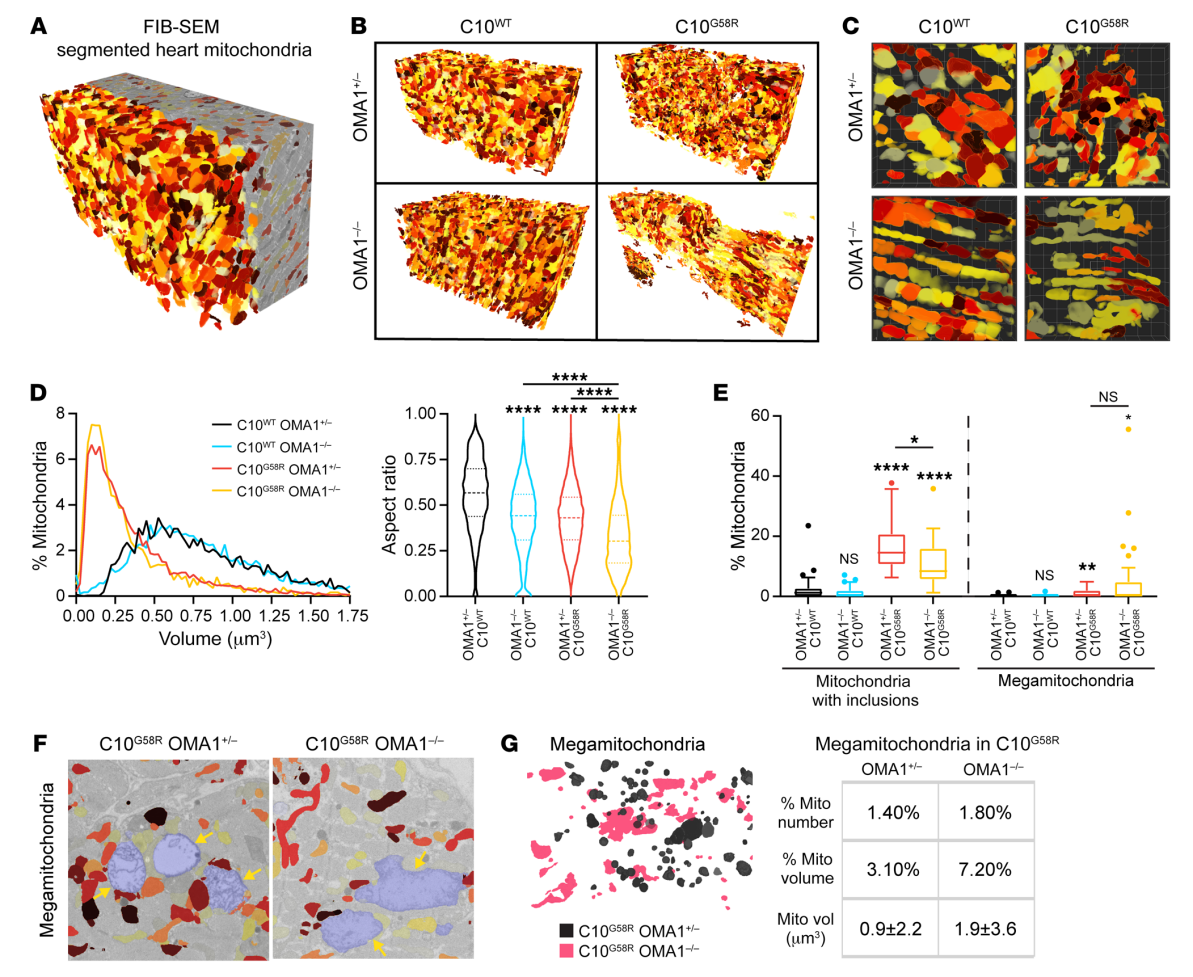
The C10 G58R mutation fragments heart mitochondria in an OMA1-dependent manner. (**A**) Reconstruction of segmented FIB-SEM mouse heart mitochondria. Colors are arbitrary and only applied to aid visualization. (**B**) Complete data sets of 3D-reconstructed heart mitochondria from 14-week-old mice of the indicated genotypes. (**C**) Magnification of representative fields from the data sets in **B**. (**D**) Volume distribution and aspect ratio measurements of mitochondria from **B** (*n* >3159 mitochondria for each genotype from 1 biological replicate). (**E**) Percentage of mitochondria with inclusions and percentage of megamitochondria from TEM images of 14-week-old mouse hearts (*n =* 3 mice per genotype for all genotypes except OMA1^+/–^ C10^G58R^, for which *n =* 2; *n* = 15 FOV quantified per mouse, with >1900 mitochondria assessed per genotype). (**F**) Megamitochondria (shaded in blue) from the FIB-SEM stack. Nonmegamitochondria are colored for comparison. (**G**) Left: Overlay of segmented megamitochondria from C10^G58R^ mice on the OMA1^+/–^ (black) or OMA1^–/–^ (pink) background. Right: Percentage of megamitochondria in the FIB-SEM stack, percentage of megamitochondrial volume of the total mitochondrial volume (mito vol), and average volume of individual megamitochondria (*n =* 119 OMA1^+/–^ C10^G58R^ megamitochondria; *n =* 58 OMA1^–/–^ C10^G58R^ megamitochondria). Error bars represent the SEM. For the violin plots in **D**, the 25th quartile, median, and 75th quartile are indicated. Box-and-whisker plot lines in **E** were calculated with Tukey’s method. *P < 0.05, **P < 0.01, and ****P < 0.0001, by 1-way ANOVA with Games-Howell’s multiple comparisons (**D**) and Dunnett’s T3 multiple comparisons (**E**). See also Supplemental Figure 11.

OMA1 cleavage of L-OPA1 inhibits mitochondrial fusion, causing mitochondrial fragmentation (2, 3, 7). We found that exogenously expressed C10 G58R fragmented mitochondria in HeLa cells by activating OMA1, as we observed previously (15) and, additionally, narrowed the mitochondrial caliber in OMA1-KO cells (Supplemental Figure 10). To assess the effects of G58R and OMA1 activation in vivo, we analyzed heart mitochondria in a surviving 14-week-old C10^G58R^ OMA1^–/–^ mouse and its littermates by focused ion beam scanning electron microscopy (FIB-SEM), followed by segmentation and 3D reconstruction of heart mitochondria (Figure 8A and Supplemental Video 2). C10^G58R^ OMA1^+/–^ mitochondria were smaller than C10^WT^ OMA1^+/–^ mitochondria (Figure 8, B–D), reflecting mitochondrial fragmentation. C10^WT^ OMA1^–/–^ mitochondria had the same average volume as C10^WT^ OMA1^+/–^, but they tended to be longer, as reflected by the decreased aspect ratio (Figure 8D). Interestingly, C10^G58R^ OMA1^–/–^ mitochondria were also longer but tended to be decreased in caliber and thus smaller in volume, similar to what we observed in cell culture with exogenous C10 G58R expression. These mitochondria also had the lowest aspect ratio, reflecting their long, thin morphology. These data demonstrate that OMA1 activation causes mitochondrial fragmentation in vivo.

Next, we analyzed heart tissue from these genotypes by transmission electron microscopy (TEM), which has superior lateral resolution. As expected, there were more inclusions in the C10^G58R^ mutants on either background compared with C10^WT^ mouse hearts (Figure 8E). Notably, OMA1 KO partially blocked inclusion formation, suggesting that OMA1 activity may facilitate (but is not required for) their formation (Figure 8E). Additionally, in C10^G58R^ mutants on both backgrounds, we observed megamitochondria: massively swollen mitochondria with lighter matrix density compared with surrounding mitochondria (Figure 8E and Supplemental Figure 11A), similar to what has been observed in other models of mitochondrial or metabolic stress (37). These megamitochondria were also seen in the FIB-SEM data sets and immunofluorescence-stained sections of C10^G58R^-mutant hearts (Figure 8F, Supplemental Figure 11B, and Supplemental Video 3). While the proportion of megamitochondria was similar for the 2 genotypes, the overall morphology of the megamitochondria (e.g., long or round) reflected that of the nonmegamitochondria from mice of their respective genotypes (Figure 8G).

As mitochondrial fission and megamitochondria can be associated with oxidative damage and mitophagy (38, 39), we also tested for signs of oxidative damage and autophagy. The autophagy adaptor p62 was increased in the heart by immunoblotting (Supplemental Figure 11, C and E), consistent with the appearance of p62 punctae by immunofluorescence (Supplemental Figure 9C) and similar to what has been described for the C10^S59L^ mouse (11). Additionally, we found that the LC3-II/LC3-I ratio was not significantly changed, suggesting that increased p62 may reflect binding to misfolded protein (Supplemental Figure 11, C and E). Finally, 4-HNE immunoblotting showed no increase in oxidatively damaged protein (Supplemental Figure 11, D and E). Thus, we did not observe signs of substantial oxidative damage or mitophagy in C10 G58R hearts.

Taken together, these findings demonstrate that OMA1 fragmented mitochondria in response to the G58R mutation in vivo and permitted reshaping of the inner membrane to form inclusions.

### OMA1 signals mitochondrial stress through the ISR.

**Figure 9:**
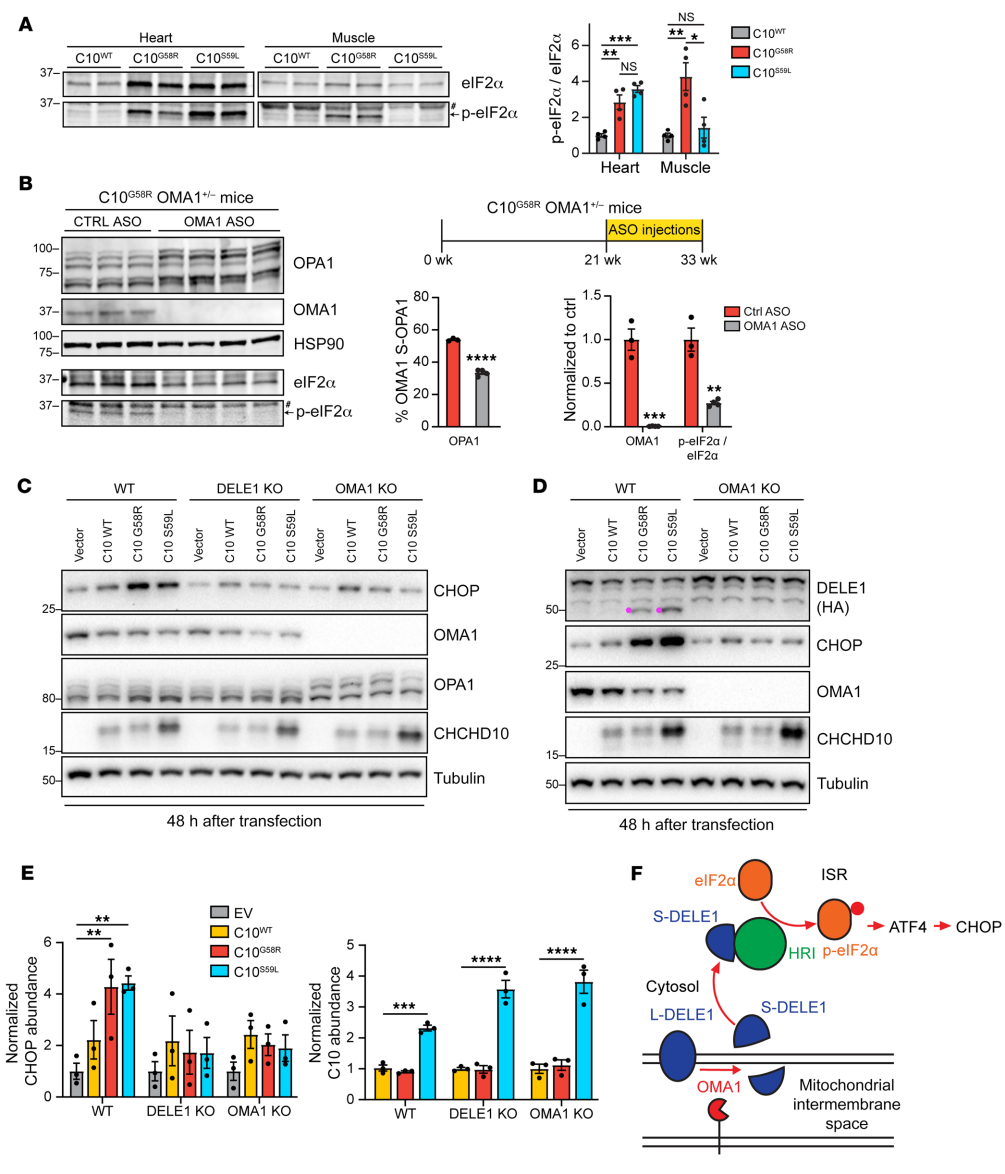
OMA1 signals C10 G58R mitochondrial stress to activate the integrated stress response through DELE1. (**A**) Representative immunoblot of eIF2α and p-eIF2α levels in heart and muscle lysates from 37- to 44-week-old C10^WT^, C10^G58R^, and C10^S59L^ mice. ^#^Nonspecific band. Graph shows quantification of immunoblot results from **A** (*n =* 4 mice per genotype). (**B**) Timeline of the ASO experiment and immunoblots for OPA1, OMA1, eIF2α, and p-eIF2α levels in C10^G58R^ mouse hearts on the OMA1^+/–^ background, treated with either a nontargeting (ctrl) or OMA1 ASO. Loading controls for eIF2α and p-eIF2α are shown in Supplemental Figure 12F. ^#^Nonspecific band. Quantification is shown in the graph (*n =* 3 control ASO–treated mice; *n =* 4 OMA1 ASO–treated mice). (**C**) Effect of 48-hour C10 WT, C10 G58R, and C10 S59L overexpression on CHOP levels and OPA1 processing in HEK293T cells on the WT, DELE1-KO, or OMA1-KO backgrounds. (**D**) Effect of 48-hour C10 WT, C10 G58R, and C10 S59L overexpression on DELE1 processing into S-DELE1 (pink) in HEK293T WT and OMA1-KO cells endogenously expressing DELE1-HA. (**E**) Quantification of CHOP and C10 levels from the immunoblot results in **C** (*n =* 3 biological replicates). (**F**) Model of OMA1 activating the integrated stress response through DELE1. Error bars represent the SEM. *P < 0.05, **P < 0.01, ***P < 0.001, and ****P < 0.0001, by 1-way ANOVA with Dunnett’s T3 multiple comparisons test (**A** and **E**) and *t* test with Welch’s correction (**B**). See also Supplemental Figure 12.

A stress response involving ATF4 has previously been observed in both C10^S59L^ and C2/C10-DKO mice (as well as in other mitochondrial myopathy models), although the mechanism for this response in vivo has not been elucidated (11, 15). ATF4 is often activated downstream of eIF2α phosphorylation in what is known as the ISR. To see if the ISR was activated in our models, we immunoblotted for eIF2α phosphorylation. The ratio of phosphorylated eIF2α (p-eIF2α) to unphosphorylated eIF2α was significantly increased in C10^G58R^ and C10^S59L^ hearts and C10^G58R^ muscle compared with levels in C10^WT^ muscle, demonstrating ISR activation (Figure 9A).

We next asked whether OMA1 mediates the ISR in vivo, similar to what was recently reported in cultured cells (8, 9). In addition to analyzing the surviving C10^G58R^ OMA1^–/–^ mice, which were decompensated as discussed above, we knocked down OMA1 in adult C10^G58R^ OMA1^+/–^ mice using either nontargeting or one of 2 OMA1-specific antisense oligomers (ASOs) (Figure 9B). In contrast to the constitutive OMA1 KO, knockdown (KD) of OMA1 in adult animals was relatively well tolerated over the 12-week treatment period, with only 2 of 9 OMA1 ASO-treated animals dying, and no or mild effects on cardiac function, motor function, and mtDNA stability (Supplemental Figure 12, A–E, and Supplemental data set 1). Immunoblotting confirmed KD of OMA1 and partial restoration of the long (noncleaved) forms of OPA1 in ASO-treated mouse hearts (Figure 9B). Since the 2 OMA1 ASOs performed similarly, they were grouped together for analysis. The ratio of p-eIF2α to eIF2α was decreased to 27% of control ASO following OMA1 ASO treatment (Figure 9B and Supplemental Figure 12F), demonstrating inhibition of the ISR.

To further evaluate whether C10 G58R and S59L induce the ISR through the recently described OMA1/DELE1 pathway (8, 9), we assessed ISR activation in 293T cell lines lacking either OMA1 or DELE1. Expression of C10 G58R or C10 S59L, but not WT C10, significantly induced expression of the ISR-dependent gene CHOP, 32 and 48 hours after transfection (Figure 9, C and E, and Supplemental Figure 12, G–L). ISR induction (manifested through increased CHOP levels) depended on both OMA1 and DELE1 (Figure 9, C and E, and Supplemental Figure 12, G–L). OMA1 activates the ISR by cleaving the full-length DELE1 (L-DELE1) to a short form (S-DELE1), which then activates heme-regulated inhibitor (HRI) in the cytosol (8, 9). Consistently, expression of both C10 G58R and C10 S59L generated S-DELE1 in an OMA1-dependent manner (Figure 9D and Supplemental Figure 12, G and J). Taken together, these findings demonstrate that C10 G58R and S59L activate the ISR through the OMA1/DELE1 pathway (Figure 9F).

**Figure 10:**
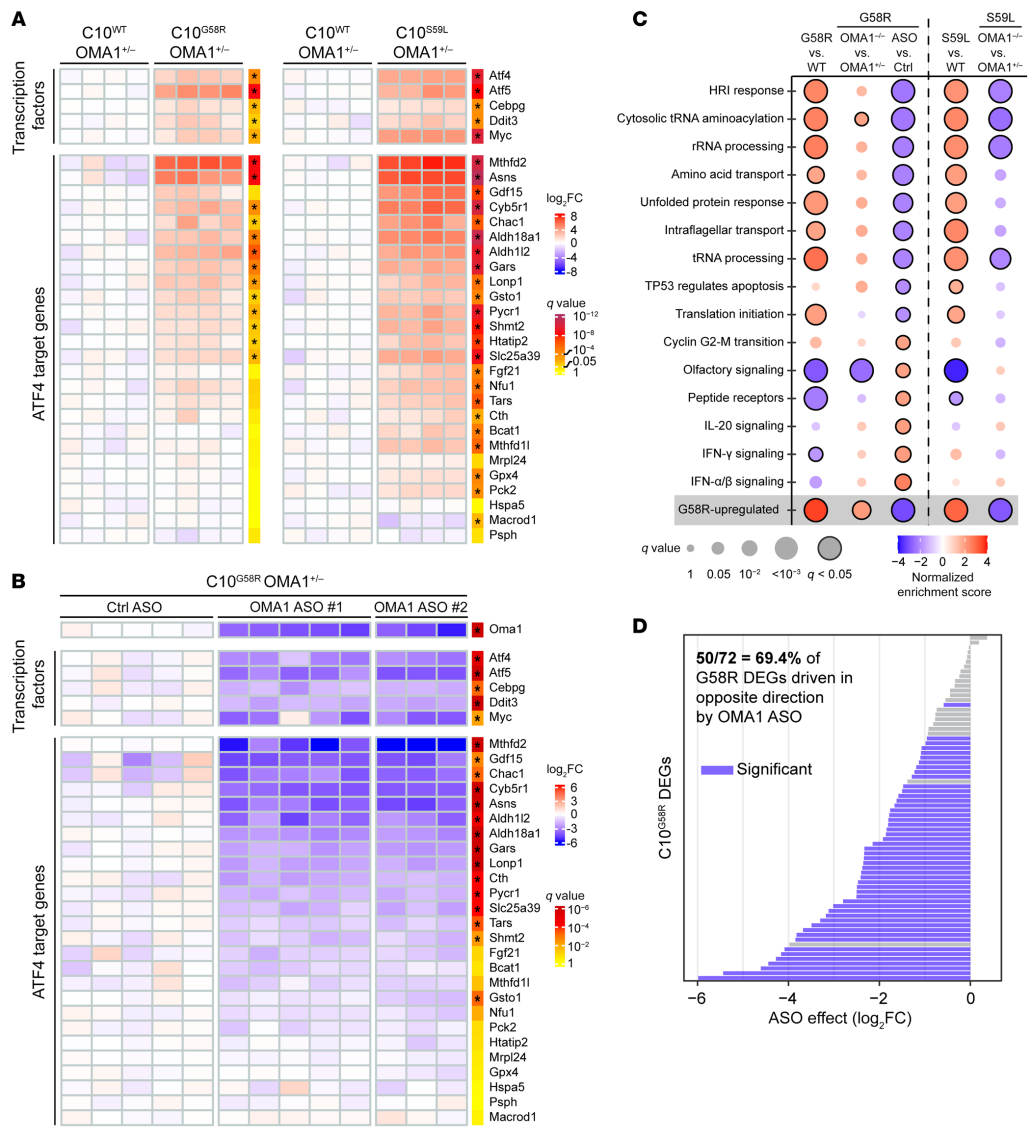
Transcriptomic analyses of the ISR in C10-mutant mice. (**A**) Microarray data on hearts from 14-week-old C10^G58R^ versus C10^WT^ mice and 20-week-old C10^S59L^ versus C10^WT^ mice, all on the OMA1^+/–^ background. Each column represents a mouse, and *q* values represent genome-wide significance. *q < 0.05. (**B**) Microarray data for 33-week-old C10^G58R^ mouse hearts on the OMA1^+/–^ background treated with either a nontargeting control ASO or an OMA1 ASO. Each column represents a mouse, and *q* values represent genome-wide significance. *q < 0.01. (**C**) GSEA of reactome pathways for the listed comparisons. Pathways shown are all the pathways with a *q* value of less than 0.025 in the C10^G58R^ OMA1 ASO versus control ASO comparison. (**D**) Effect of OMA1 ASO on the expression of G58R DEGs identified in the comparison from **A**. See also Supplemental Figure 13 and Supplemental data sets 3 and 4.

To examine the ISR further, we looked at global gene expression in C10^G58R^ and C10^S59L^ mouse hearts on an OMA1^+/–^ background (Supplemental data set 3). Gene set enrichment analysis (GSEA) of reactome pathways showed that EIF2AK1 (HRI) response, tRNA aminoacylation, and mitochondrial translation were among the most upregulated pathways in C10^G58R^ versus C10^WT^ hearts (Supplemental data set 4). Indeed, we found that transcription factors involved in the ISR (ATF4, ATF5, CHOP, and CEBPG) were all significantly upregulated in both C10^G58R^ and C10^S59L^ mouse hearts (Figure 10A), along with several ATF4 targets, including MTHFD2, GDF15, FGF21, ASNS, LONP1, and ALDH18A1. Many of these same ISR-associated genes were downregulated by OMA1 ASO treatment compared with control ASO treatment in C10 G58R-KI mice (Figure 10, B and C, and Supplemental data set 4), demonstrating that the OMA1 stress response is responsible for the transcriptional upregulation of these genes. Altogether, approximately 70% of the differentially expressed genes (DEGs) identified in the C10^G58R^ versus C10^WT^ comparison were significantly suppressed by OMA1 ASO treatment, suggesting that OMA1 drove most of the transcriptional response to the G58R mutation (Figure 10D). Similar to OMA1 KD in the C10^G58R^ mice, constitutive KO of OMA1 in C10^S59L^ mice suppressed the ISR (Supplemental Figure 13). By contrast, constitutive KO of OMA1 in C10^G58R^ mice mildly increased the expression of some ATF4-dependent genes, likely reflecting alternative activation of ATF4 in the decompensated mice (Supplemental Figure 13). Taken together, these data demonstrate that OMA1 signals IMM stress through the ISR in vivo.

### OMA1 activation shapes the mitoproteome through mitonuclear signaling.

**Figure 11:**
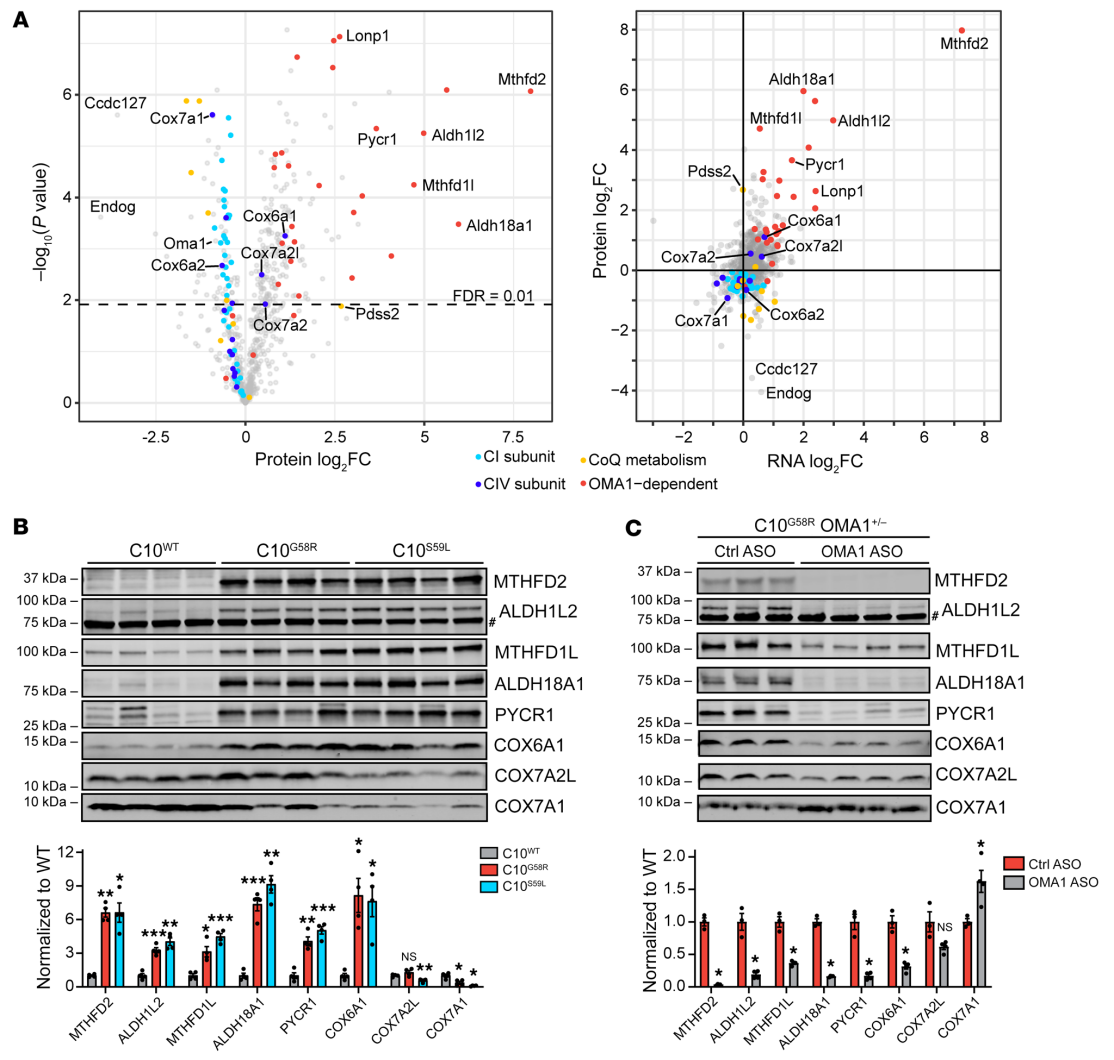
OMA1 activation shapes the mitoproteome through mitonuclear signaling. (**A**) Left: Volcano plot of protein fold change (FC) in 36-week-old C10^G58R^ versus C10^WT^ mouse heart mitochondria quantified by label-free mass spectrometry (*n =* 4 mice per group). Right: Protein fold change versus transcript fold change in C10^G58R^ versus C10^WT^ mouse hearts. The proteomics data are from 36-week-old C10^G58R^ versus C10^WT^ mice on the WT background, and the transcriptomics data are from 14-week-old C10^G58R^ versus C10^WT^ littermates on the OMA1^+/–^ background. (**B**) Validation of some proteins that were significantly upregulated or downregulated in the C10^G58R^ versus C10^WT^ proteomics data set. Immunoblot of C10^WT^, C10^G58R^, and C10^S59L^ mouse heart lysates. Loading controls are shown in Supplemental Figure 14A. ^#^Nonspecific band. (**C**) Immunoblot of heart lysates from 33-week-old C10^G58R^ mice on the OMA1^+/–^ background treated with a nontargeting control ASO or an OMA1 ASO. Loading controls are shown in Supplemental Figure 14B. ^#^Nonspecific band. Error bars represent the SEM. *P < 0.05, **P < 0.01, and ***P < 0.001, by permutation-based FDR with s_0_ = 0 (**A**), 1-way ANOVA with Dunnett’s T3 multiple comparisons (**B**), and multiple *t* tests with Welch’s correction and correction for multiple comparisons with the 2-stage step-up (Benjamini, Krieger, and Yekutieli) method (**C**). See also Supplemental Figure 14 and Supplemental data set 2.

We next examined how the G58R mutation affects the heart mitochondrial proteome by label-free quantification mass spectrometry. Endonuclease G (Endog) and Ccdc127 were the 2 proteins with the greatest decrease in C10^G58R^ versus C10^WT^ heart mitochondria. The levels of OMA1 were also decreased, as expected, given its autocatalysis when activated (Figure 11A, left). Enzymes involved in coenzyme Q metabolism were also found to be overall decreased, with the exception of the upstream enzyme Pdss2, similar to what has been previously observed in models of decreased mtDNA expression (14). CI and CIV subunits were mildly decreased overall in C10^G58R^ heart mitochondria, consistent with the decrease in CI and CIV activity.

In contrast to the other CIV subunits, 3 were increased: Cox6a1, Cox7a2, and Cox7a2l. These CIV isoforms (called the “liver” isoforms) are not typically expressed in adult striated muscle but are the dominant isoforms in most other tissues (40, 41). The “heart” isoforms Cox6a2 and Cox7a1 were decreased, suggesting that CIV may undergo a subunit switch favoring liver over heart subunits (Figure 11, A and B, and Supplemental Figure 14, A and C). Notably, this switch occurred in each of the intact CIV complexes, separated by blue native PAGE (BN-PAGE) gel electrophoresis (Supplemental Figures 14, E and F, and Supplemental data set 2). Overall, the monomeric form of CIV was relatively preserved in C10^G58R^ hearts, but CIV-containing supercomplexes were decreased (Supplemental Figure 14C, right).

Plotting protein versus RNA expression showed that almost all OMA1-regulated genes were concordantly increased, suggesting that these protein changes are likely transcriptionally driven (Figure 11A, right). Among the most increased OMA1-dependent genes were enzymes in the mitochondrial 1-carbon (1C) metabolism and proline synthesis pathways, which are known targets of the ISR. Notably, the liver CIV isoforms were also increased, whereas the heart isoforms Cox6a2 and Cox7a1 were decreased, suggesting that the CIV isoform switch may also be mediated by the ISR.

We next assessed whether these changes to the mitochondrial proteome depended on OMA1. Enzymes in the 1C metabolism and proline synthesis pathways were significantly increased in C10^G58R^ and C10^S59L^ hearts and were suppressed by KD of OMA1 in C10^G58R^ hearts (Figure 11, B and C, and Supplemental Figure 14, A and B), confirming that their upregulation at the protein level was signaled by OMA1. Similarly, the liver isoform Cox6a1 increased in C10^G58R^ and C10^S59L^ hearts and was suppressed in C10^G58R^ hearts by OMA1 ASO treatment, confirming that this isoform was also upregulated by OMA1. By contrast, the heart isoform Cox7a1 exhibited a converse pattern; it was decreased in both C10^G58R^ and C10^S59L^ hearts and increased in C10^G58R^ hearts treated with OMA1 ASO, suggesting that it may be negatively regulated by OMA1. Considering all identified high-confidence, OMA1-regulated mitochondrial genes, the majority were significantly upregulated at both the transcript and protein levels and suppressed by OMA1 ASO at the transcript level (Supplemental Figure 14D). These results demonstrate that an OMA1-dependent transcriptional response rewired the heart mitochondrial proteome in response to the C10 G58R mutation.

## Discussion

Through genetic characterization of a C10 G58R family and a C10^G58R^-KI mouse model, we have established that the G58R mutation is pathogenic, causing aggregate formation and autosomal dominant IMMD. OMA1 is activated in response to C10 G58R misfolding to mediate a protective response that involves mitochondrial fragmentation locally and signaling outside the mitochondria to activate the ISR. The ISR reshapes the mitochondrial proteome, including upregulation of the “liver” isoforms of CIV subunits, which we identify as a component of the ISR in striated muscle. To our knowledge, this is the first demonstration that the OMA1 stress response can be critical for neonatal survival and can mediate mitonuclear signaling through the ISR in vivo.

The heterozygous C10 G58R variant was first identified in *cis* with R15S in a large US family with autosomal dominant myopathy (25, 26). Here, we presented a family from the UK exhibiting myopathy and cardiomyopathy with a de novo G58R mutation in isolation, establishing that the G58R variant is pathogenic. The study of the US and UK families share several features, including (a) a generalized myopathy presenting in the first decade of life; (b) myopathic features and an absence of fibrillations on needle electromyography; (c) moderately decreased complex II-III activity and severely decreased complex IV activity; and (d) intramitochondrial inclusions that we found to be dilations of the cristae with intracristal membranes.

Despite their similarities, the disease severity differed between these US and UK families. In the US family, some members lived into their 60s, facial weakness was delayed until the third or fourth decade, and cardiomyopathy was not a general feature (26). By contrast, the 3 affected members of the UK family all died before the age of 35, and all had early facial weakness and cardiomyopathy. Although the reason for this difference is not clear at present, the genetic background including a potential protective effect of the *cis* R15S variant in the US family may play a role.

The C10 G58R and S59L mutations — while affecting neighboring residues — cause different phenotypes, with a more severe myopathy in patients with G58R mutations and upper and lower motor neuron involvement (as well as cerebellar, cortical, and possible nigrostriatal involvement) in patients with S59L mutations (27). As in humans, we found myopathy to be more severe in the C10^G58R^ mice than in the C10^S59L^ mice. The differential toxicities of G58R and S59L in muscle and nerve may relate to the distinct toxic conformations they adopt. Notably, C10 G58R and S59L exhibited differential solubility and formed aggregates with distinct morphologies and localizations in the affected heart tissue. Thus, although misfolding into toxic conformations may be a shared pathogenetic mechanism for dominant C10 and C2 mutations, distinct toxic conformations may cause distinct phenotypes. This parallels recent observations of other toxic misfolding proteins, such as α-synuclein and tau, which misfold into different pathogenic strains, each of which causes a distinct but related neurodegenerative disorder (42, 43).

C10 G58R dramatically distorted the IMM, with mitochondrial cristae forming focal dilations with internal vesicles. The IMM is commonly altered in models with mitochondrial dysfunction, and rod-like intracristal inclusions are known to develop in response to creatine depletion (44). However, the morphology of C10 G58R inclusions was distinct and plausibly reflected strain on the IMM from C10 G58R misfolding. Notably, the disruption of the IMM was more pronounced in the C10^G58R^ model than the comparatively mild effects on OXPHOS subunit expression, suggesting that IMM disruption may have been a proximal event. We propose that the morphogenesis of these intracristal inclusions may involve dilation of the cristae followed by internalization of membranes (possibly by inward budding), as some dilated cristae were empty, but most cristae with internal vesicles were dilated. Interestingly, L-OPA1, which is thought to stabilize the neck of cristae (45), may partially inhibit the formation of these inclusions, as we found that fewer inclusions formed in the absence of OMA1. However, although their formation may be modulated by OMA1, they seemed to be driven by C10 G58R toxicity, as they were observed in the presence or absence of OMA1. It is not clear at present whether C10 G58R disrupts the IMM through a direct interaction with the membrane or through an indirect mechanism.

Strikingly, OMA1 activation was critical for neonatal survival of C10^G58R^ mice, with only a few escapees surviving to young adulthood. Escape from neonatal lethality is also seen in other mitochondrial disorders, such as reversible infantile respiratory chain deficiency, and may reflect metabolic rescue by an alternatively activated ISR and/or the mTOR pathway (46). The neonatal lethality we observed in the C10^G58R^ OMA1^–/–^ double mutants contrasts with previously reported models, including YME1L conditional heart KO and PHB2 conditional brain KO models, in which OMA1 activation drove the pathology (18, 19). The trigger for OMA1 activation, however, may be different in these models; YME1L, OMA1, and PHB2 associate within inner membrane proteolytic hubs, the disruption of which may lead to aberrant OMA1 activation (47, 48). Activation of OMA1 by C10 G58R, by contrast, may be through bioenergetic instability. Notably, C10 G58R caused lower ΔΨ_m_ and unstable ΔΨ_m_ when expressed in cultured cells. These perturbations can be directly sensed by a voltage sensor in OMA1 to increase its activity (6, 34, 35). However, direct evidence for a causal link between lowered ΔΨ_m_ from C10 G58R expression and OMA1 activation is still lacking, and another mechanism of OMA1 activation by C10 G58R is possible.

In addition to demonstrating that OMA1 mediated mitochondrial fragmentation in response to C10 G58R stress, we showed in vivo that OMA1 signaled mitochondrial stress to the nucleus through the ISR. This involves the OMA1-mediated cleavage of DELE1 in the IMS, as part of the OMA1/DELE1/HRI pathway recently described in cell culture studies (8, 9). This mechanism is likely shared by other mitochondrial stresses that disrupt inner membrane integrity or otherwise cause instability of ΔΨ_m_ to activate OMA1.

Although we showed that OMA1 was necessary for the survival of C10^G58R^ pups, it remains unknown which function of OMA1 is necessary: mitochondrial fragmentation (likely through L-OPA1 cleavage), activation of the ISR (through DELE1/HRI), and/or an uncharacterized function. Future studies focused on the 2 best established substrates of OMA1, DELE1 and OPA1, may help clarify the importance of each to the OMA1 stress response.

Our findings beg the question: Do all forms of mitochondrial dysfunction activate the ISR through OMA1? Notably, the ISR is potently activated by mutations or toxins that decrease mtDNA expression (sometimes termed mitonuclear imbalance) (14, 17). Are these stress responses also OMA1 dependent? Two lines of evidence suggest that they may not be. Although cell culture studies found that diverse mitochondrial toxins activated the ISR through OMA1 and DELE1, inhibition of mtRNA translation with dox did not (9). Additionally, OMA1-independent modes of DELE1 signaling have been detected (10). Using OMA1 levels as a biomarker of OMA1 activation, we compared the heart mitoproteome of our C10^G58R^ model with models with reduced mtDNA expression (14). Although the ISR was activated to a similar extent in all models, OMA1 levels were reduced as expected in the C10^G58R^ model but were unchanged or increased following diminished mtDNA expression (Supplemental Figure 15A). Notably, models with diminished mtDNA expression had larger reductions in OXPHOS subunits than we observed in our C10^G58R^ model (Supplemental Figure 15A). We speculate that there are at least 2 mitochondrial ISR pathways: one that signals loss of IMM integrity through OMA1, and another that signals severe OXPHOS deficiency independently of OMA1. Plausible mechanisms for the OMA1-independent ISR activation include GCN2 activation in response to asparagine deficiency or mTOR activation, as proposed previously (13, 16, 49).

In addition to well-characterized ISR-dependent changes in the heart, we observed upregulation of the ubiquitous liver subunits of CIV (including Cox6a1, Cox7a2, and Cox7a2l) and downregulation of their heart counterparts (Cox6a2 and Cox7a1). Analysis of proteomics data from a previous study of 5 models with decreased mtDNA expression showed a similar shift between the liver and heart isoforms, suggesting that this is a general feature of the ISR in striated muscle (Supplemental Figure 15B) (14). Although the liver isoforms are present in striated muscle at birth in mice, their expression decreases at around 4 weeks of age, as these isoforms are replaced by their heart counterparts (50). Although Cox7a2l has been observed previously to increase in response to an ER stress–mediated ISR (through PERK) (51), our findings place this change in the broader context of a subunit switch in CIV favoring the more generally expressed liver subunits.

In summary, we have shown that the C10 G58R mutation causes a pathologically distinct form of mitochondrial myopathy, IMMD. In response to C10 G58R misfolding, OMA1 mediates a protective response that fragments mitochondria locally and signals mitochondrial stress to the nucleus through the ISR. As the OMA1 stress response is protective, interventions facilitating it may have therapeutic potential. Additionally, as C10 G58R has a toxic gain-of-function mechanism and C10 KO is tolerated (15, 22), reducing C10 expression may be a viable therapeutic strategy for IMMD. Finally, it is notable that FGF21 was elevated in the proband’s serum and that expression of FGF21 and GDF15 was dependent on the OMA1 stress response in the C10 G58R mouse model. As both FGF21 and GDF15 are serum proteins that are commonly elevated in mitochondrial myopathies, they may serve as biomarkers of target engagement and treatment responses in IMMD therapeutic trials (52, 53).

## Methods

Further information can be found in the Supplemental Methods (54–66).

### Data availability.

The microarray data generated in this study have been deposited in the NCBI’s Gene Expression Omnibus (GEO) database (GEO GSE189393 and GSE189396) and the analyzed data are provided in Supplemental data set 3. Proteomics data are provided in Supplemental data set 2.

### Genetic and clinical characterization of the CHCHD10 p.G58R family.

The proband of the family was referred to the Mitochondrial Diagnostic Centre at Oxford University for clinical assessment as well as histological, biochemical, and molecular genetic analyses.

### Mouse models.

Mice were maintained on a 12-hour light/12-hour dark cycle, with food and water provided ad libitum. The C10^G58R^ mouse was generated using CRISPR/Cas9 endonuclease-mediated genome editing on a C57Bl6J background. The generation of C10^S59L^, C2/C10-DKO, and OMA1-KO mice was described previously (15, 36).

### Motor function tests.

C10^WT^ and C10^G58R^ littermate mice, aged 18 weeks, and C10^S59L^ mice, aged 25 weeks, were tested. Mouse forelimb grip strength was measured by pulling the mouse and recording the force generated as the mouse gripped onto the instrument (BIOSEB instrument with bar, catalog EB1-BIO-GS3). Balance and motor coordination were tested by placing the mice on a rotarod (Ugo Basile, catalog 57624) and measuring the time to fall. Endurance and fatigue were measured with the treadmill fatigue test adapted from a previously published protocol (67).

### Mouse CI and CIV activity assays.

Assays were performed according to the kit instructions (Abcam; CI rodent: AB109721, CIV rodent: AB109911). All preparation steps, including homogenization, were performed on ice, and 30–60 mg samples of liquid-nitrogen flash-frozen mouse heart or muscle were used.

### Human histochemistry and complex activities.

Histochemistry and ETC complex activities were performed according to standard methods within the context of an accredited National Health Service (NHS) diagnostic laboratory (68–70).

### Cell transfection.

HeLa cells, HEK293 cells, or 293T cells were transfected with WT or mutant C10 constructs using FuGENE 6 transfection reagent (Promega, catalog PRE2691) in all instances except for the experiments shown in Figure 9 and Supplemental Figure 12, in which PEI 25K (Polysciences, catalog 23966) was used. For the experiments involving Su9-EGFP, the cells were transfected with both C10 constructs and Su9-EGFP at a 3:1 DNA ratio. The cells were incubated at 37°C at least overnight before downstream analysis.

### Immunoblotting.

Immunoblotting and densitometric measurements were performed as described previously (15).

### Mitochondrial isolation.

Mitochondria were isolated from cells or whole mouse hearts as described previously (71).

### Super-resolution imaging.

The super-resolution images shown in Figure 2D, Figure 3B, Supplemental Figure 1F, Supplemental Figure 4E, Supplemental Figure 6, A and F, Supplemental Figure 10A, and Supplemental Figure 11B were acquired using an LSM 880 with Airyscan microscope (Zeiss) as *z*-stacks, with a minimum of 5 images. The images were 3D deconvolved using the default settings in the Zeiss Zen software. The images shown are maximum-intensity projections of the *z*-stack produced using ImageJ/Fiji (NIH).

### Cell lines.

HeLa^OMA1 KO^ cells were a gift from Richard Youle (NIH), and their generation was described previously (72). HEK293^C2/C10 DKO^, HEK293^OMA1 KO^, and HEK293 C10 G58R Tet-inducible cells were described previously (15), and HEK293 C10 WT and S59L Tet-inducible cells were generated in the same manner, using the sleeping beauty transposon system. These cells all also express blue fluorescent protein (BFP) from a separate promoter. Where indicated, HEK293^WT^, HEK293^OMA1 KO^, and HEK293^C2/C10 DKO^ cell lines were transduced with empty vector, C10 WT, C10 G58R, or C10 S59L subcloned into the pCIG3-IRES-GFP vector as described previously (15). Experiments with the transduced cells were performed 4–5 days after transduction. The original HEK293 and HeLa cell lines were from American Type Culture Collection (ATCC).

### FIB-SEM, segmentation, reconstruction, and analysis.

Heart samples were processed with the standard TEM protocol described above. The blocks were processed with the ZEISS Crossbeam 540 (Gemini II column) to collect FIB-SEM micrographs at a 10 nm pixel size (X × Y × Z) using ZEISS Atlas 5 software (Carl Zeiss Microscopy). The 10 nm FIB milling thickness was conducted at 30 keV, while maintaining the beam current at 2 nA. The collected micrographs were aligned using a proprietary algorithm and then exported as 8-bit TIFF files, which were sent to Ariadne (https://ariadne.ai/) for AI-based segmentation of mitochondria. The 3D-reconstructed mitochondria for this study were generated using Dragonfly software, version 2020.2 for Windows 10 (Object Research Systems; http://www.theobjects.com/dragonfly). Analysis was performed using the multi-ROI object analysis tool in Dragonfly.

### RNA microarray.

RNA was extracted from mouse hearts using either the Direct-zol RNA Miniprep Kit (Zymo, catalog R2051) or the RNeasy Fibrous Tissue Mini Kit (QIAGEN, catalog 74704, used for the ASO experiment), and RNA expression was measured using the Clariom_S_Mouse microarray (Affymetrix). Transcriptome Analysis Console software (Affymetrix, version 4.0.1) was used to analyze the data with the default settings.

### ASO experiment.

ASOs were synthesized at Ionis Pharmaceuticals as previously described (73). C10^G58R;^ OMA1^+/–^ mice were given weekly subcutaneous injections of either a nontargeting (control) ASO or 1 of 2 OMA1-targeting ASOs starting at 21 weeks of age. The ASOs were at a concentration of 5 mg/mL, and injections were dosed at 50 mpk. A total of 12 injections were administered per mouse over 12 weeks. Echocardiography was performed during the tenth week, and motor function tests were performed during the 12th (final) week. After the tests were completed, the mice were anesthetized with isoflurane and transcardially perfused with PBS, and their tissue was collected and flash-frozen in liquid nitrogen. Immunoblotting and transcriptomics were performed on heart tissue as described above.

### BN-PAGE.

Mitochondrial isolation from fresh heart tissue was performed as described above, and BN-PAGE was performed as previously described (23).

### Statistics.

FDR *q* values were calculated for the transcriptomics experiments with Transcriptome Analysis Console software (Affymetrix, version 4.0.1) and for the proteomics experiments with Perseus (MaxQuant, using the permutation-based FDR method with s_0_ = 0). All other statistical analyses were performed in GraphPad Prism 9 (GraphPad Software) using Welch’s *t* test corrected for multiple comparisons; 1-way ANOVA with Dunnett’s T3 multiple comparisons (or Šidák’s or Games-Howell’s multiple comparisons); 2-way ANOVA with Šidák’s multiple comparisons; or the log-rank (Mantel-Cox) test for survival data.

### Study approval.

The patient research protocol (REC 20/04, IRAS 162181) was approved and performed under the ethics guidelines issued by the South Central–Berkshire Research Ethics committee for clinical studies (United Kingdom), with written informed consent obtained from all participants including for the publishing of patient photographs. All animal studies were approved by the Animal Care Use Committee of the NINDS, NIH intramural program.

## Author contributions

MKS and DPN conceptualized the study and designed the methodology. MKS, XH, BPW, EF, IS, YL, CKEB, DAS, CF, IAB, LTJ, JP, and DPN performed experiments. MKS, NPR, and DPN conducted formal analysis. AFP, PMQ, CLO, JP, and DPN provided resources. MKS and DPN wrote the original draft of the manuscript. Review and Editing, MKS, XH, JP, PMQ, EF, LTJ, and DPN reviewed and edited the manuscript. MKS and DPN performed visualization. LTJ, JP, and DPN supervised the study. LTJ, JP, and DPN acquired funding.

## Supplementary Material

Supplemental data

Supplemental data set 1

Supplemental data set 2

Supplemental data set 3

Supplemental data set 4

Supplemental video 1

Supplemental video 2

Supplemental video 3

## Figures and Tables

**Table 2:**
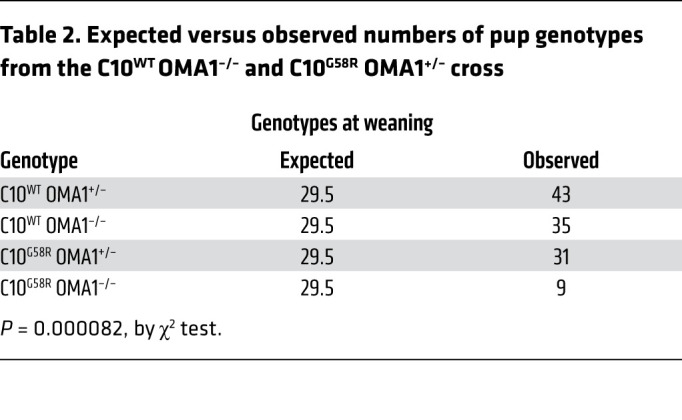
Expected versus observed numbers of pup genotypes from the C10^WT^ OMA1^–/–^ and C10^G58R^ OMA1^+/–^ cross

**Table 1:**
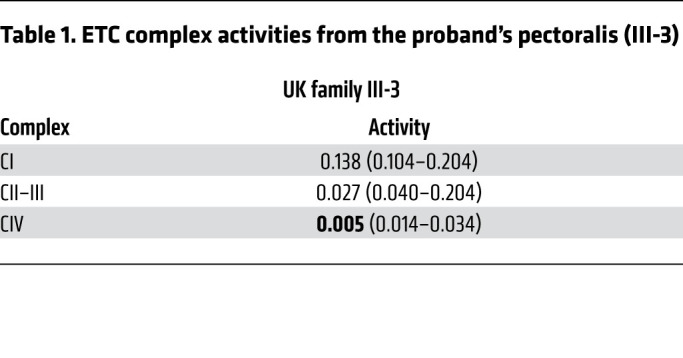
ETC complex activities from the proband’s pectoralis (III-3)

## Version 1 06/14/2022

In-Press Preview

## Version 2 07/15/2022

Electronic publication
