# Supplementary material for: OMA1 mediates local and global stress responses against protein misfolding in CHCHD10 mitochondrial myopathy
Source: J Clin Invest. 2022 Jul 15;132(14):e157504. doi: 10.1172/JCI157504 (PMC9282932; doi:10.1172/JCI157504)

Supplemental Files and Videos

Supplemental data set 1: Echocardiography of mouse hearts

Supplemental data set 2: Proteomics of HEK293 dox-inducible cells and mouse hearts

Supplemental data set 3: Gene expression foldchanges of mouse hearts

Supplemental data set 4: Gene set enrichment analyses of gene expression data

Supplemental Video 1: Mitochondrial membrane potential of untreated and DOX (C10 WT or mutant overexpressing)-treated HEK293 cells

Supplemental Video 2: FIB-SEM segmentation of heart mitochondria

Supplemental Video 3: Segmented non-megamitochondria (shades of cyan) and megamitochondria (differently colored) from the $C10^{G58R}$; $OMA1^{-/-}$ dataset

Resources table

REAGENT or RESOURCE	SOURCE	IDENTIFIER
Antibodies		
CHCHD2 (WB)	Proteintech	Cat#19424-1-AP; RRID: AB_10638907
CHCHD10 (WB, IF 1:250)	Sigma	Cat#HPA003440; RRID: AB_1078348
FLAG M2 (IF 1:1000)	Sigma	Cat#F1804-1MG; RRID: AB_262044
TOM20 F-10 (IF 1:1000)	Santa Cruz	Cat#SC-17764; RRID: AB_628381
Tubulin (WB)	Sigma	Cat#T8328; RRID: AB_1844090

Alpha-tubulin (WB) (Fig. 8C-D and Fig. S10)	Proteintech	Cat#66031-1-Ig; RRID: AB_11042766
NDUFB8 (WB)	Abcam	Cat#Ab110242; RRID: AB_2756818
Cytochrome c (IF 1:200)	BD Biosciences	Cat#556432; RRID: AB_396416
PDH (IF 1:100)	Abcam	Cat#AB110333; RRID: AB_10862029
OPA1 (WB)	BD Bioscience	Cat#612606; RRID: AB_399888
OMA1 (WB)	Santa Cruz	Cat#SC-515788; RRID: AB_2905488
OMA1 (WB) (Fig. 8C-D and Fig. S10)	Cell Signaling	Cat# 95473S; RRID: AB_2800248
HSP90 (WB)	Proteintech	Cat#13171-1-AP; RRID: AB_2120924
eIF2α (WB)	Santa Cruz	Cat#SC-133132; RRID: AB_1562699
p-eIF2α (WB)	Cell Signaling	Cat#3597; RRID: AB_390740
CHOP (WB)	Cell Signaling	Cat#2895; RRID: AB_2089254
HA (WB)	Biolegend	Cat#901514; RRID: AB_2565336
MTHFD2 (WB)	Proteintech	Cat#12270-1-AP; RRID: AB_2147525
ALDH1L2 (WB)	Proteintech	Cat#21391-1-AP; RRID: AB_2878854
MTHFD1L (WB)	Proteintech	Cat#16113-1-AP; RRID: AB_2250974

ALDH18A1 (WB)	Proteintech	Cat#17719-1-AP; RRID: AB_2223896
PYCR1 (WB)	Proteintech	Cat#13108-1-AP; RRID: AB_2174878
COX6A1 (WB)	Abcam	Cat#Ab110265; RRID: AB_10866496
COX7A1 (WB)	Proteintech	Cat#11413-1-AP; RRID: AB_2085705
COX7A2L (WB)	Proteintech	Cat#11416-1-AP; RRID: AB_2245402
SDHA (BN PAGE)	Abcam	Cat#Ab14715; RRID: AB_301433
MTCO1 (BN PAGE)	Invitrogen	Cat#459600; RRID: AB_2532240
p62 (IF, 1:200)	Abcam	Cat#AB56416; RRID: AB_945626
LC3B (WB)	Novus Biologicals	Cat#NB600-1384; RRID: AB_669581
4-HNE (WB)	Abcam	Cat#AB46545; RRID: AB_722490
TOM70 (WB)	Santa Cruz	Cat#SC-390545; RRID: AB_2714192
p32 (WB)	Abcam	Cat#AB24733; RRID: AB_448269
Mic60 (WB)	Proteintech	Cat#10179-1-AP; RRID: AB_2127193
Beta-actin	Sigma	Cat#A2228; RRID: AB_476697
Chemicals, peptides, and recombinant proteins		
BanII Endonuclease	NEB	Cat#R0119S
Platinum SuperFi II Green Master Mix	Invitrogen	Cat#12369010
SYBR Gold	Invitrogen	Cat#S11494

TaKaRa LA Taq Hot Start	Takara Bio	Cat#RR042A
HaeIII Endonuclease	NEB	Cat#R0108T
Cytochrome c from equine heart	Sigma	Cat#C2506
DAB	Sigma	Cat#D5637
Catalase	Sigma	Cat#C40
Mounting medium	KPL	Cat#71-00-16
LipidTOX deep red	Invitrogen	Cat#H34477
Mitotracker Green	Invitrogen	Cat#M7514
MitoSOX Red	Invitrogen	Cat#M36008
CM-H2DCFDA	Invitrogen	Cat#C6827
FuGENE 6	Promega	Cat#PRE2691
Polyethyleneimine (PEI 25K)	Polysciences	Cat#23966
BSA fraction V	MP Biomedicals	Cat#02160069
Xylenes	Sigma	Cat#534056
8% glutaraldehyde	Electron Microscopy Services	Cat#16020
Digitonin	Sigma	Cat#D141
Proteinase K	Bioline	Cat#BIO-37084
Antimycin A	Sigma	Cat#A8674
BAM 15	Selleck Chemicals	Cat#S0411
Critical commercial assays		
BCA kit	Thermo Scientific	Cat#23225
DNeasy Blood and Tissue Kit	QIAGEN	Cat#69504
Direct-zol RNA Miniprep Kit	Zymo	Cat#R2051
RNeasy Fibrous Tissue Mini Kit	QIAGEN	Cat#74704
Deposited data		
Congenital OMA1 KO vs het effect on G58R and S59L microarray	This paper	GEO: GSE189393
Acute OMA1 KD effect on gene expression in the G58R background microarray	This paper	GEO: GSE189396
Experimental models: Cell lines		
HeLa	ATCC	
HEK293	ATCC	
HeLa$^{OMA1\ KO}$	Sekine et al., 2019	N/A
HEK293$^{C2/C10\ DKO}$	Liu et al., 2020	N/A
HEK293 C10 G58R Tet-inducible	Liu et al., 2020	N/A
HEK293 C10 WT Tet-inducible	This paper	
HEK293 C10 S59L Tet-inducible	This paper	
Experimental models: Organisms/strains		
CHCHD10^{G58R} mouse	This paper	N/A
CHCHD10^{S59L} mouse	Liu et al., 2020	N/A
CHCHD2/CHCHD10 DKO mouse	Liu et al., 2020	N/A
OMA1 KO mouse	Quiros et al., 2012	N/A

Software and algorithms		
CalR	Mina et al., 2018	RRID: SCR_015849
Fiji	NIH	RRID: SCR_002285
Perseus	MaxQuant	RRID: SCR_015753
Transcriptome Analysis Console Software	Affymetrix	RRID:SCR_018718
Dragonfly	ORS	
Image Studio	LI-COR	RRID:SCR_015795
Prism 9	GraphPad	RRID:SCR_002798
Other		
Grip strength instrument	BIOSEB	Cat#EB1-BIO-GS3
Rotarod	Ugo Basile	Cat#57624
Treadmill	Columbus Instruments	Cat#1050-RM Exer-3/6
Automated droplet generator	Bio-Rad	Cat#1864101
QX200 droplet reader	Bio-Rad	Cat#1864003
1.5H coverslips	Thorlabs	Cat#CG15KH

Supplemental methods

Generation and genotyping of C10^{G58R} mice

A guide RNA (gRNA) near the G54 sequence in mouse C10 (mouse equivalent of G58) was used (TAGCCGTGGGCTCAGCTGTAGGG), along with a single-stranded donor oligonucleotide with a GGC → AGA substitution at G54 for repair: CTGCCGCTCCCGGCCAGCCGGGTCTTATGGCTCAGATGGCATCCACCGCCGCAG GCGTAGCCGTGAGATCAGCTGTAGGCCATGTCATGGGTAGCGCCCTGACCAGTG CCTTCAGTGGGGGAAATTCAGAGCCTGCCCAGCCTGCCGTCCAGCAGGTGAGCG GGAGGACTCAAGAAACGGAGGCAGGATTCACACATGGT. The following primers

were used for genotyping: forward primer 5'-GACCCTGGAGTAGAGGGGTT-3' and reverse primer 5'-GGCCACTCCTCATTGGACTC-3'. The mouse C10 G54R mutation removes a cut site of the restriction endonuclease BanII (NEB, cat# R0119S), introducing a restriction fragment length polymorphism. Therefore, the region surrounding G54 was amplified by PCR and then subjected to a BanII restriction digest for 1 hour followed by 10 minutes of heat inactivation at 80 C, and finally run on a 2% agarose mini gel. The digested WT bands are ~150bp and the undigested G58R bands are ~300bp.

Motor function tests

$C10^{WT}$ and $C10^{G58R}$ littermates aged 18 weeks and $C10^{S59L}$ mice aged 25 weeks were tested. Mouse forelimb grip strength was measured by pulling the mouse and recording the force generated as the mouse grips to the instrument (BIOSEB instrument with bar, cat# EB1-BIO-GS3). The mouse had to grip with both forelimbs for the trial to be scored; if the mouse used only one forelimb or did not have a proper grip, it was allowed to rest and the trial was repeated. This was repeated 3 times with 15 seconds of rest in between, and the results were averaged.

Balance and motor coordination were tested by placing the mice on a rotating rod (rotarod, Ugo Basile cat# 57624) and measuring the time to fall. The rotarod was allowed to rotate briefly to ensure that all mice faced the proper direction, and when all were facing forwards the speed was ramped from 5 rpm to 40 rpm over 120 seconds;

the trial finished when all the mice had fallen off the rotarod. This was repeated 3 times, with 15 minutes of rest between each trial.

Endurance and fatigue were measured with the treadmill fatigue test. The mice were run on a treadmill (Columbus Instruments cat# 1050-RM Exer-3/6) with gradually increasing speed, and the distance at which the mouse begins spending most of the time in the rear end of the treadmill (defined as 5 consecutive seconds being within a body's length of the shocker) was recorded; this was performed after three days of training. The electric stimulus was at 2 Hz and 1.125 mA. For training, the mice were run for 5 minutes at a speed of 10 meters/minute. This was done once a day for the three days prior to the test. For the fatigue test, mice were run at the following speeds for the specified amounts of time: 30 seconds at 8 m/min, 30 seconds at 10 m/min, 3 minutes at 12 m/min, 4 minutes at 16 m/min, 4 minutes at 20 m/min, 4 minutes at 24 m/min, and then at 28 m/min until the mouse is fatigued. The distance run was recorded when the mice fulfilled the fatigue criterion above.

As $C10^{G58R}$ mice were generally unable to turn when placed facing upwards in the pole test, the test had to be modified so that mice are placed at the top of the 50 cm pole facing downwards and allowed to descend. To train the mice, this was repeated 3 times with 1 minute of rest in between, and this was done each of the two days preceding the experiment. After the two training days, mice were placed at the top of the pole and the time needed until all four paws touch the base was recorded. This was repeated 5 times with 3 minutes of rest between each trial, and the trial results were averaged.

The behavioral tests were performed in the same week as follows – day 1: grip strength, pole test training 1, treadmill training 1; day 2: rotarod, pole test training 2, treadmill training 2; day 3: pole test, treadmill training 3; day 4: treadmill.

Echocardiography and electrocardiography

Mouse heart echocardiography and electrocardiography (ECG) were performed at the NHLBI Phenotyping Core. Mice were lightly anesthetized with isoflurane and placed supine over a heated platform with ECG leads and a rectal temperature probe. The Vevo2100 ultrasound system (VisualSonics, Toronto, Canada) with a 30 MHz ultrasound probe (VisualSonics, MS-400 transducer) was used to acquire heart images. Measurements were made from standard 2D and M mode images from the parasternal long axis and mid-papillary short axis views of the left ventricle.

Cell lines

To generate stable HEK293$^{C2/C10}$ DKO cell lines stably expressing mouse C10, mouse CHCHD10 cDNA was obtained from Transomic (clone: BC061190) and subcloned into the pCIG3-IRES-GFP lentiviral vector (Addgene Plasmid #78264). Cells were transduced as described previously (Liu et al., 2020). Mouse CHCHD10 was also subcloned into the E1YFP-N1 vector (Clontech), replacing EYFP, for transient expression in HeLa cells to assess for mitochondrial fragmentation. Mouse CHCHD10 G54R (equivalent of human G58R) was generated using the QuikChange II Site-Directed Mutagenesis Kit (Agilent).

Mouse complexes I and IV activity assays

30-60 mg samples of liquid-nitrogen-flash-frozen mouse heart or muscle were placed in 1.5 mL centrifuge tubes with 400 μL of Solution 1 from the CIV activity kit. The tissue was minced with scissors into small pieces and then homogenized with a Fisher Scientific PowerGen 125 Homogenizer. Heart was homogenized at power level 4.5 for 1 minute, then 10 seconds at power level 5.5. Muscle was homogenized at power level 4.5 for 1 minute, then 30 seconds at power level 5.5. Protein concentration was determined with a BCA kit (Thermo Fisher Scientific, cat# 23225), and the sample was diluted to 5.5 mg/mL in Solution 1. Detergent was added to the samples for a final volume ratio of 1:10 (e.g. 10 μL Detergent for 90 μL of sample), resulting in a protein concentration of 5 mg/mL. The samples were incubated on ice for 30 minutes then centrifuged at 15,000 g for 20 minutes at 4 C, and the supernatant was collected.

The plates were equilibrated to room temperature before being loaded with samples. For CI activity, heart samples were diluted in incubation solution to 33.75 μg /450 μL, and muscle samples were diluted in incubation solution to 90 μg /450 μL. For CIV activity, heart and muscle samples were diluted to 45 μg/450 μL in Solution 1. 200 μL of diluted sample was added to the respective plate, and the assay was performed in technical duplicates. The plates were incubated at room temperature for 3 hours. Afterwards, wells were emptied and washed thrice with wash buffer (CI) or Solution I (CIV).

For CI, 200 μL of 1X NADH and 1X dye in 1X wash buffer was added to each well, and the plate was placed in a plate reader with the following settings – wavelength: 450 nm,

time: 30 minutes, interval: 30 seconds, temperature: room temperature, shake between readings. The slope of the resulting curve represented CI activity.

For CIV, 200 µL of 1:20 reagent c in Solution 1 was added to each well, and the plate was placed in a plate reader with the following settings – wavelength: 550 nm, time: 120 minutes, interval: 1 minute, temperature: 30 C, shake between readings. The absolute value of the slope of the resulting curve represented CIV activity.

Human mtDNA long-range PCR

Genomic DNA was extracted from fresh frozen tissue by first homogenizing tissue and then using conventional proteinase K digestion and phenol-chloroform extraction methods. Long-range PCR of 13.8 kb of the human mitochondrial genome (NC_012920.1 m.2695 to m.16459) was undertaken using primers 5’-GAGGCGGGCATAACACAGCAAGACGA-3’ and 5’-GGCCCGGAGCGAGGAGAGTAGCAC-3’ (Li et al., 1995) and TaKaRa LA Taq HS (Takara Bio Inc., cat# RR042A). The reaction conditions were as recommended by Takara Bio for a 25 µL reaction, but with 4 µL TaKaRa dNTP mixture, primers at a final concentration of 1 µM, and approximately 100 ng of template genomic DNA. The PCR was run with the following cycle: 94 C for 1.5 minutes, 25 x (98 C for 10 seconds, 68 C for 10 minutes + 30 seconds per cycle), 72 C for 10 minutes, 15 C indefinitely. Following the PCR, 10 µL of product was run on a 0.7% agarose gel for 4 hours at 80 Volts. Gels were imaged using a UV transilluminator.

Mouse mtDNA long-range PCR

Whole DNA was extracted from 15-25 mg of mouse tissue using the DNeasy Blood and Tissue Kit (QIAGEN, cat# 69504) according to the kit's instructions. DNA was then diluted in water to 40 ng/μL. The following primers were used – forward: 5'-CTGGAATTCAGCCTACTAGCAATTATCC-3' and reverse: 5'-TTTAGGTTTATGGCTAAGCATAGTGGGG-3' (Bannwarth et al., 2016). The final PCR reaction contained 10 μL of Platinum SuperFi II Green Master Mix (Invitrogen, cat# 12369010), 5 μL template DNA (40 ng/μL, 200 ng total), and 5 μL of primer master mix (1 μM forward and 1 μM reverse primers in water, for a final reaction concentration of 250 nM each). The PCR was run with the following cycle: 98 C for 2 minutes, 20 x (98 C for 10 seconds, 60 C for 15 seconds, 72 C for 6 minutes and 24 seconds), 10 x (98 C for 10 seconds, 60 C for 15 seconds, 72 C for 6 minutes and 24 seconds + 20 seconds per cycle), 72 C for 5 minutes, 4 C indefinitely.

Following the PCR, the 20 μL samples were diluted in 40 μL of water and run on a 0.8% agarose gel for 1 hour at 70 Volts in TAE buffer and post-stained with SYBR Gold (Invitrogen, cat# S11494) for 30 minutes in TAE buffer. Gels were imaged on a Bio-Rad Chemidoc system.

Mouse mtDNA copy number

Whole DNA was extracted from hearts as described above. Whole DNA was digested with HaeIII (NEB, cat# R0108T) as follows: 5 μL 10X CutSmart buffer, 1 μL HaeIII, 500 ng DNA, and water for a final reaction volume of 50 μL at 37 C and 300 RPM for 15

minutes, followed by a 5-minute heat inactivation at 80 C. DNA was diluted in water from 50 ng/μL to 1 ng/μL. Droplet digital PCR (ddPCR) primer/probe assays for copy number variation (CNV) were obtained from Bio-Rad (mouse ND1/HEX, mouse ActB/FAM). The ddPCR mix was as follows: 3.5 μL digested DNA (1 ng/μL), 1.1 μL ND1 assay, 1.1 μL ActB assay, 5.3 μL water, and 11 μL 2X ddPCR Supermix for Probes. Droplets were generated with the Automated Droplet Generator (Bio-Rad, cat# 1864101), and the reaction was run with CNV settings using the QX200 Droplet Reader (Bio-Rad, cat# 1864003). The resulting data was analyzed with QuantaSoft Analysis Pro (Bio-Rad).

Mouse COX activity stain and fiber width measurements

Mice were anesthetized with isoflurane and transcardially perfused with PBS. Tibialis and soleus muscles were collected and immediately flash-frozen in liquid-nitrogen-cooled isopentane and stored at -80 C until sectioning. The muscles were cryosectioned at -20 C to obtain 10 μm transverse sections. For staining, muscle sections were allowed to dry at room temperature for 1 hour and then were incubated in the incubation solution for 1 hour at 37 C. Incubation solution: 100 μM cytochrome c (Sigma, cat# C2506), 4 mM DAB (Sigma, cat# D5637), and 20 μg/mL catalase (Sigma, cat# C40) in 0.2 M phosphate buffer (pH 7.0). Sections were washed in 0.2 M phosphate buffer for 5 minutes twice, mounted with mounting medium (KPL, cat# 71-00-16), coverslipped, and imaged with a Zeiss Wide-Field microscope. Image analysis was performed in Fiji (NIH). Muscle fiber maximal widths were measured for 20 random fibers per mouse.

Lipid staining

Mice were anesthetized with isoflurane and transcardially perfused with 20 mL PBS followed by 15 mL of 4% paraformaldehyde (PFA). Tibialis muscles were dissected and postfixed in 4% PFA for 30 minutes at room temperature, washed for 5 minutes in PBS 3 times, and stored in PBS at 4 C until use. Longitudinal 35 μm free-floating sections were obtained using the Compresstome VF-300-0Z (Precisionary). Muscle sections were incubated in 1:1000 LipidTOX deep red (Thermo Fisher Scientific, cat# H34477) for 1 hour at room temperature on a rocker and protected from light. The sections were then washed with PBS for 10 minutes 3 times, mounted on slides with mounting medium (KPL, cat# 71-00-16), coverslipped with 1.5H thickness coverslips (Thorlabs, cat# CG15KH), and sealed with nail polish. Sections were imaged on an Olympus FLUOVIEW FV3000 confocal laser scanning microscope.

Indirect calorimetry

The Oxymax-CLAMS setup (Columbus Instruments) was used to assess mouse metabolism. Mice were singly housed in the CLAMS chambers, received food and water *ad libitum* throughout the experiment, and were checked on at least twice a day throughout the 5-day duration of the experiment. Oxygen consumption, carbon dioxide production, food intake, and beam breaks (locomotion) were monitored during the experiment. The resulting data was analyzed using CalR (Mina et al., 2018).

Body composition

Non-invasive measurements of lean tissue and fat were obtained using the EchoMRI NMR machine. Un-anesthetized mice were placed in a clear plastic tube and gently restrained at the end of the tube by using a plunger with air holes. The plunger was fitted to the mouse and tightened gently to minimize movement. The tube was then inserted into the mini-spec port to a premeasured depth, and measurements were collected. The mouse was removed from the tube and returned to the home cage, and the tube was washed and sanitized after each use.

EVcouplings conservation analysis and 3D prediction

The human CHCHD10 protein sequence (Q8WYQ3) was analyzed using the EVcouplings server (https://v2.evcouplings.org/). The b0.2 cutoff yielded the most matched sequences and was used in the subsequent analysis. Outputs included assessment of amino acid frequency for each residue among the identified C10 homologs, as well as structure predictions generated using the EVfold algorithm (Marks et al., 2011 and Hopf et al., 2019). The highest scoring structure was visualized using UCSF Chimera (https://www.cgl.ucsf.edu/chimera/).

Hydrophobicity analysis

Hydrophobicity of the middle α-helix of WT, G58R, and S59L C10 was calculated using ProtScale and the Kyte-Doolittle algorithm (https://web.expasy.org/protscale/).

Soluble/insoluble assay

This assay was performed on cultured cells or on mouse tissue. HEK293 or HeLa cells were harvested by scraping into PBS 24 hours after transfection, whereas mouse tissue was fractionated and the assay was performed on total homogenate, the cytosolic fraction, and the mitochondrial fraction. The mitochondrial and cytosolic fractions were treated with 1% TX-100 in 50 mM Tris, and after 21300 g centrifugation for 10 minutes the supernatant was used as the soluble fraction and the pellet was treated again with 2% SDS in 50 mM Tris buffer. After centrifugation, the supernatant was used as the insoluble fraction. Laemmli buffer was added to both fractions to obtain an equal volume of 1X buffer with 2.5% (v/v) 2-mercaptoethanol. Lysates were boiled at 98 C, separated by SDS-PAGE, and analyzed by immunoblotting.

Mouse histochemistry

Mice were anesthetized with isoflurane and transcardially perfused with 25 mL PBS. The heart midventricular region and tibialis muscle were dissected and postfixed in 4% PFA for 1 hour (heart) or 30 minutes (tibialis) at room temperature, with the heart additionally fixed overnight at 4 C. After fixation, the tissue was washed for 5 minutes in PBS 3 times. Some heart specimens were sent to Histoserv (Germantown, MD) for paraffin embedding and microtome sectioning to obtain 4 μm sections. H&E and Masson’s trichrome stains were additionally performed by Histoserv.

35 μm free-floating sections of heart and muscle were obtained using the Compresstome VF-300-0Z (Precisionary). The sections were permeabilized and

blocked in 0.4% Triton-X (Sigma, X100) and 4% BSA fraction V (MP Biomedicals, cat# 02160069) in PBS for 2 hours at room temperature. Afterwards, the sections underwent primary antibody incubation in 0.3% Triton-X and 1% BSA in PBS overnight at 4 C (note: samples from the PDH/C10 experiment from Figure S4E were incubated in primary antibodies for 3 days). The samples were washed for 10 minutes in PBS 3 times, and then incubated in secondary antibodies in PBS (1:500). Afterwards, the samples were washed for 10 minutes in PBS 3 times, mounted on a slide with mounting medium (KPL, cat# 71-00-16), coverslipped (Thorlabs, cat# CG15KH), and sealed with nail polish.

The p62/C10 staining from Figure S9C was performed on unstained 4 μm paraffin-embedded heart sections obtained from Histoserv. The staining procedure was identical to that in the main methods, but the slides had to be deparaffinized first with the following serial incubations: 2x5 minutes xylenes (Sigma, cat# 534056) twice, 3 minutes 1:1 xylenes:ethanol, 2x3 minutes 100% ethanol, 3 minutes 95% ethanol, 3 minutes 70% ethanol, 3 minutes 50% ethanol, 30 seconds rinse in tap water.

Proteinase K protection assay

About 15 μg of mitochondria was lysed in a buffer containing 250 mM sucrose, 10 mM HEPES, 10 mM KCl, 2 mM $MgCl_2$, and 1 mM EDTA (pH 7.4). According to different treatment conditions, lysates were added with or without different percentages of digitonin or 1% Triton X-100. After sitting on ice for 10 minutes the proteinase K was added for a final concentration of 50 μg/mL and the lysates were kept on ice for another

15 minutes. Finally, 3 mM PMSF was added to stop the proteinase K digestion. The samples were mixed with 4X Laemmli sample buffer and 2-mercaptoethanol and analyzed by western blotting.

Transmission electron microscopy

Human pectoralis muscle was snap frozen in liquid-nitrogen-cooled isopentane and subsequently processed for electron microscopy according to standard protocols.

Mouse heart and skeletal muscle TEM was performed as previously described (Liu et al., 2020). For the serial TEM sections, the samples were prepared with a modified protocol for heavy membrane staining. Heart samples were obtained and fixed with glutaraldehyde as above. The samples were placed in filtered 1% tannic acid solution in EM buffer on ice for 1 hour. The samples were then washed and placed in a solution of 1% osmium tetroxide and 1% ferrocyanide in EM buffer. After that, the samples were washed and processed en bloc as above with uranyl acetate, dehydration, embedding, serial sectioning, and imaging.

Immunoblotting

HEK293 and HeLa cells were lysed in a buffer containing 1% sodium dodecyl sulfate (SDS) and 63 mM Tris (pH 6.95). DNA shearing was accomplished using a bath sonicator. Insoluble material was separated by centrifugation at 21130 g at 4 C for 3 minutes and the soluble fraction was retained. Protein concentration was measured

using the BCA assay. Bromophenol blue (final concentration 0.0008%), glycerol (11%) and β-mercaptoethanol (0.284 mM) were added to the supernatant. The sample was heated at 80 C for 10 minutes. Insoluble material was separated by centrifugation at 21130 g at room temperature for 3 minutes and the soluble fraction was retained. Subsequently, the soluble fraction was analyzed by sodium dodecyl sulfate-polyacrylamide gel electrophoresis (SDS-PAGE) and immunoblotting.

Mouse tissue was lysed in a buffer containing 20 mM Tris pH 7.8, 137 mM NaCl, 2.7 mM KCl, 1 mM $MgCl_2$, 1% Triton X-100, 10% glycerol, 1 mM EDTA, and 1 mM dithiothreitol, with 1% proteinase inhibitor. Lysates were sonicated 4 times by a Vibra-Cell Ultrasonic Disruptor for 15 seconds each time, at an output level of 20. Protein concentration was determined by the BCA assay. Lysates were separated on SDS-PAGE gels and analyzed by immunoblotting.

Densitometry measurements were performed using Fiji (NIH) and Image Studio (LI-COR). OMA1-cleaved S-OPA1 bands were calculated by measuring the maximum intensity of each of the five bands in a linescan of the optical density of the five OPA1 bands on the blot using Fiji. After subtracting background intensity, the peaks of the c and e bands were summed and divided by the sum of the five bands (a–e) to obtain the percentage of OMA1-generated S-OPA1 from total OPA1.

Measurements of mitochondrial membrane potential and ROS

To assess mitochondrial potential stability, Tet-inducible HEK293 C10 WT, G58R, or S59L cells also expressing mito-mEmerald were either untreated or treated with 1

µg/mL doxycycline for 48 hours. The cells were then stained with 40 nM TMRE for at least 15 minutes and imaged live using an Olympus FLUOVIEW FV3000 confocal microscope in galvano mode to visualize membrane potential fluctuations. Images were obtained every ~9.5 seconds for at least 90 seconds. Using the mito-mEmerald channel, 20 mitochondria per FOV (60 mitochondria total from 3 biological replicates) were randomly selected and their TMRE/mito-mEm intensities were tracked for 10 frames. The TMRE/mito-mEm signal for each frame for each mitochondrion was normalized by the respective mitochondrion's 10-frame TMRE/mito-mEm average. The normalized TMRE/mito-mEm signals are plotted in Figure 6I, and the 10-frame normalized TMRE/mito-mEm standard deviation for each mitochondrion is represented in Figure 6H.

To assess $\Delta\Psi_m$ by flow cytometry, Tet-inducible HEK293 C10 G58R cells were treated overnight with DMSO or 1 µg/mL doxycycline. The cells were trypsinized and counted. Equal numbers of cells were incubated with 5 nM TMRE and 200 nM MitoTracker green for 15 minutes in a round bottom plate kept in the 37 C tissue culture incubator. After incubation the plate was spun at 400 g for 3 min, the cells were resuspended in a cell sorting buffer described previously (Liu et al., 2020), and their membrane potential was measured using a CellStream (Luminex) flow cytometer. For each sample, TMRE was measured separately for BFP+ cells (which contain the C10 WT or C10 G58R expression cassette) and a small BFP- negative population (which does not contain the expression cassette). The TMRE signal of BFP- cells was used for normalization. For Figure 6E, the stably transduced HEK 293 WT and C2/C10 DKO cells lines were stained with only 5 nM TMRE and the TMRE signal from GFP- cells was used for

normalization. For ROS assessment, Tet-inducible HEK293 C10 G58R cells were treated overnight with DMSO or 1 μg/mL doxycycline. The cells were then trypsinized, counted, and equal numbers were incubated in media containing 5 μM MitoSOX or 10 μM H_2DCFDA for 30 minutes in suspension. The cells were pelleted by centrifugation at 400 g for 3 minutes and then resuspended in a cell sorting buffer. MitoSOX or H_2DCFDA signal intensity was measured using the CellStream flow cytometer. All cell lines were incubated at 37 C with 5% CO_2 and ambient O_2.

Generation of primary fibroblasts

WT and G58R fibroblast cells were generated from P1 newborn pups. The pups were placed in Dulbecco's modified Eagle medium (DMEM) + 1% L-glutamine + 15% fetal bovine serum (FBS) + MEM non-essential amino acid solution (MEM-NEAA) + 1% sodium pyruvate + 1X penicillin/streptomycin (pen/strep) + 1X gentamicin +1X amphotericin B. Skin from the pups was cut into thin 1-2 mm slices using sterile scalpels. The tissues were then transferred to a plate coated with 1% gelatin and containing the dissection media and covered with sterile cover slips. High-glucose DMEM + sodium pyruvate + 10% FBS + pen/strep was used for primary fibroblasts after the first passage.

Mitochondrial isolation

The method was adapted from a previously-published protocol (Frezza et al., 2007). Mice were anesthetized with isoflurane, transcardially perfused with 25 mL PBS, and

the heart was dissected and placed in a dish with cold PBS. The heart was then transferred into a tube with 10 mM EDTA in PBS and was minced into small pieces with scissors. The sample was centrifuged for 30 seconds at 5000 g and was washed twice with PBS/EDTA solution. After the washes, PBS/10 mM EDTA/0.05% trypsin solution was added and the tube was placed on a rocker for 30 minutes at 4 C. The tube was centrifuged at 200 g for 5 minutes and the supernatant was discarded. The tissue was weighed and 10 times the volume of the IBm1 buffer (6.7 mL of 1 M sucrose, 5 mL of 1 M KCl, 5 mL of 1 M Tris/HCl, 1 mL of 1 M EDTA, 2 mL of 10% BSA with water to make 100 mL of buffer, pH 7.4) was added before tissue was homogenized. The homogenate was centrifuged at 700 g for 10 minutes and the supernatant was collected, and it was then centrifuged at 8000 g for 10 minutes. The cytosolic fraction (supernatant) was discarded and the pellet was resuspended in the IBm2 buffer (25 mL of 1 M sucrose, 1 mL of 1 M Tris/HCl, 3 mL of 0.1 M EGTA/Tris with water to make 100 mL of buffer, pH 7.4) and centrifuged again at 8000 g for 10 minutes. The pellet was washed once more in IBm2 buffer before it was resuspended in RIPA buffer with protease inhibitor and phosphatase inhibitor. The sample was sonicated and spun at the highest speed (21130 g) for 10 minutes and the supernatant was collected and processed for BCA analysis.

Mouse transmission electron microscopy

Mice were anesthetized with isoflurane and transcardially perfused with 25 mL of PBS. Hearts and tibialis muscles were rapidly dissected and a 1 mm x 1 mm x 1 mm sample was drop fixed in 4% glutaraldehyde (Electron Microscopy Services, cat# 16020) in electron microscopy (EM) buffer (0.1 M sodium cacodylate at pH 7.4 with 2 mM calcium

chloride) for 1 hour at room temperature and then at 4 C for at least 24 hours. Samples were washed with EM buffer and treated with 1% osmium tetroxide in 0.1 M sodium cacodylate buffer at pH 7.4 for 1 hour on ice, washed and en bloc stained with 0.25% uranyl acetate in 0.1 M acetate buffer at pH 5.0 overnight at 4 C, dehydrated with a graded series of ethanol washes and finally embedded in epoxy resins. Ultrathin sections (70 nm) were stained with lead citrate and imaged with a JEOL 1200 EXII TEM.

Immunogold-electron microscopy

The method was adapted from a previously-published protocol (Tao-Cheng et al., 2021). Briefly, DOX-inducible HEK293 cells were treated with 1 μg/mL doxycycline overnight and then fixed with 4% paraformaldehyde + 0.05% glutaraldehyde for 45 minutes in PBS. The sample was then blocked and made permeable with 0.1% saponin with 5% bovine serum albumin (BSA) for 40-60 minutes. The sample was incubated with CHCHD10 (Sigma, cat# HPA003440) primary antibody followed by secondary antibodies (Nanogold, Nanoprobes, Yaphank, NY) for 1-2 hours, postfixed with 2% glutaraldehyde in PBS at least overnight, silver enhanced (HQ kit, Nanoprobes), en bloc mordanted with 0.25-0.5% uranyl acetate in acetate buffer (pH 5.0) for 1 hour, treated with 0.2% osmium tetroxide in 0.1 M phosphate buffer (pH 7.4) for 30 minutes, dehydrated with a series of graded ethanol, and embedded in epoxy resin. Imaging was performed on a JEOL-1200 EXII TEM.

Oxygen consumption

Seahorse Extracellular Flux Analyzer XF (Agilent) was used to measure the oxygen consumption rates (OCR) of 293 WT or OMA1 KO cells stably transduced with the pCIG3-IRES-EGFP empty vector or C10 G58R. 30,000 cells per well were seeded in XF96 cell culture microplates coated with poly-lysine and incubated for 24 hours at 37 C with 5% CO2 in standard complete high glucose DMEM. One hour before the assay, the cells were placed in DMEM lacking bicarbonate (XF DMEM medium pH 7.4 (Agilent cat# 103575-100)) and supplemented with 10 mM glucose, 1 mM pyruvate, and 2 mM glutamine. The culture plates were moved to a 37 C incubator with atmospheric CO2 to degas for 1 hour prior to the assay. 2-2.5 μM oligomycin, 2 μM FCCP and 0.5 μM of rotenone and antimycin were added during the assay as the OCR was being measured. The data was normalized by protein concentration using the BCA assay.

RNA microarray analysis

In Transcriptome Analysis Console, data were summarized using the gene-level signal space transformation - robust multiple-array average normalization (SST-RMA) method. DEGs were required to have a genome-wide q-value of < 0.01 or 0.05 (as specified) measured using the eBayes analysis of variance (ANOVA) method. Heatmaps were generated using the ComplexHeatmap package in R. The genes displayed in the heatmaps consisted of ATF4, other transcription factors involved in the ISR, ATF4 target genes, and myc (Fusakio et al., 2016; Han et al., 2013; Huggins et al., 2016; Patel et al., 2019; Quiros et al., 2017). GSEA was performed using the GSEA software

and reactome pathways (Jassal et al., 2019; Mootha et al., 2003; Subramanian et al., 2005). A separate GSEA was performed using reactome pathways plus a custom "G58R Upregulated" category of genes that were upregulated with an FDR q-value < 0.01 in the $C10^{G58R}$; $OMA1^{+/-}$ vs $C10^{WT}$; $OMA1^{+/-}$ comparison.

Proteomics and analysis

Mitochondria were isolated from newly harvested mouse hearts as described above. Protein concentration of the mitochondrial fraction was determined using a BCA kit. 86 μg of the mitochondrial fraction was solubilized in 1% digitonin and complexes were separated on a blue native (BN)-PAGE gel with bovine heart mitochondria used as a molecular weight standard. One gel band was cut for each lane. In-gel proteins were reduced with 10 mM Tris(2-carboxyethyl)phosphine hydrochloride (TECP), alkylated with 10 mM N-Ethylmaleimide (NEM), and digested with trypsin. The extracted peptides were desalted and used for label-free quantitation. Liquid chromatography-tandem mass spectrometry (LC-MS/MS) data acquisition was performed on an Orbitrap Lumos mass spectrometer (Thermo Fisher Scientific) coupled with a 3000 Ultimate high-pressure liquid chromatography instrument (Thermo Fisher Scientific). Peptides were separated on an ES802 column (Thermo Fisher Scientific) with the mobile phase B (0.1% formic acid in acetonitrile) increasing from 3 to 24% over 95 minutes. The LC-MS/MS data were acquired in data-dependent mode. For the survey scan, the mass range was 400-1500 m/z; the resolution was 120 k; the automatic gain control (AGC) value was 8e5. The MS1 cycle time was set to 3 seconds. As many MS2 scans as possible were acquired within the cycle time. MS2 scans were acquired in ion-trap with

an isolation window of 1.6 Da. Database search and mutant/WT ratio calculation were performed using Proteome Discoverer 2.4 (Thermo Fisher Scientific) against Sprot Mouse database. Proteins were annotated as mitochondrial if they appeared in mouse MitoCarta3.0, and were additionally annotated with MitoPathways from MitoCarta3.0 (Rath et al., 2021). Identified proteins tagged as non-mitochondrial or without quantifications in at least 3 (Figures 6C and 11A) or 2 (Figure S14F) samples in at least 1 group were filtered out using Perseus (MaxQuant).

For the complex IV monomer vs supercomplexes experiment, the BN-PAGE gel was cut into 4 slices as in Figure S14E and proteomics analysis was performed on each slice as above.

BN-PAGE

The mitochondrial fraction was resuspended in 10 mM of HEPES pH 7.6 and 0.5 M sucrose. Protein concentration was measured using the BCA assay. The mitochondrial fraction was solubilized in 1% n-Dodecyl-β-D-Maltoside (DDM) and 1X NativePAGE sample buffer. The NativePAGE Novex Bis-Tris Gel System (Thermo Fisher Scientific) was used according to the manufacturer’s instructions with the following modifications: 20 µg of the mitochondrial fraction was loaded, only Light Blue Cathode Buffer was used, and electrophoresis was performed at 150 V for 1 hour then 250 V for 2 hours. Polyvinylidene fluoride (PVDF) was used as the membrane for immunoblotting, and transfer was performed at 130 V for 1 hour at 4 C. The membrane was then washed with 10% acetic acid for 20 minutes and air-dried. Afterwards, the membrane was

washed 5 times with methanol to remove residual Coomassie Blue dye, blocked with 5% milk in TBST for 1 hour at room temperature and blotted for proteins of interest.

Supplemental Figure 1

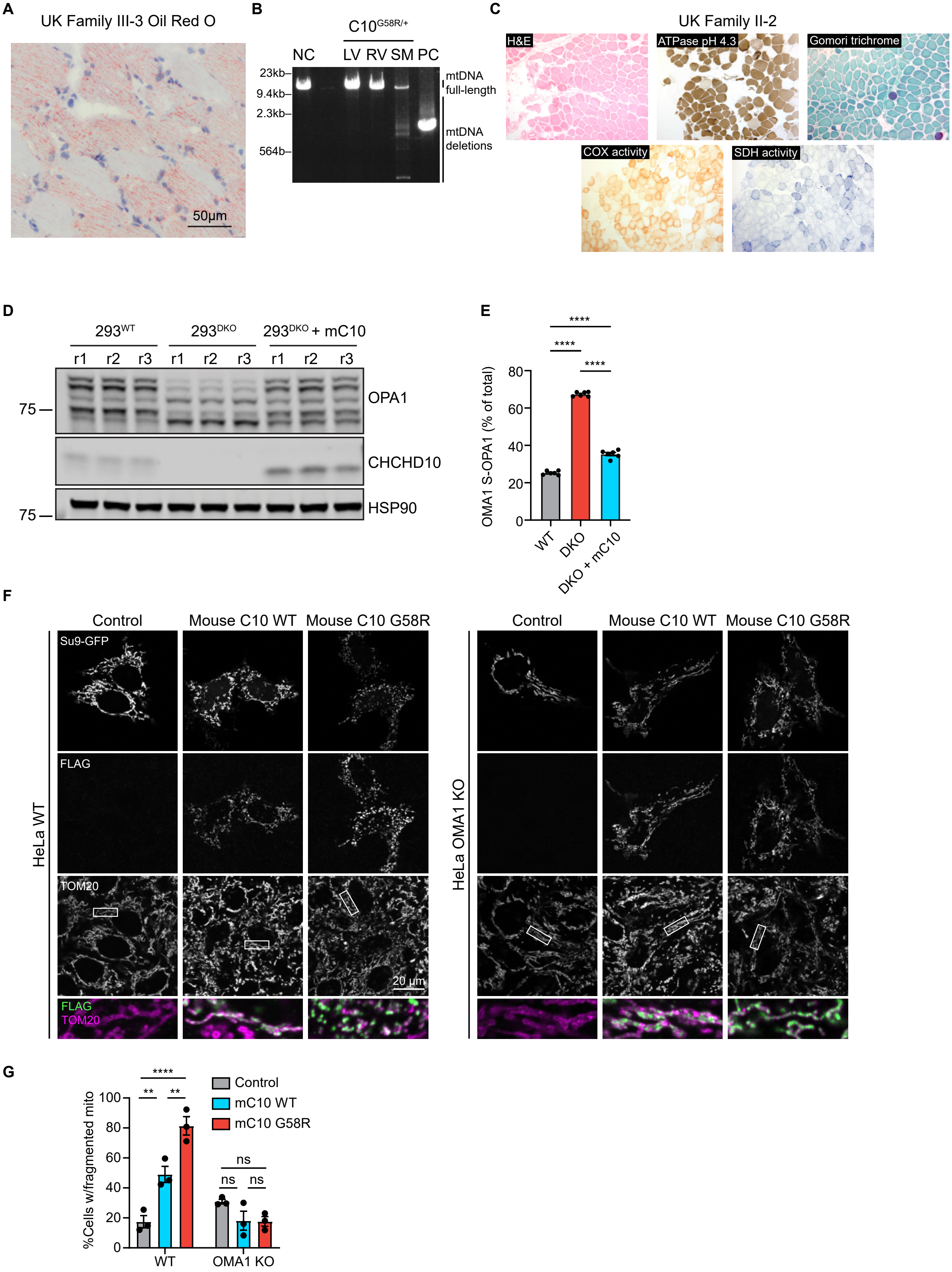

Supplemental Figures

Figure S1. Phenotypic characterization of the G58R mutation in human and cell culture.

(A) Oil Red O of pectoralis muscle of the proband (III-3) showing significantly increased lipid droplets. (B) Long-range PCR of mtDNA of negative control (NC); proband's left ventricle (LV), right ventricle (RV), and skeletal muscle (pectoralis, SM); and positive control (PC). (C) Histochemistry of proband's mother's (II-2) vastus lateralis showing COX-negative fibers and occasional ragged-red fibers. (D) Representative western blot of the effect of stable mouse C10 expression on OPA1 cleavage in HEK293$^{C2/C10\ DKO}$ cells. (E) Quantification of (D) (n = 6 biological replicates). (F) Representative confocal images of HeLa cells transfected with Su9-GFP (control), mouse C10 WT + Su9-GFP, or mouse C10 G58R + Su9-GFP. (G) Quantification of mitochondrial fragmentation from (F) (n = 3 biological replicates).

Supplemental Figure 2

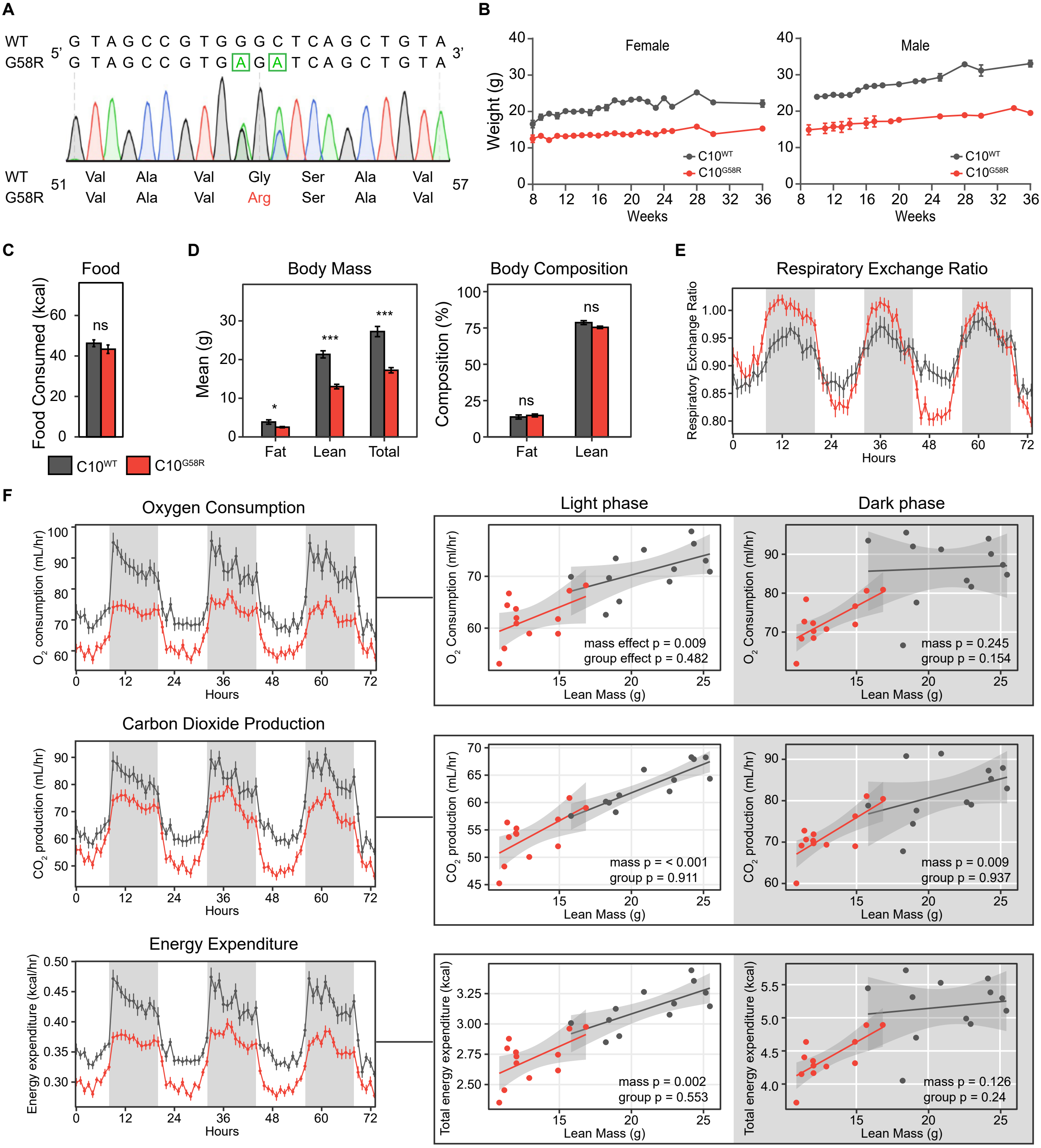

Figure S2. Phenotypic characterization and CLAMS of the G58R mutation in the KI mouse.

(A) Sanger sequencing of C10 from a $C10^{G58R}$ mouse showing the heterozygous G58R mutation. (B) Weights of $C10^{WT}$ and $C10^{G58R}$ mice. At least 3 mice per group per timepoint. At least 10 mice per group total. (C) Total food intake over 72 hours, (n = 12 mice per genotype). (D) Absolute (left) and relative (right) lean and fat mass, (n = 12 mice per genotype). (E) Respiratory exchange ratio hourly plot, (n = 12 mice per genotype). (F) Hourly plots (left) and generalized linear model regression (right) of oxygen consumption, carbon dioxide production, and energy expenditure, (n = 12 mice per genotype).

Supplemental Figure 3

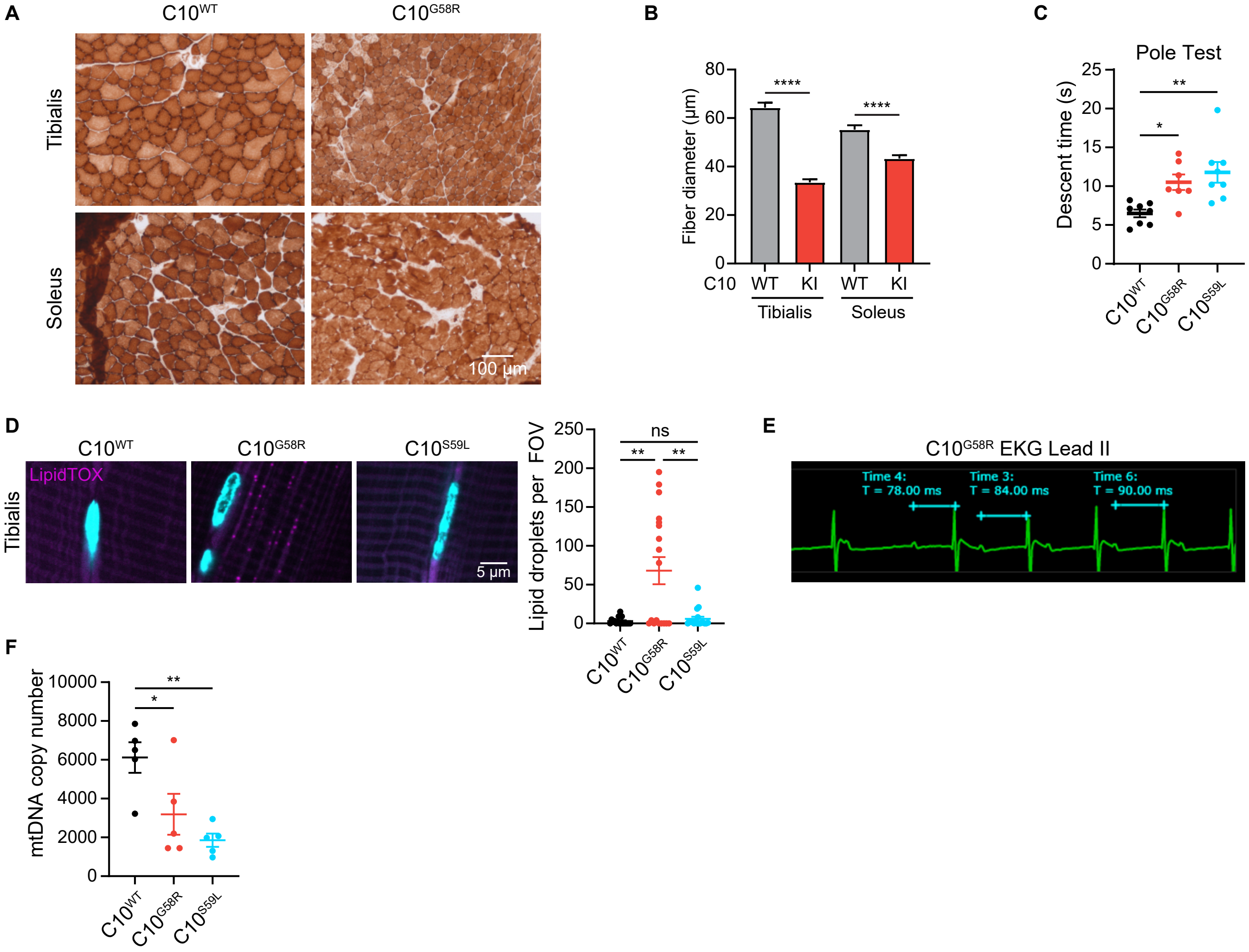

Figure S3. Muscle and EKG characteristics of G58R mice.

(A) Representative COX activity stain of 36-week-old C10^{WT} and C10^{G58R} mouse skeletal muscles, (at least 3 FOV per mouse from 3 mice per group). (B) Quantification of fiber diameter from (A), (n = 60 fibers from 3 mice for C10^{WT} tibialis and C10^{G58R} soleus, and n = 40 fibers from 2 mice for C10^{WT} soleus and C10^{G58R} tibialis). (C) Time to descend a 50 cm pole for 18-week-old C10^{WT} and C10^{G58R} mice, and 25-week-old C10^{S59L} mice. (D) Left: representative images of LipidTOX staining of 30-week-old C10^{WT}, C10^{G58R}, and C10^{S59L} tibialis muscles on the OMA1$^{+/-}$ background. Right: quantification of left, (n = 3 mice per genotype; 6 FOV per mouse). (E) EKG lead II of a C10^{G58R} mouse showing second-degree atrioventricular block. (F) mtDNA copy number of 36–46-week-old C10^{WT}, C10^{G58R}, and C10^{S59L} mouse hearts. Each datapoint represents a mouse.

Supplemental Figure 4

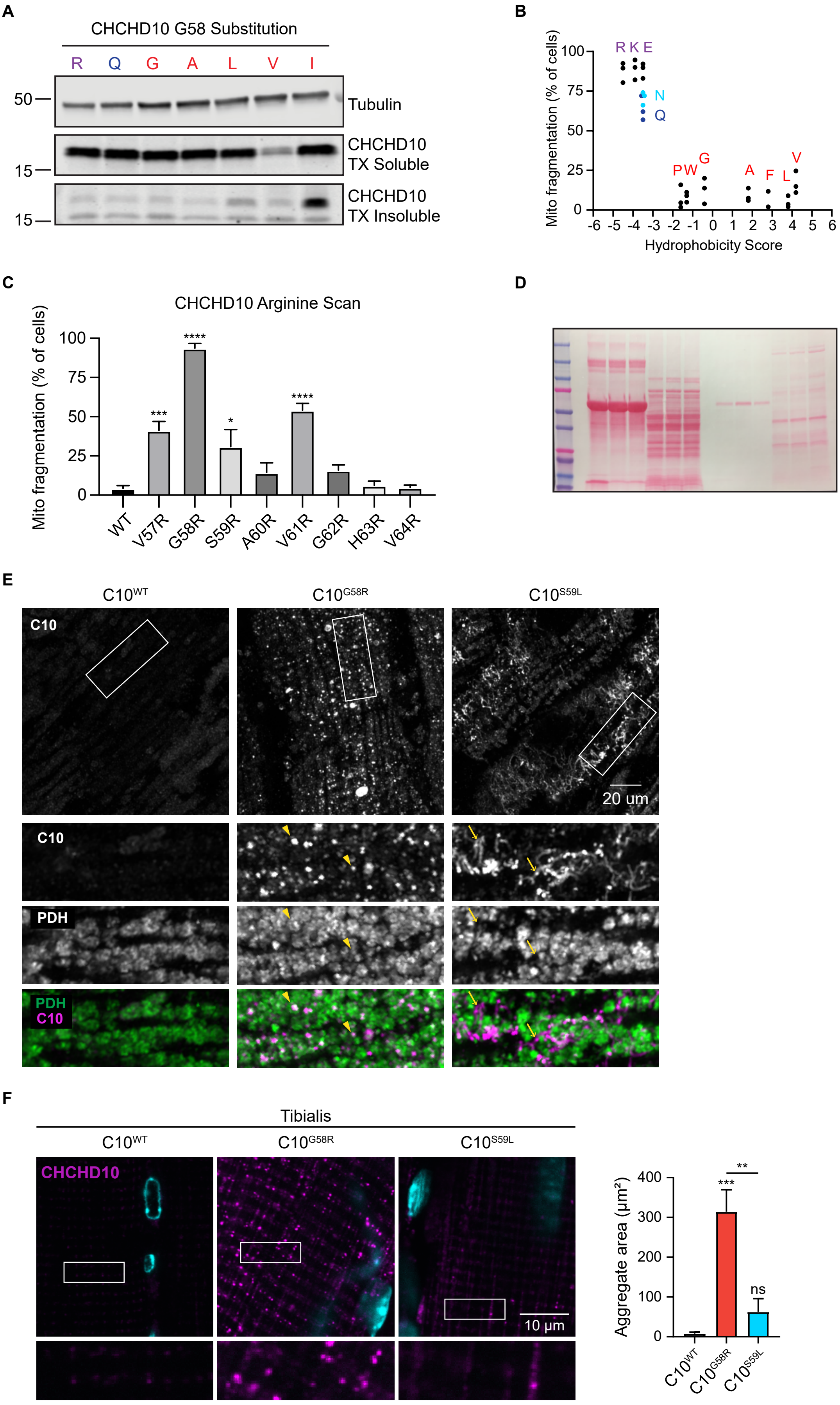

Figure S4. Effect of C10 mutations on C10 solubility, aggregation, and mitochondrial fragmentation.

(A) Immunoblots of Triton-X (TX)-soluble and -insoluble C10 from HEK293 C2/C10 DKO lysates after transfection with C10 containing G58 substitutions with amino acids of varying hydrophobicity. (B) Quantification of mitochondrial fragmentation in HeLa cells transfected with C10 containing G58 substitutions with amino acids of varying hydrophobicity, showing individual datapoints ($n \geq 50$ cells per replicate from 3 biological replicates). (C) Levels of mitochondrial fragmentation in HeLa cells transfected with C10 constructs containing the indicated arginine substitutions in the central α-helix. (D) Loading control for Figure 3A. (E) Airyscan images of 15-week-old $C10^{WT}$, $C10^{G58R}$, and $C10^{S59L}$ mouse hearts stained for C10 and PDH. Arrowheads show intramitochondrial aggregates and arrows show extramitochondrial aggregates. (F) Left: representative confocal images of staining for C10 in tibialis of 30-week-old $C10^{WT}$, $C10^{G58R}$, and $C10^{S59L}$ mice on the $OMA1^{+/-}$ background. Right: quantification of C10 aggregate area, ($n = 3$ mice per genotype; 6 FOV per mouse).

Supplemental Figure 5

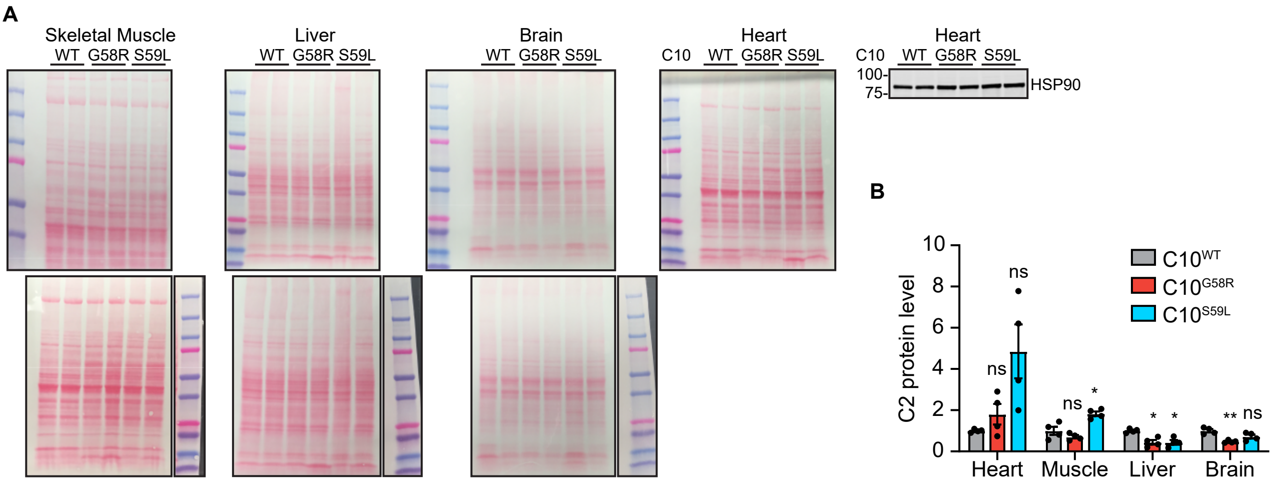

Figure S5. Effect of the C10 G58R mutation on C2 levels.

(A) Loading controls for OPA1 and OMA1 (top) and C2/C10 (bottom) in Figure 5A. (B) Quantification of C2 levels from Figure 5A.

Supplemental Figure 6

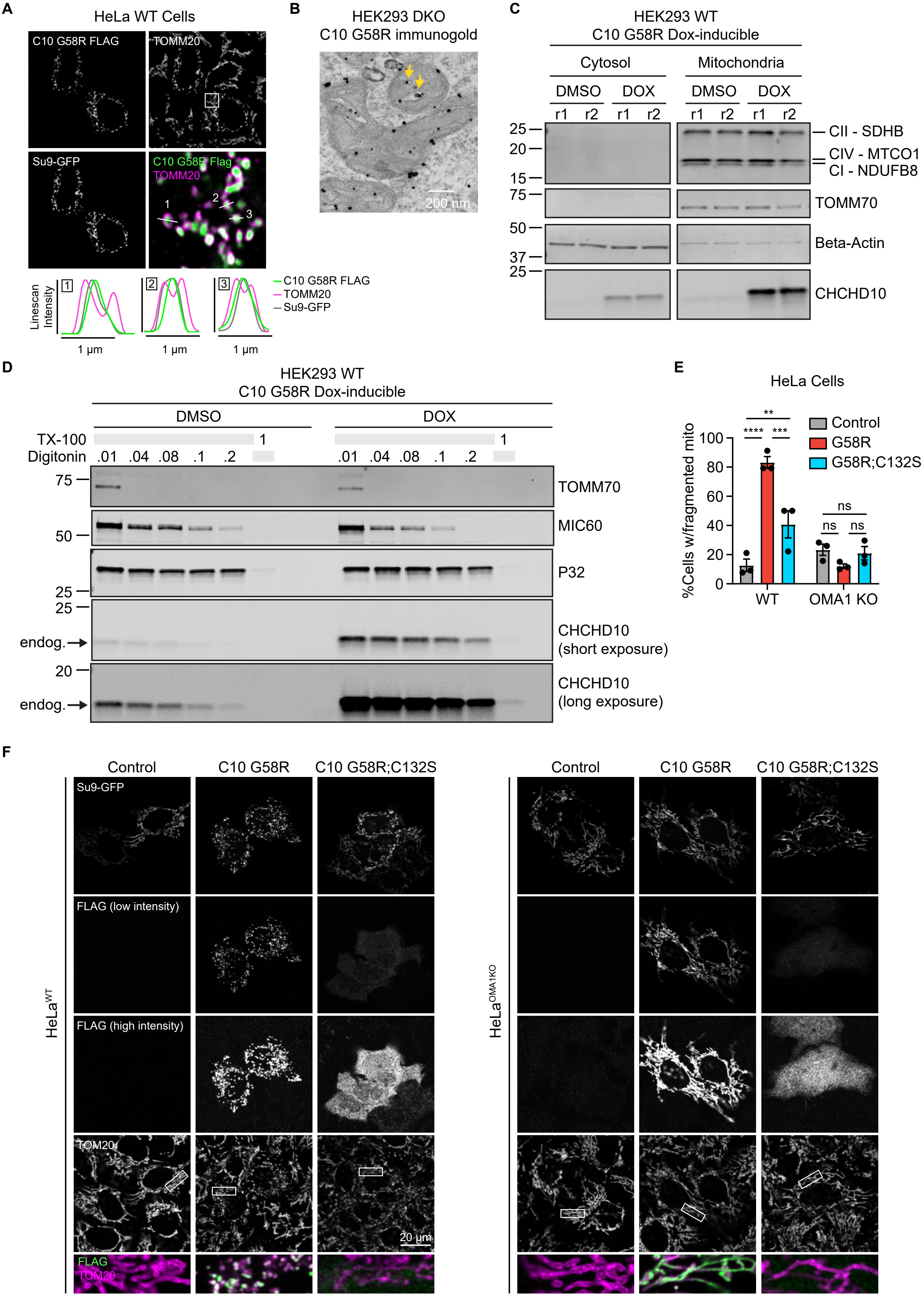

Figure S6. Effect of the C10 G58R mutation on mitochondrial localization of C10 G58R.

(A) Line scans of HeLa cells transfected with Su9-GFP and C10 G58R. (B) Immunogold-EM of C10 G58R overexpressing (OE) C2/C10 DKO HEK293 cells, (representative of ≥ 5 mitochondria in 1 biological replicate). (C) Western blots of the cytosolic and mitochondrial fractions of C10 G58R Dox-inducible HEK293 cells treated with DMSO or doxycycline (1 μg/mL) overnight. (D) Protease protection assay of mitochondria from (C). (E) Quantification of mitochondrial fragmentation in HeLa cells transfected with Su9-GFP (control), C10 G58R + Su9-GFP, or C10 G58R ; C132S + Su9-GFP (n = 3 biological replicates). (F) Representative confocal images of the HeLa cells quantified in (E).

Supplemental Figure 7

A

C10 Expression in Human Cell Lines and Tissues

Tissue/Cell lines	nTPM	% of Heart
Cell RNA - BJ fibroblasts	11.9	0.9%
Cell RNA - HeLa	48.8	3.7%
Cell RNA - HEK293	140.6	10.6%
Tissue RNA - skin	72.4	5.4%
Tissue RNA - sk. muscle	1231.0	92.4%
Tissue RNA - heart muscle	1332.6	100.0%

Data from the Human Protein Atlas (HPA)

B

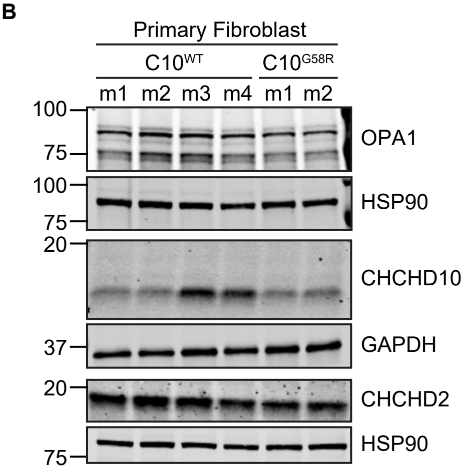

C

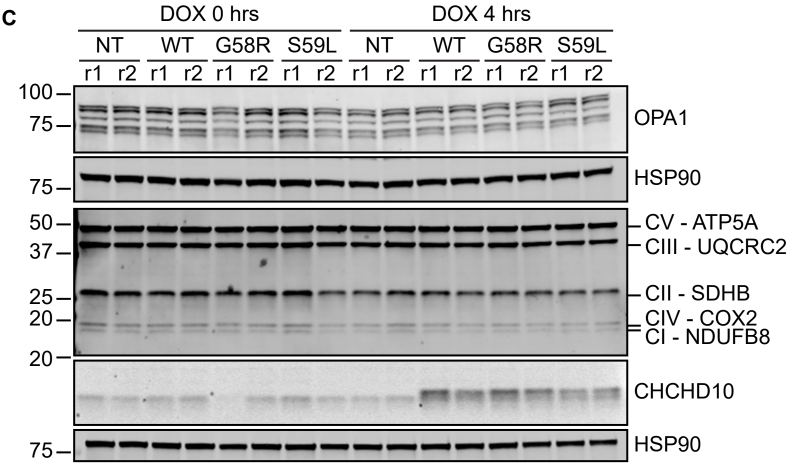

D

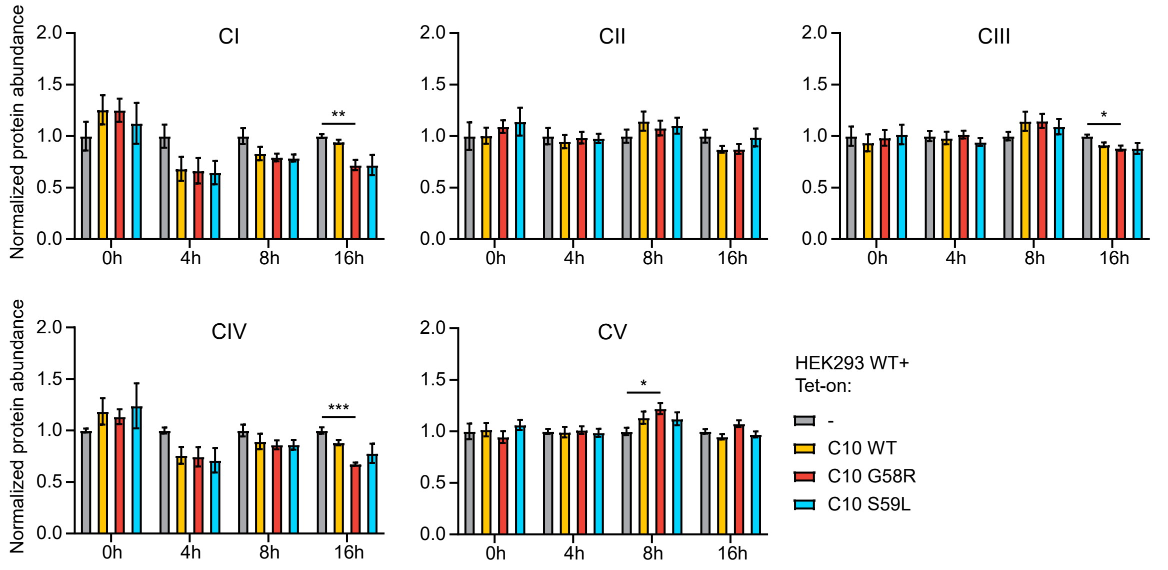

Figure S7. Effect of C10 pathogenic mutations on ETC complex stability.

(A) Normalized expression of CHCHD10 in various cell lines and tissues from the Human Protein Atlas. (B) Western blot of $C10^{WT}$ and $C10^{G58R}$ primary fibroblast lysates. (C) Western blot showing WT HEK293 cell line or dox-inducible HEK293 cell lines for C10 WT, C10 G58R, or C10 S59L expression at the 0- or 4-hour doxycycline (1 μg/mL) treatment timepoints. (D) Quantification of ETC complex subunits from (C). All comparisons are vs. the HEK293 WT cells (gray) (n = 4-5 biological replicates).

Supplemental Figure 8

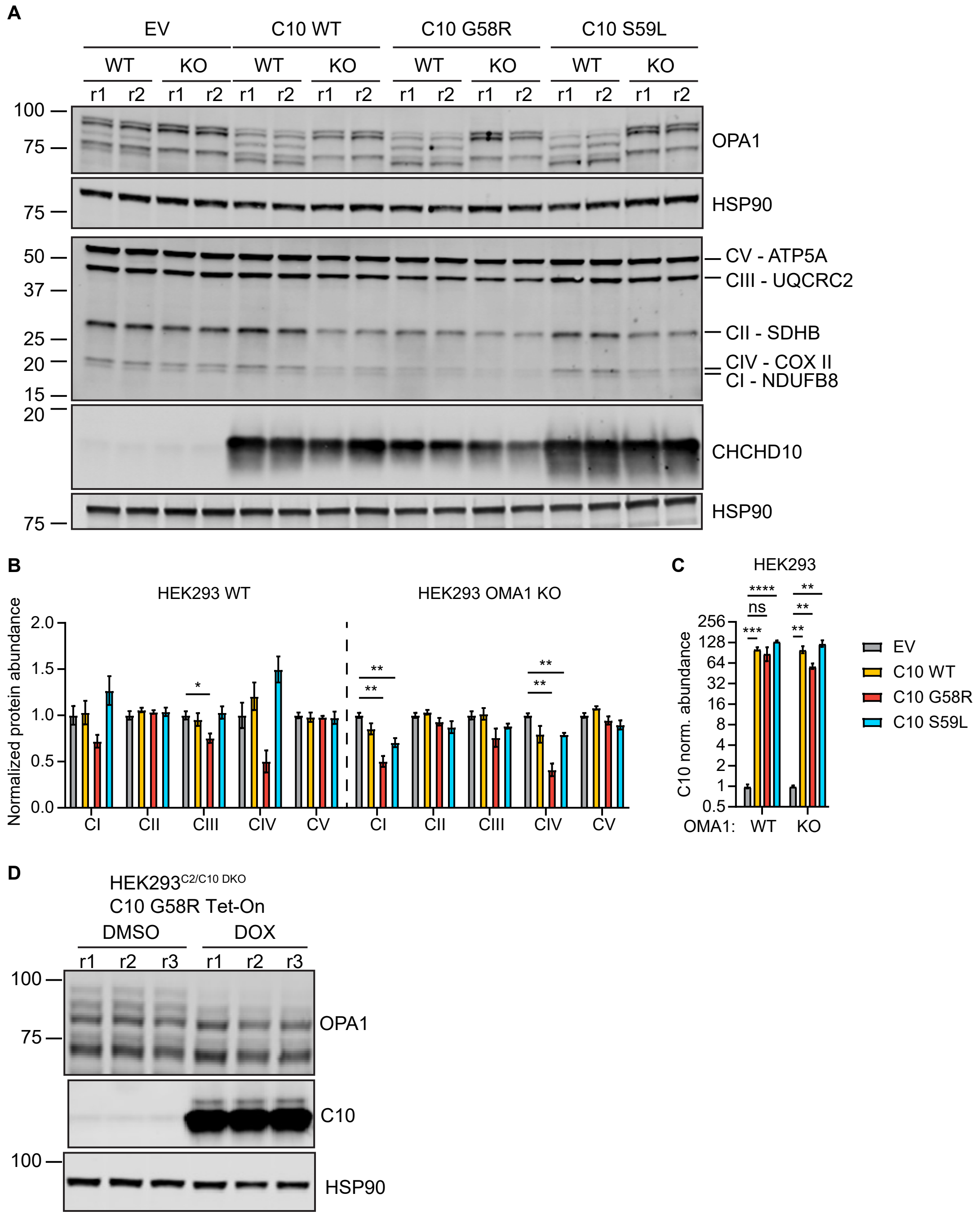

Figure S8. Effect of C10 pathogenic mutations on ETC complex stability under WT and OMA1 KO conditions, and effect of $\Delta\Psi_m$ modifiers on OPA1 cleavage.

(A) Western blot of HEK293 WT and OMA1 KO cells transfected with an empty vector, C10 WT, C10 G58R, or C10 S59L. Quantification of ETC subunit abundances (B) and C10 abundance (C) from (A). All comparisons are vs. the empty vector (n = 4 biological replicates). (D) Immunoblot of OPA1 levels from Tet-On HEK 293 cells treated overnight with DMSO or DOX (overexpressing C10 G58R) on a C2/C10 DKO background.

Supplemental Figure 9

A

Neonatal genotypes

Genotype	P1	P5
C10^{WT};OMA1$^{+/-}$	6	6
C10^{WT};OMA1$^{-/-}$	6	9
C10^{G58R};OMA1$^{+/-}$	6	5
C10^{G58R};OMA1$^{-/-}$	4	7

B

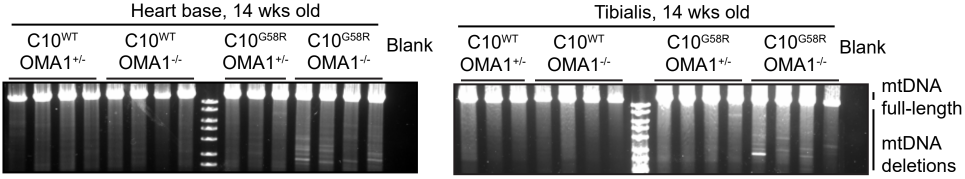

C

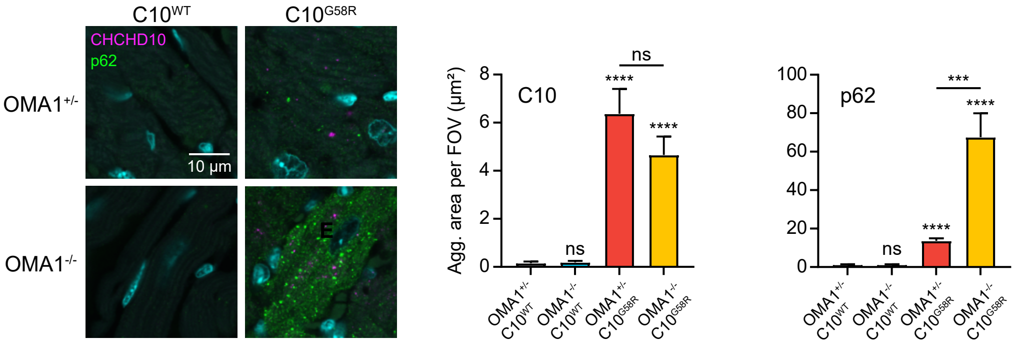

D

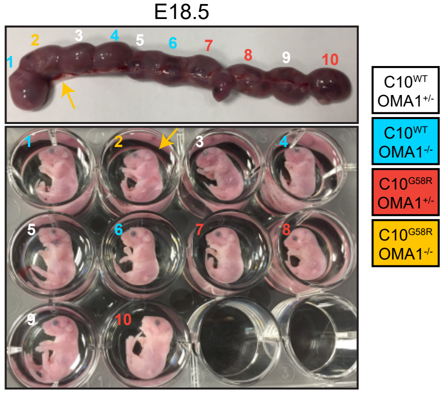

E

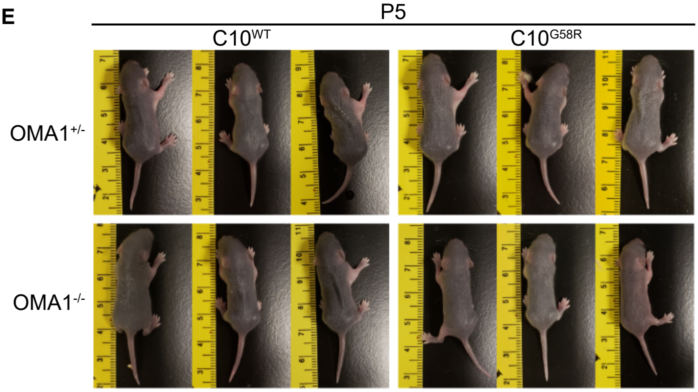

F

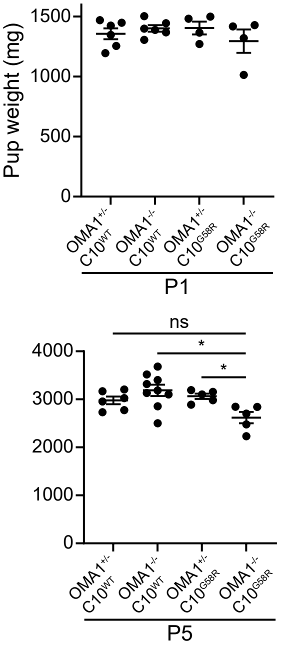

G

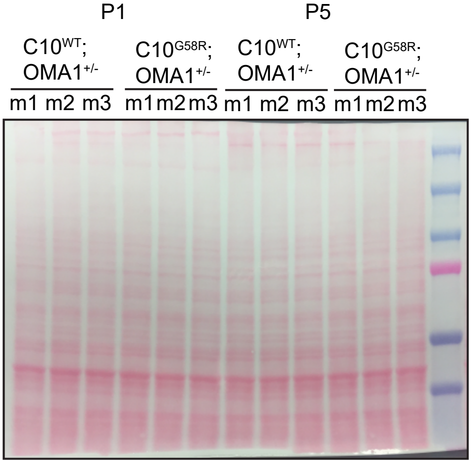

H

Genotypes at weaning

Genotype	Exp	Obs
C10^{WT};OMA1$^{+/-}$	17	18
C10^{WT};OMA1$^{-/-}$	17	15
C10^{S59L};OMA1$^{+/-}$	17	21
C10^{S59L};OMA1$^{-/-}$	17	14

$p = 0.6226$

Figure S9. OMA1 is critical for survival of C10 G58R mouse pups.

(A) Genotypes of pups from the $C10^{WT}$; $OMA1^{-/-}$ X $C10^{G58R}$; $OMA1^{+/-}$ cross at P1 and P5. (B) Long-range PCR of a 12.8kb segment of mtDNA from 14-week-old mouse hearts (left) and tibiales (right). DNA ladder is HyperLadder 1kb. Each lane represents an individual mouse. (C) Left: representative confocal images showing C10 and p62 staining of 14-week-old mouse hearts. Right: quantification of C10 and p62 aggregate area, (n = 3 mice per genotype for $OMA1^{+/-}$; $C10^{WT}$ and $OMA1^{+/-}$; $C10^{G58R}$, and n = 4 mice per genotype for and $OMA1^{-/-}$; $C10^{WT}$ and $OMA1^{-/-}$; $C10^{G58R}$. 10 FOV quantified per mouse). (D) E18.5 mouse embryos from the $C10^{WT}$; $OMA1^{-/-}$ X $C10^{G58R}$; $OMA1^{+/-}$ cross in the uterus (top) and outside of the uterus (bottom). The $OMA1^{-/-}$; $C10^{G58R}$ embryo is indicated. (E) P5 pups from the $C10^{WT}$; $OMA1^{-/-}$ X $C10^{G58R}$; $OMA1^{+/-}$ cross. (F) P1 and P5 pup weights from the $C10^{WT}$; $OMA1^{-/-}$ X $C10^{G58R}$; $OMA1^{+/-}$ cross. (G) Loading control for Figure 7D. (H) Expected vs. observed numbers of pup genotypes from the $C10^{WT}$; $OMA1^{-/-}$ X $C10^{S59L}$; $OMA1^{+/-}$ cross.

Supplemental Figure 10

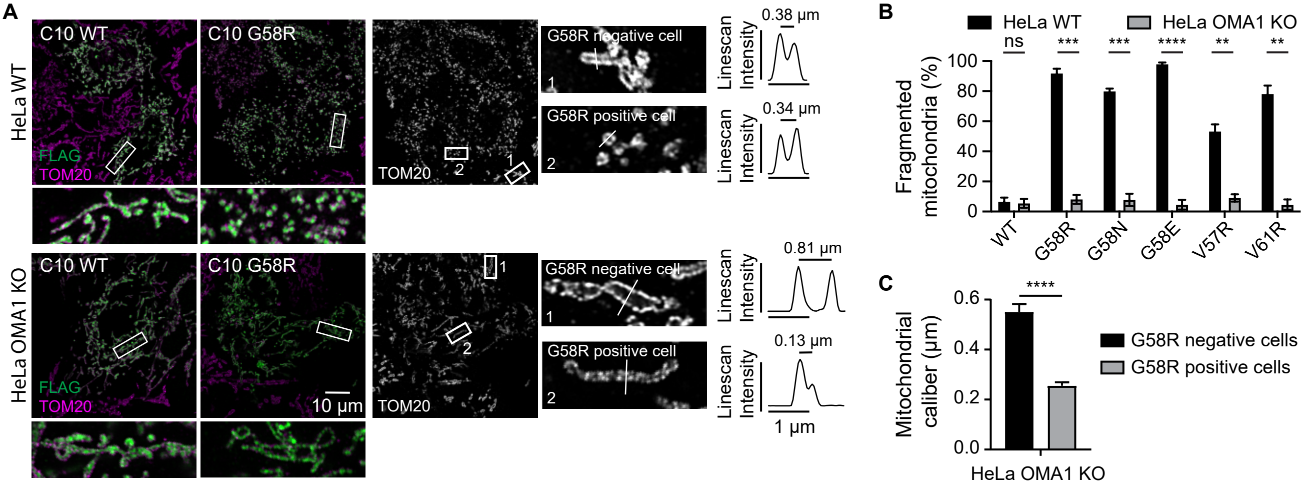

Figure S10. G58 substitutions fragment mitochondria through OMA1.

(A) HeLa cells transfected with C10 WT and C10 G58R constructs on the WT (top) or OMA1 KO (bottom) background. Magnifications depict mitochondrial caliber, with TOM20 line scan intensities on the right. Images were taken with a Zeiss Airyscan microscope. (B) Mitochondrial fragmentation levels in HeLa cells transfected with C10 mutants on the WT or OMA1 KO background ($n \geq 50$ mitochondria per replicate in 3 biological replicates per condition). (C) Quantification of mitochondrial caliber in (A), ($n = 42$ mitochondria from 3 biological replicates).

Supplemental Figure 11

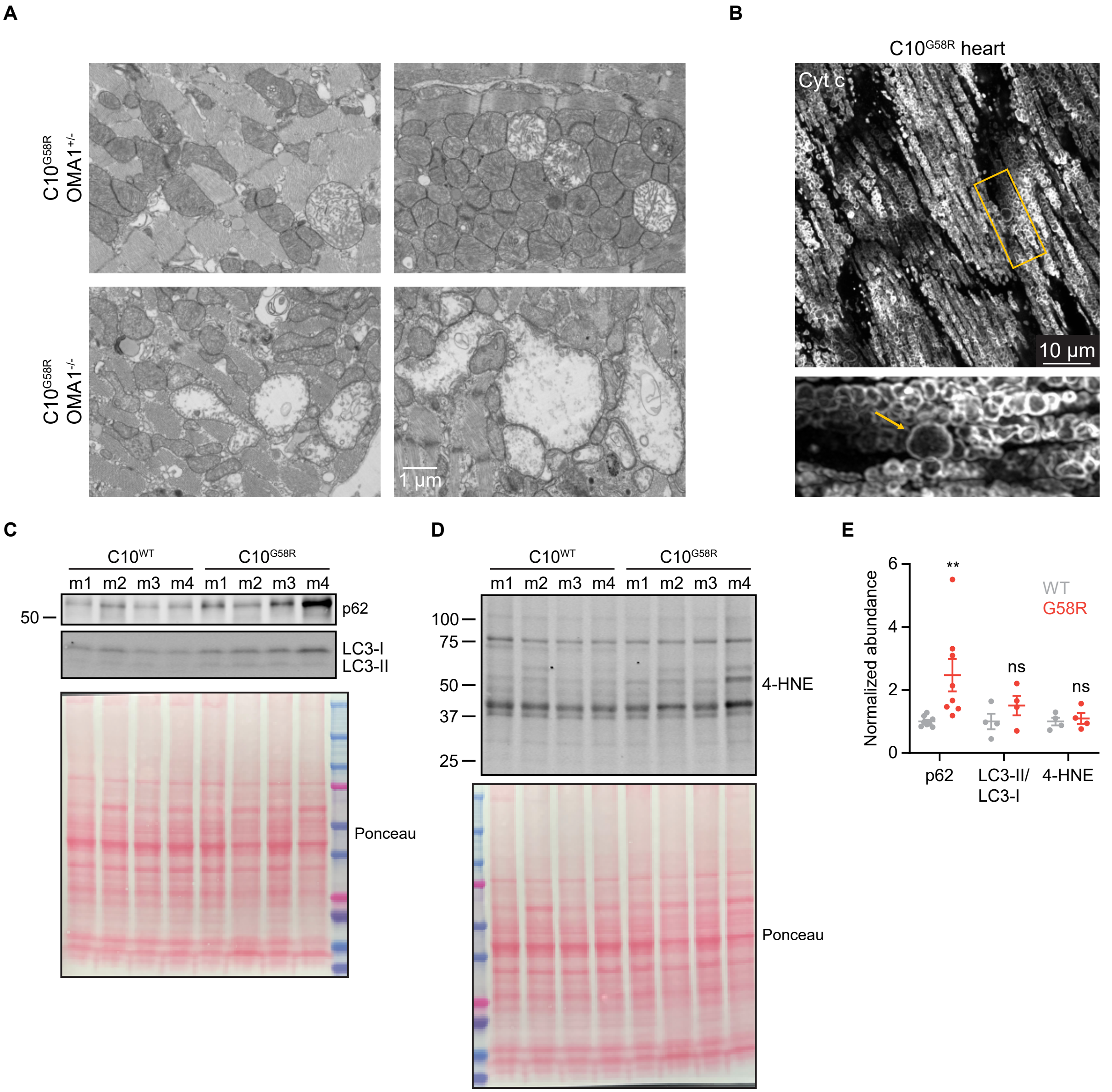

Figure S11. Megamitochondria, autophagy, and oxidative damage assessments in C10 G58R mouse hearts.

(A) Representative TEM images of megamitochondria. (B) 15-week-old $C10^{G58R}$ heart immunofluorescence showing a megamitochondrion. (C) Western blot of p62, LC3-I, and LC3-II from mouse heart lysates. (D) 4-HNE staining of mouse heart lysates. (E) Quantification of the data in (C) and (D).

Supplemental Figure 12

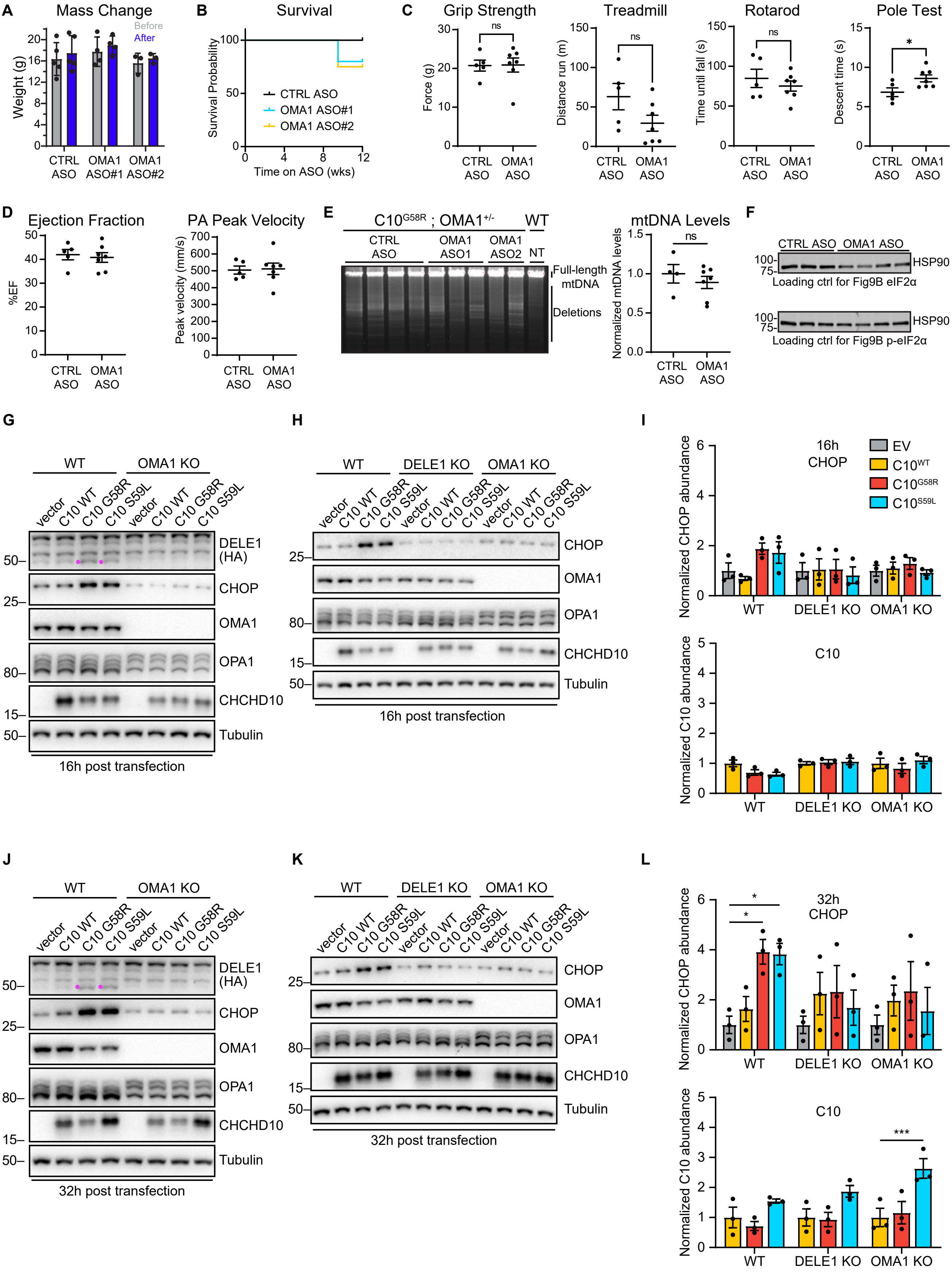

Figure S12. Pathogenic C10 mutations activate the ISR through the OMA1-DELE1 pathway.

(A) Weight of $C10^{G58R}$; $OMA1^{+/-}$ mice before the first ASO injection and after the last injection. (B) Survival of $C10^{G58R}$; $OMA1^{+/-}$ mice over the course of ASO treatment, (n = 5 mice for CTRL and OMA1 ASO#1, and n = 4 mice for OMA1 ASO#2). (C) Grip strength, treadmill, rotarod, and pole test results of 33-week-old $C10^{G58R}$; $OMA1^{+/-}$ mice after 12 weeks of CTRL ASO or OMA1 ASO. (D) Percent ejection fraction and pulmonary artery peak velocity of 33-week-old $C10^{G58R}$; $OMA1^{+/-}$ mice after 12 weeks of CTRL ASO or OMA1 ASO. (E) Left: long-range PCR of a 12.8kb segment of mtDNA from hearts of 33-week-old $C10^{G58R}$; $OMA1^{+/-}$ mice after 12 weeks of CTRL ASO or OMA1 ASO. Right: mtDNA copy number. (F) Loading controls for Figure 9B. Effect of 16-hour (G) and 32-hour (J) C10 WT, C10 G58R, and C10 S59L overexpression on DELE1 processing into S-DELE1 (pink) in HEK293T WT and OMA1 KO cells stably expressing DELE1-HA. Effect of 16-hour (H) and 32-hour (K) C10 WT, C10 G58R, and C10 S59L overexpression on CHOP levels and OPA1 processing in HEK293T cells on the WT, DELE1 KO, or OMA1 KO background. (I) and (L) are quantifications of (H) and (K), respectively. All comparisons are vs. the empty vector (for CHOP quantifications) or $C10^{WT}$ (for C10 quantifications).

Supplemental Figure 13

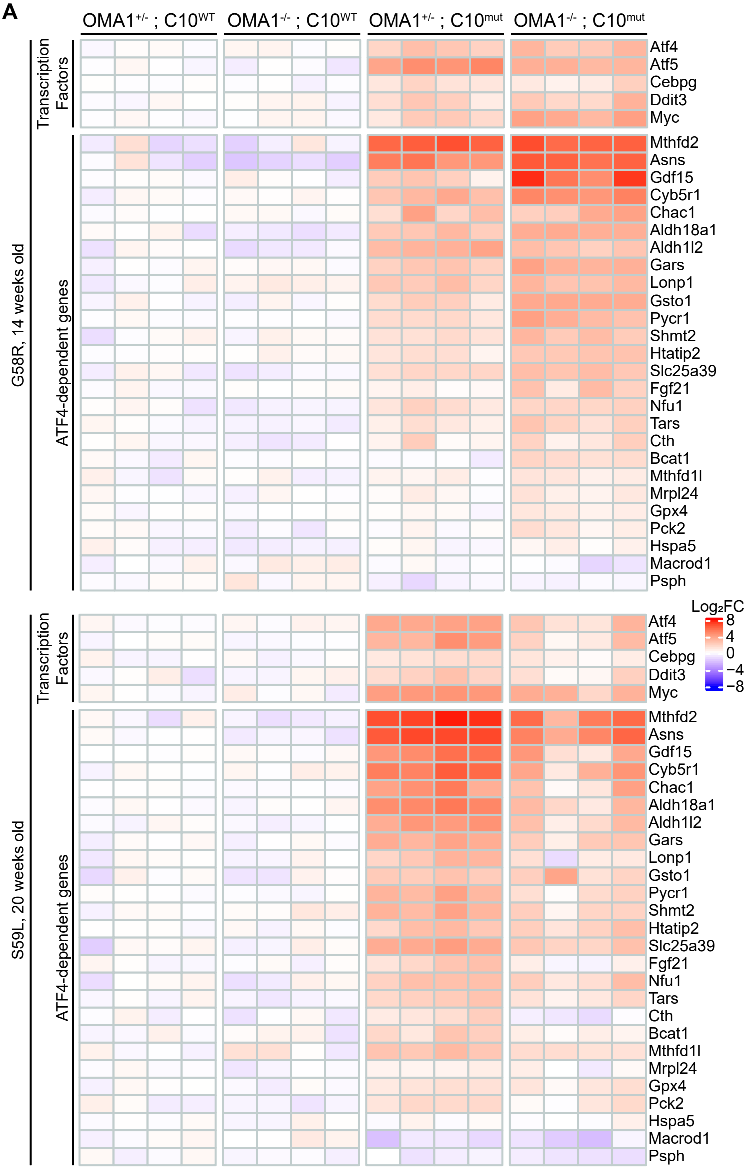

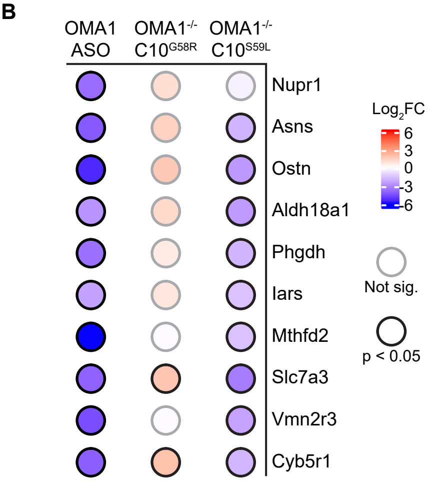

Figure S13. Transcriptomic analyses of C10 mutant mouse hearts.

(A) Microarray data of hearts from mice of the indicated genotypes. Each column represents a mouse. (B) Foldchange of the top 10 DEGs of the OMA1 ASO vs CTRL ASO comparison in said comparison, the $C10^{G58R}$; $OMA1^{-/-}$ vs $C10^{G58R}$; $OMA1^{+/-}$ comparison, and the $C10^{S59L}$; $OMA1^{-/-}$ vs $C10^{S59L}$; $OMA1^{+/-}$ comparison.

Supplemental Figure 14

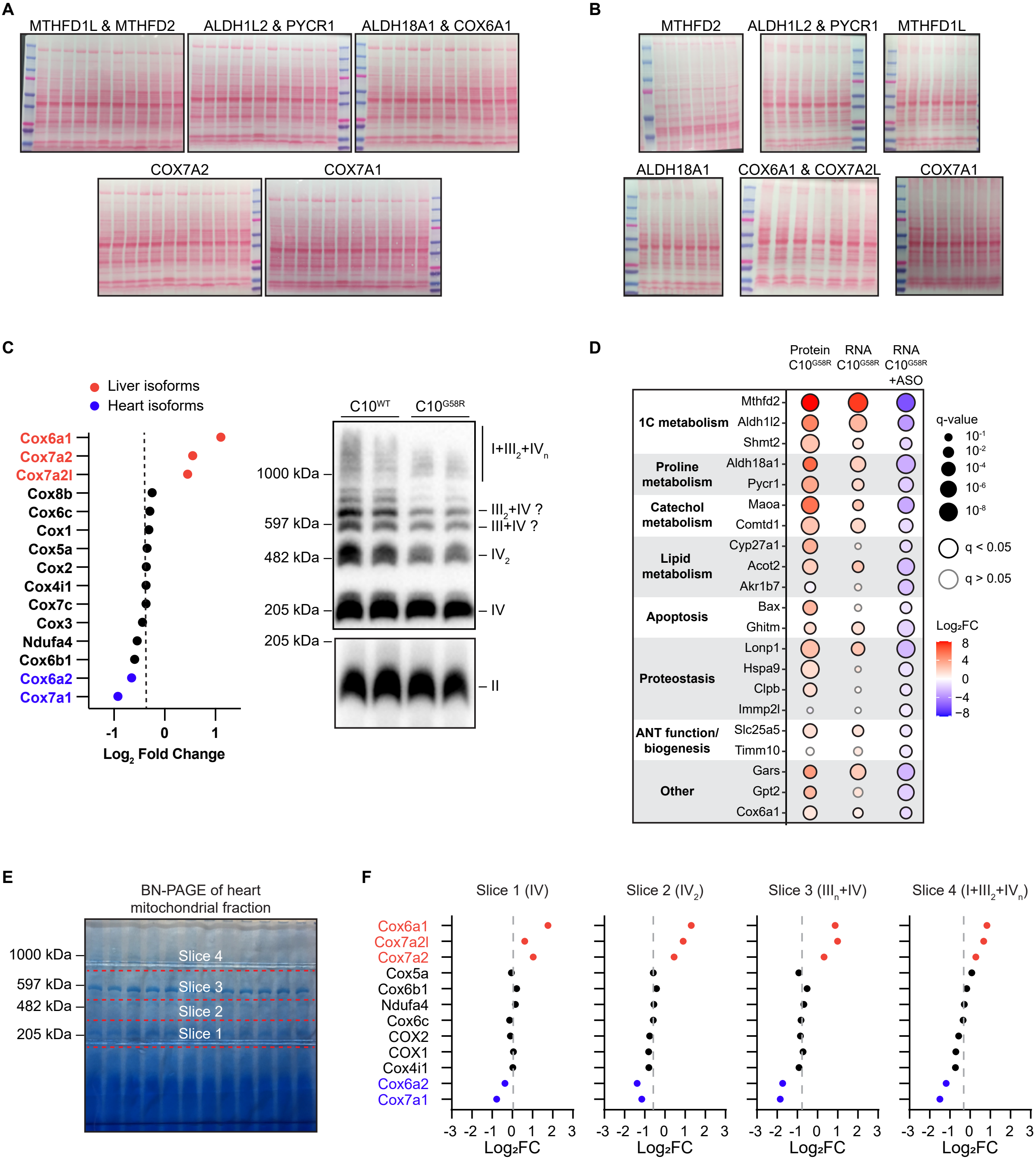

Figure S14. Effects of C10 G58R and S59L on the mitochondrial proteome.

(A) Loading controls for Figure 11B. (B) Loading controls for Figure 11C. (C) Left: foldchange of complex IV subunits from the $C10^{G58R}$ vs $C10^{WT}$ heart mitochondrial proteomics experiment in Figure 11A. Right: BN-PAGE gel of heart mitochondrial lysate of 36-week-old $C10^{WT}$ and $C10^{G58R}$ mice, stained for complex IV (top) and complex II (bottom). (D) Comparison of proteomic data from $C10^{G58R}$ vs $C10^{WT}$, transcriptomic data from $C10^{G58R}$ vs $C10^{WT}$ on the $OMA1^{+/-}$ background, and transcriptomic data from $OMA1^{+/-}$; $C10^{G58R}$ OMA1 ASO vs CTRL ASO for high-confidence OMA1 targets captured by proteomics. (E) BN-PAGE of $C10^{WT}$ and $C10^{G58R}$ heart mitochondrial lysates showing where the gel was cut for downstream proteomics. (F) Fold changes of CIV subunits between $C10^{WT}$ and $C10^{G58R}$ heart mitochondrial lysates in the different BN-PAGE slices from (E). Liver isoforms are in red and heart isoforms in blue. The dashed line indicates the median FC, (n = 3 mice per group).

Supplemental Figure 15

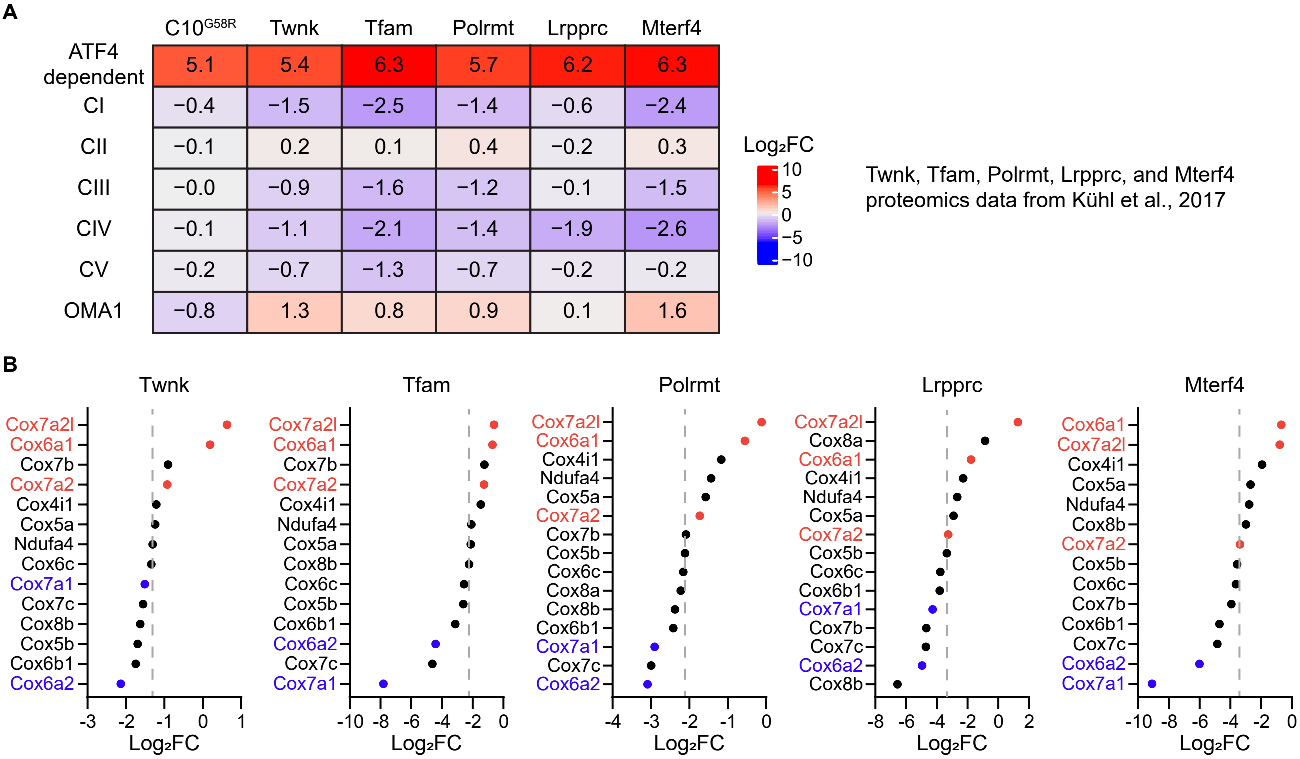

Figure S15. Effects of different models of mitochondrial stress on the mitochondrial proteome.

(A) Protein fold changes vs WT of ATF4-dependent proteins, CI-V subunits, and OMA1 in C10^{G58R} and different models of mitochondrial disease, resulting from heart KO of the following genes: Twnk, Tfam, Polrmt, Lrpprc, and Mterf4. Data is from Kühl et al., 2017. (B) Fold changes of CIV subunits in models in (A). Data is from Kühl et al., 2017. Liver isoforms are in red and heart isoforms in blue. The dashed line indicates the median FC.

Uncut gels

Lanes used in figures indicated. If no specific lanes are indicated then every lane was used in the figure.

Fig. 2E

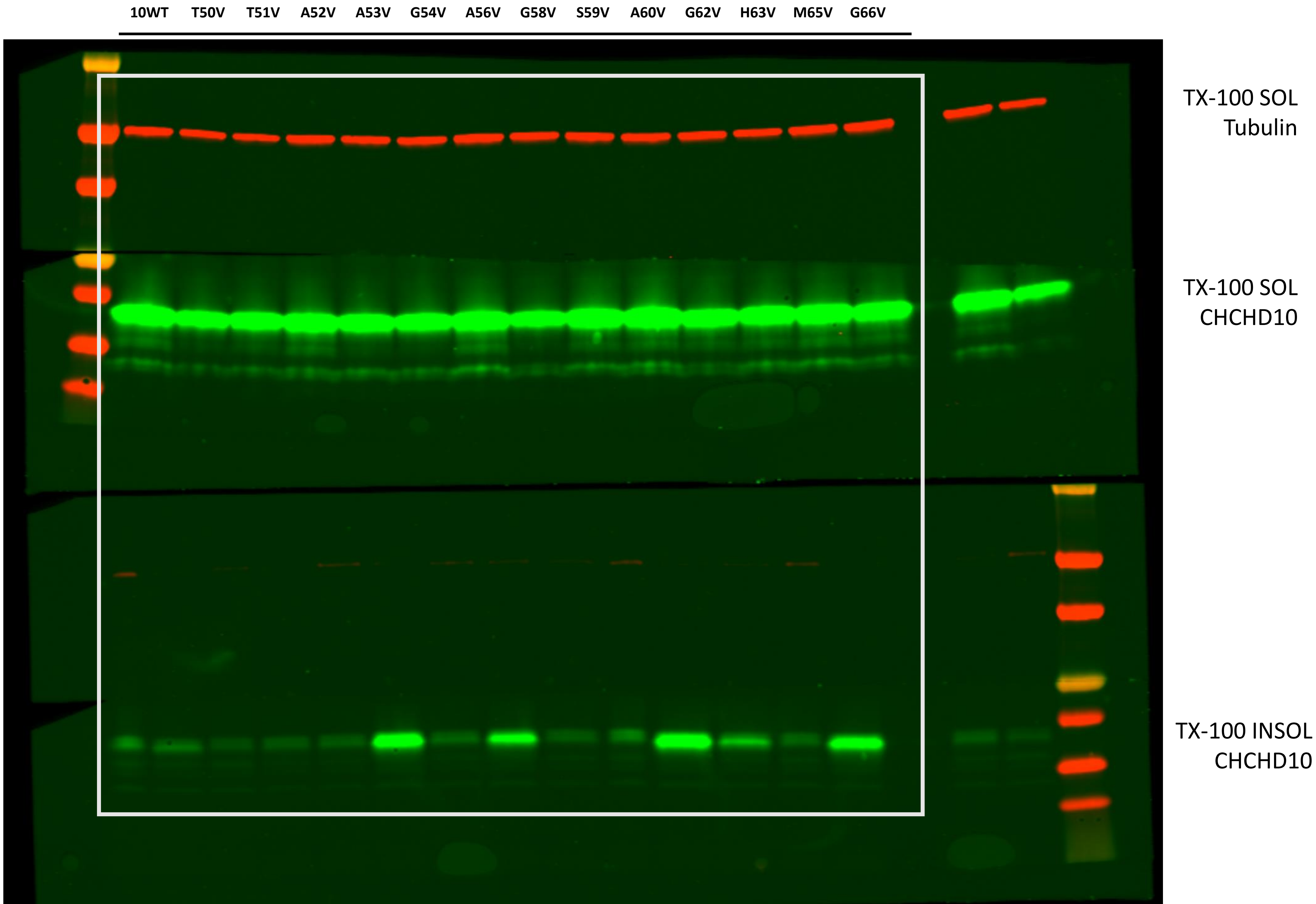

10WT
T50V
T51V
A52V
A53V
G54V
A56V
G58V
S59V
A60V
G62V
H63V
M65V
G66V
TX-100 SOL
Tubulin
TX-100 SOL
CHCHD10
TX-100 INSOL
CHCHD10

Fig. 3A

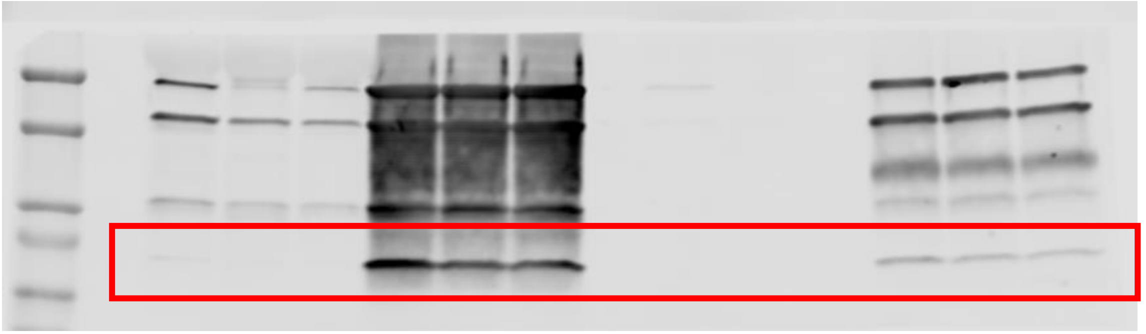

NDUFB8 (Total OXPHOS rodent)

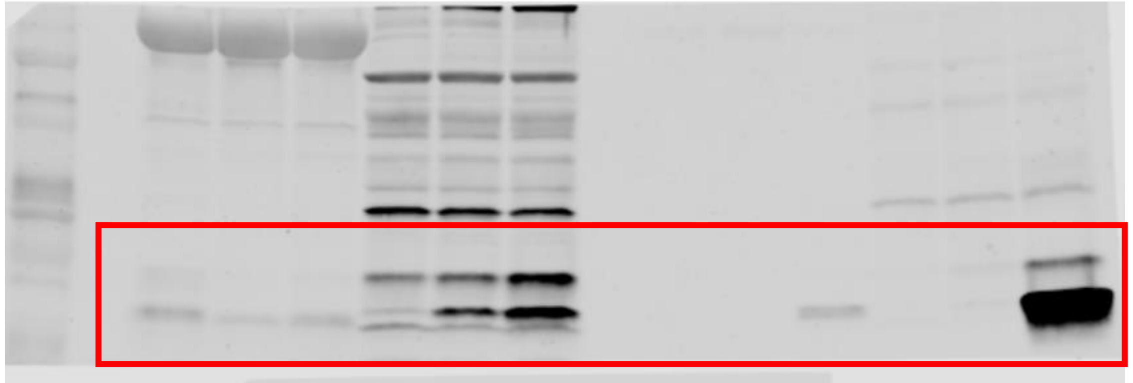

CHCHD2 rab

Fig. 5A

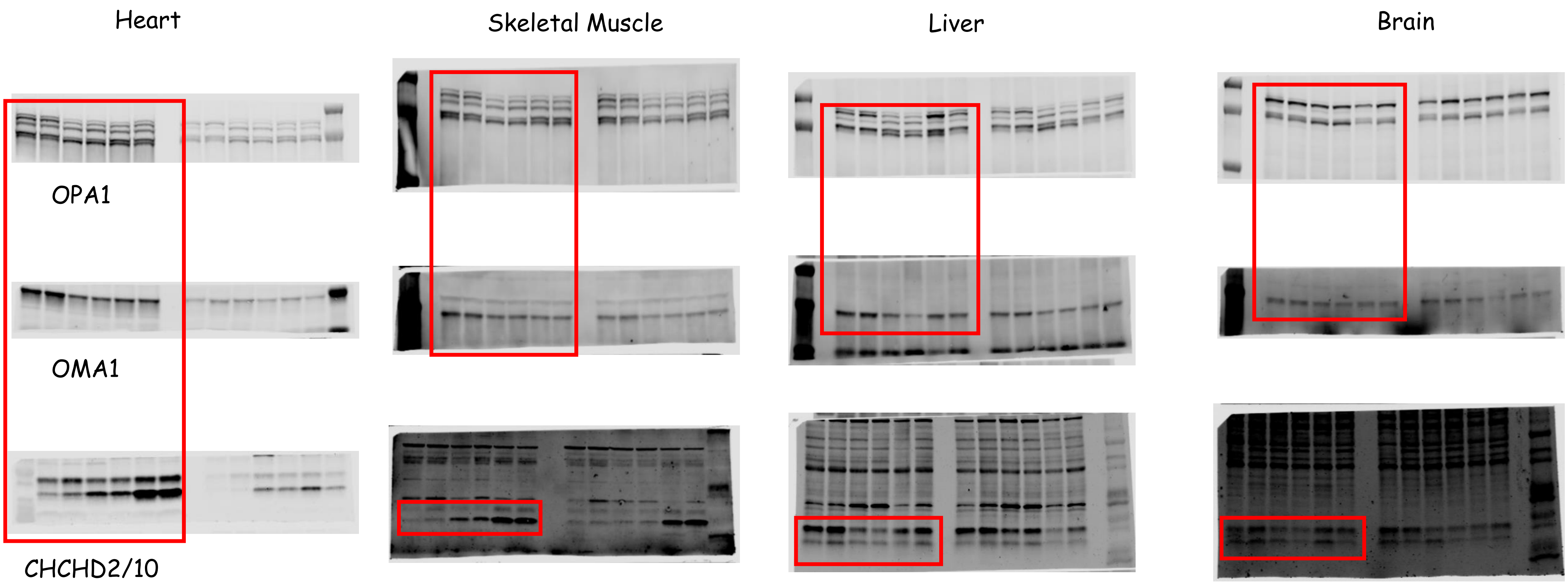

Fig. 5D

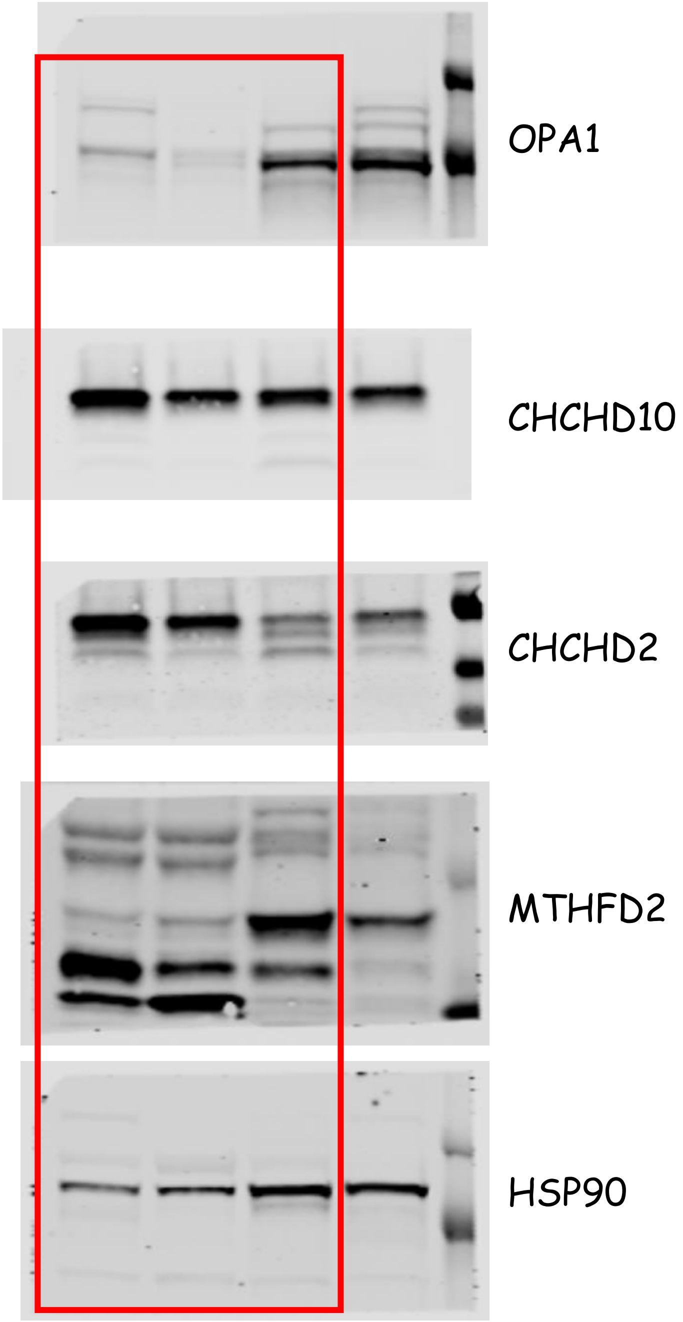

Fig. 6A

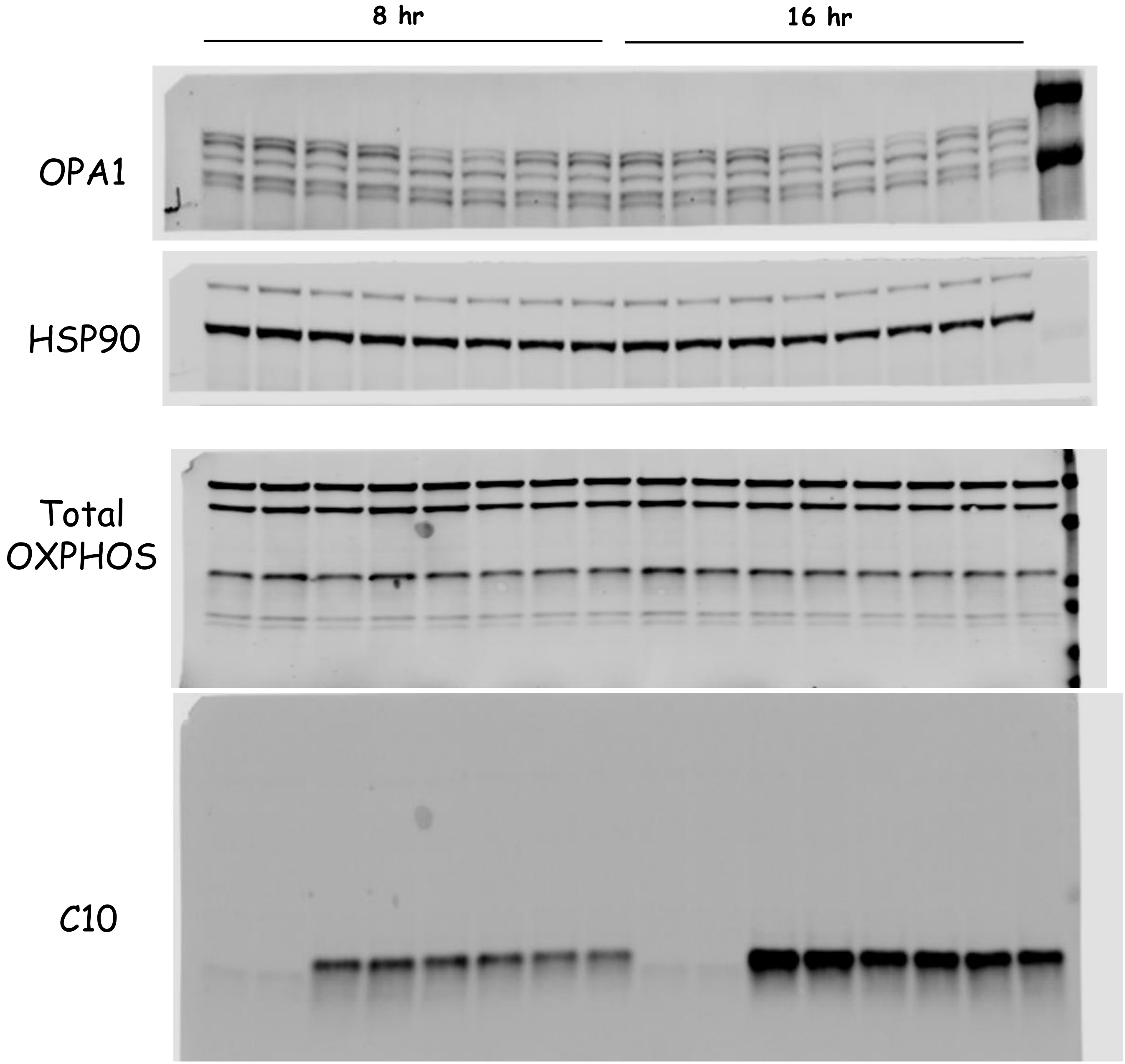

Fig. 7A

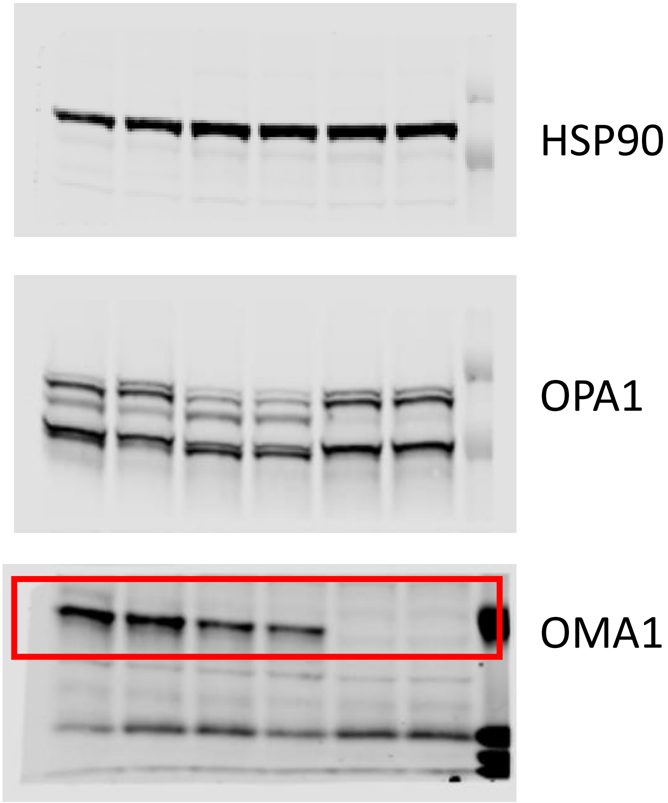

Fig. 7D

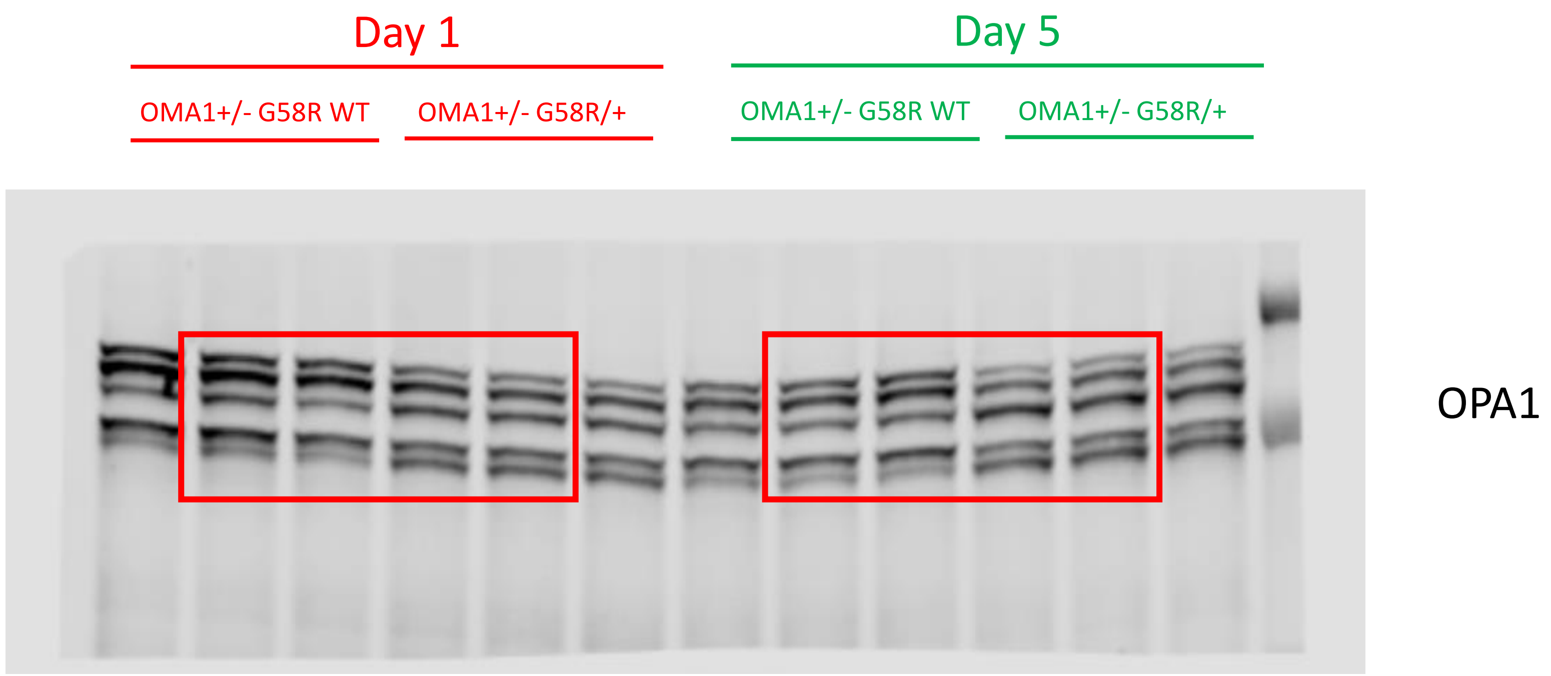

Fig. 9A

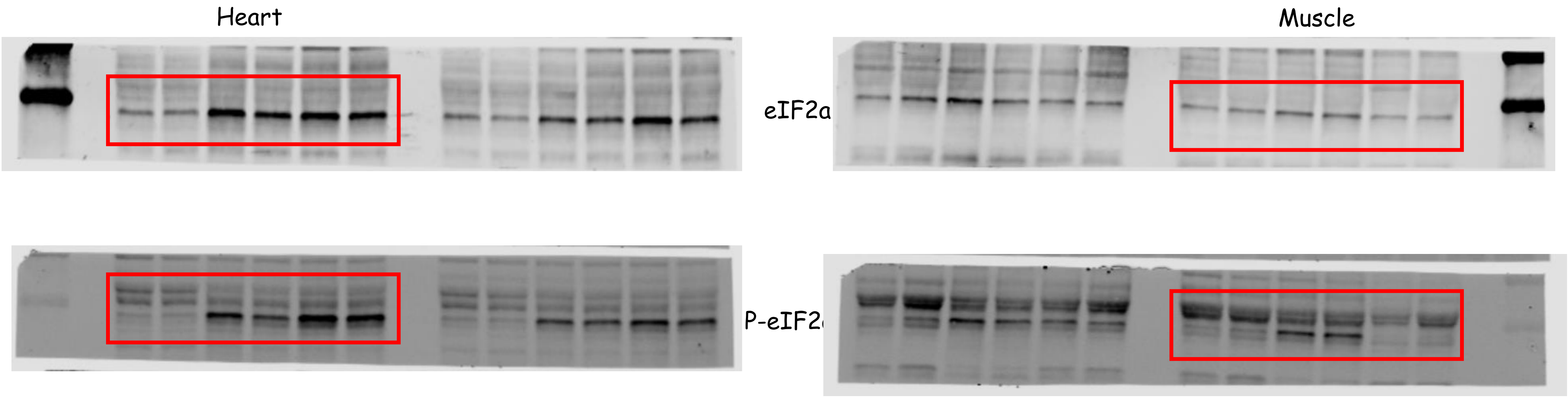

Fig. 9B

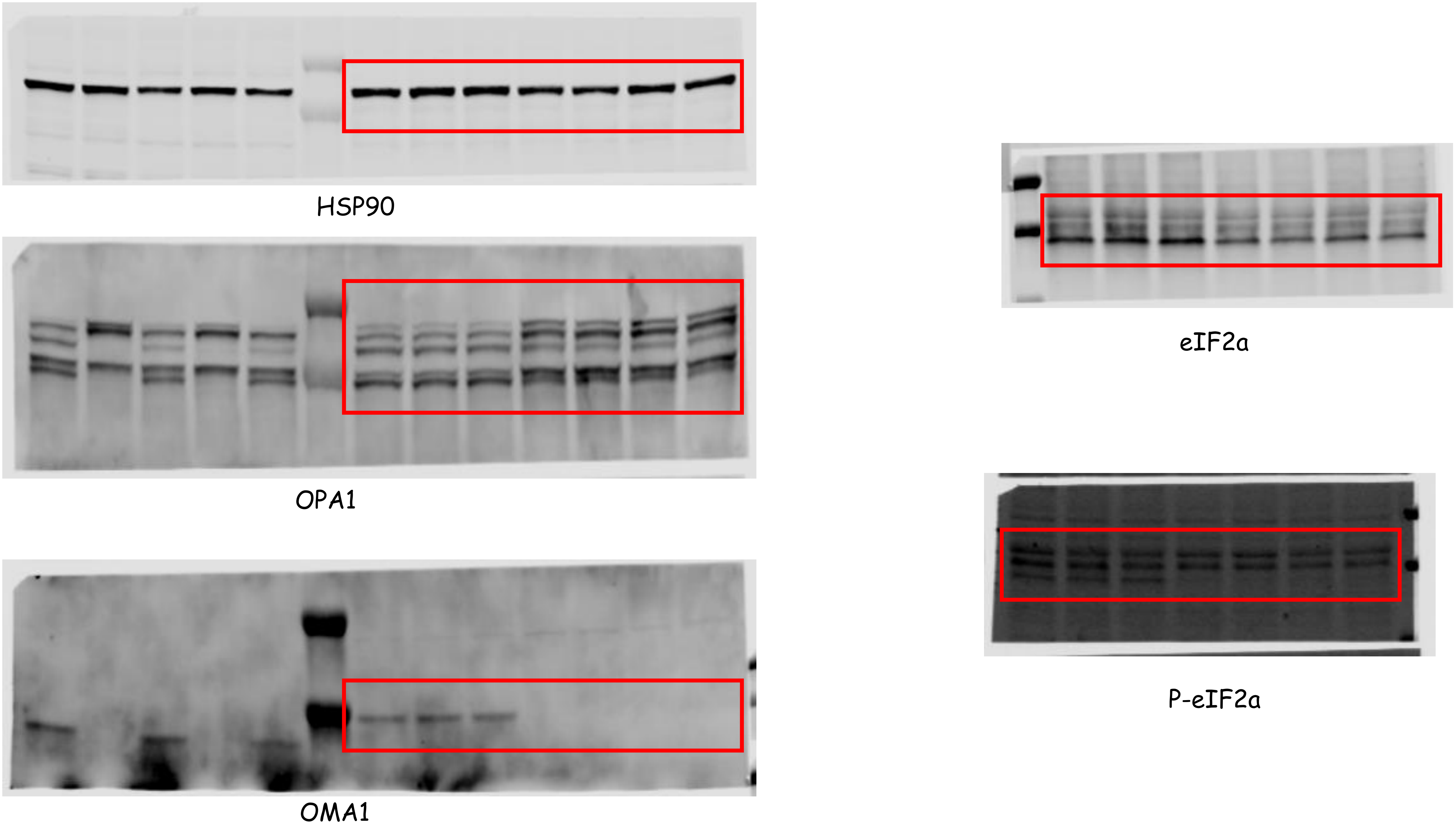

Fig. 11B

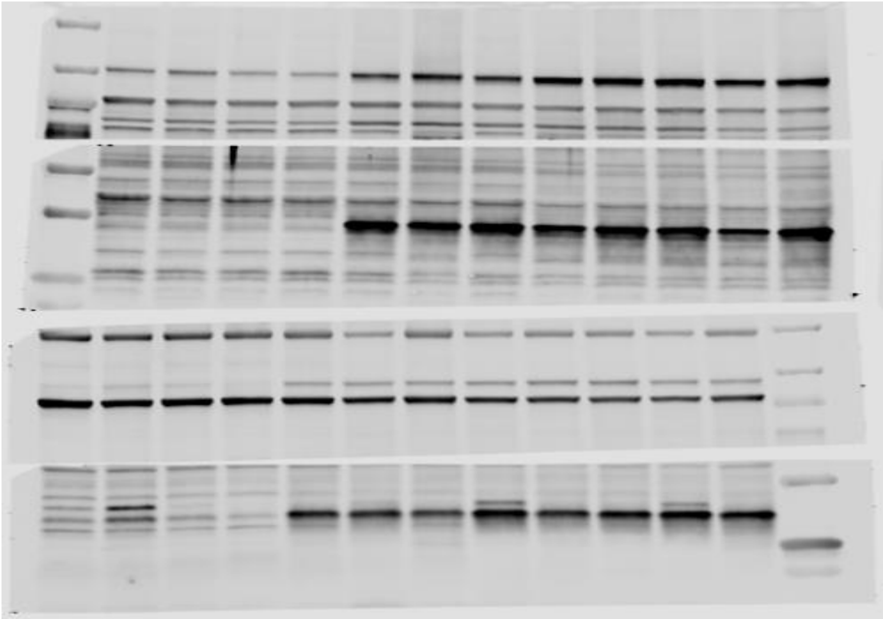

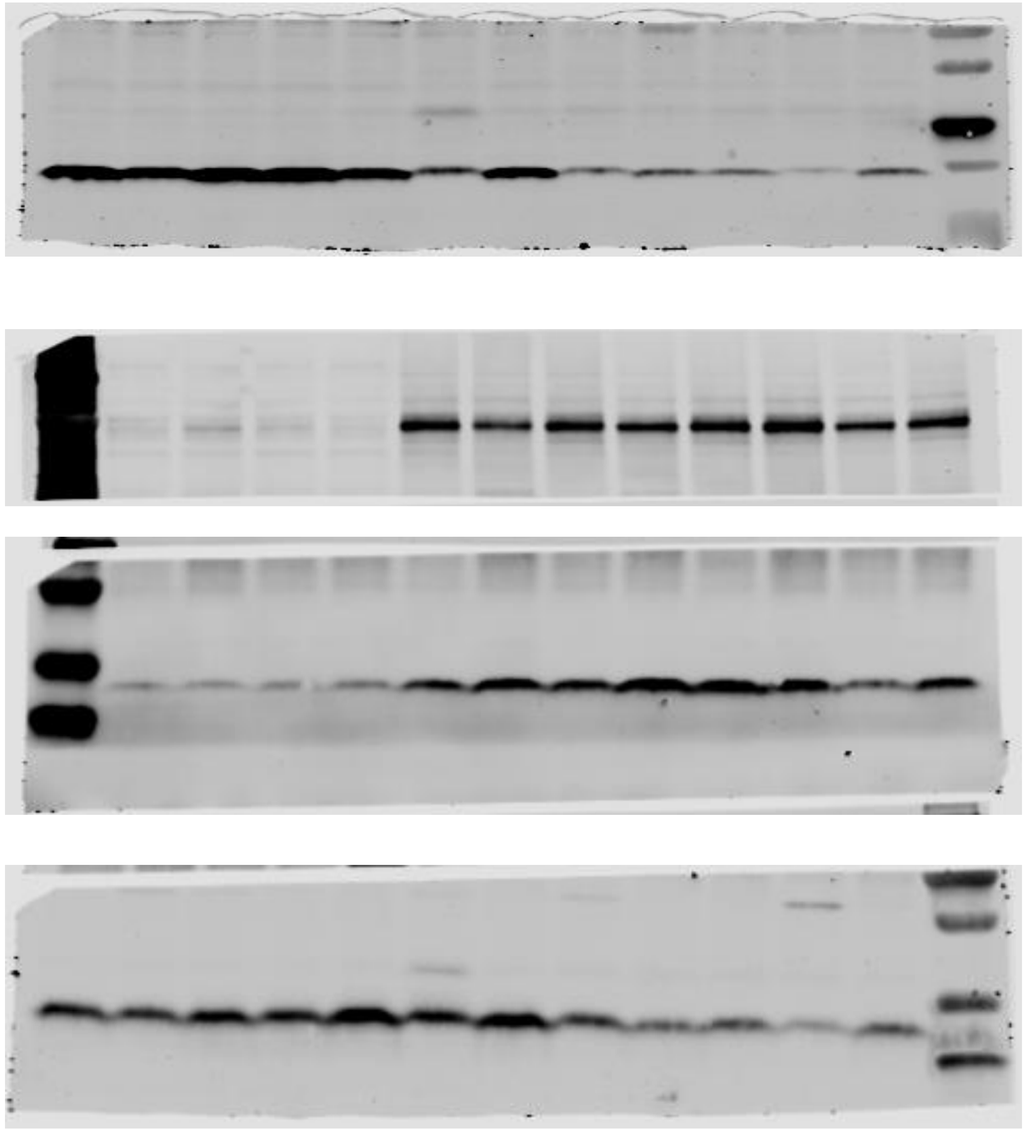

Fig. 11C

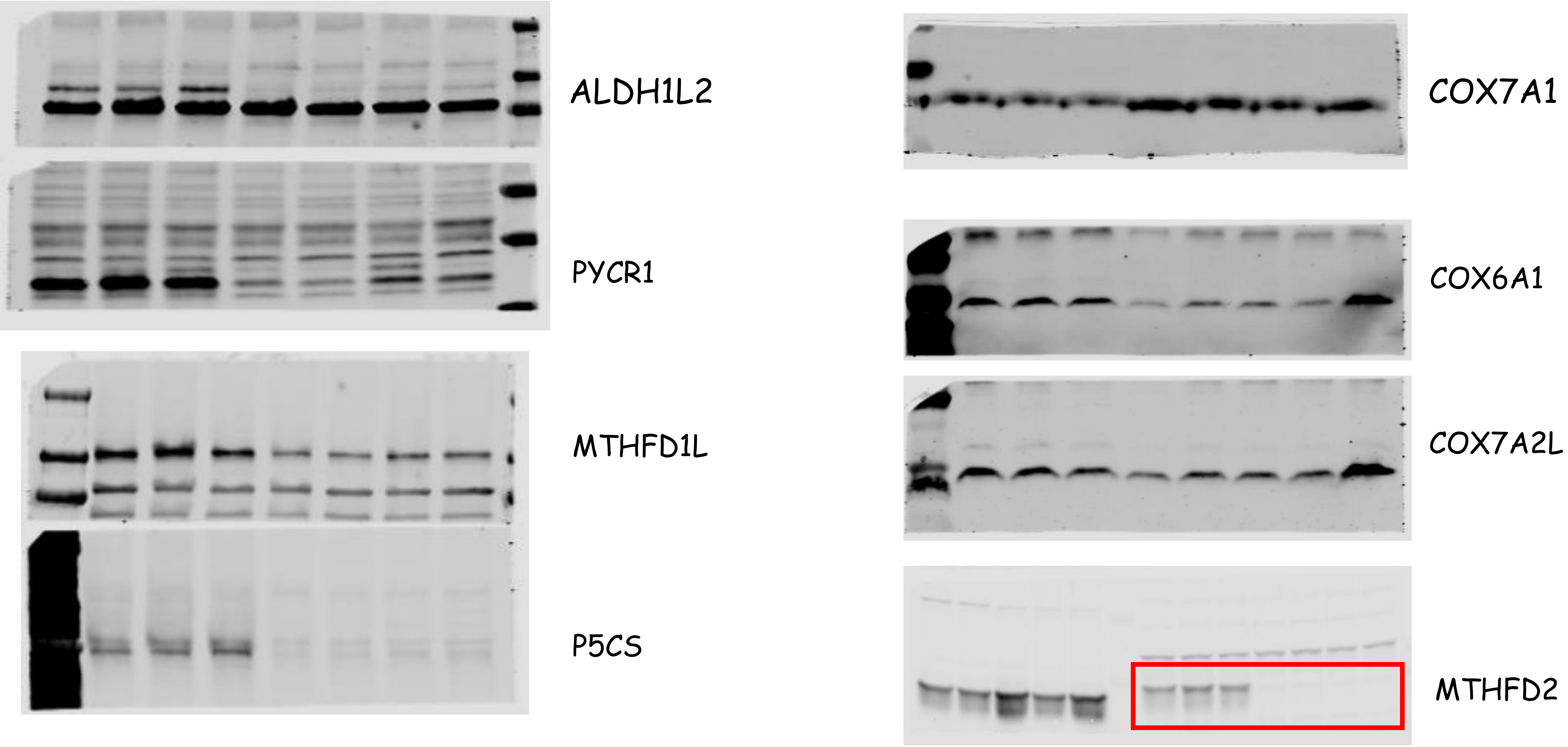

Supplement: Supplemental data [file jci-132-157504-s084.pdf]
